# Medications for community pharmacists to dose adjust or avoid to enhance prescribing safety in individuals with advanced chronic kidney disease: a scoping review and modified Delphi

**DOI:** 10.1186/s12882-024-03829-y

**Published:** 2024-10-29

**Authors:** Jo-Anne Wilson, Natalie Ratajczak, Katie Halliday, Marisa Battistella, Heather Naylor, Maneka Sheffield, Judith G. Marin, Jennifer Pitman, Natalie Kennie-Kaulbach, Shanna Trenaman, Louise Gillis

**Affiliations:** 1 College of Pharmacy, Faculty of Health, Dalhousie University, Halifax, NS Canada; 2 Leslie Dan Faculty of Pharmacy, University of Toronto, Toronto, ON Canada; 3 Clinical Pharmacist-Nephrology, University Health Network, Toronto, ON Canada; 4 Regional Research and Education, Horizon Health Network, St. John, New Brunswick Canada; 5 College of Pharmacy, Dalhousie University, St. John, New Brunswick Canada; 6 Nova Scotia Health Renal Program, Halifax, NS Canada; 7 St. Paul’s Hospital, Kidney Care Clinic, Vancouver, BC Canada; 8 UBC Pharmaceutical Sciences, Vancouver, BC Canada; 9 Pharmacy Department, Nova Scotia Health, Halifax, NS Canada; 10 Nova Scotia Health Research and Innovation, Halifax, NS Canada; 11 W.K. Kellogg Health Sciences Library, Dalhousie University, Halifax, NS Canada

**Keywords:** Medication Safety, Chronic Kidney Disease, Community Pharmacy Practice, Education

## Abstract

**Background:**

Community pharmacists commonly see individuals with chronic kidney disease (CKD) and are in an ideal position to mitigate harm from inappropriate prescribing. We sought to develop a relevant medication list for community pharmacists to dose adjust or avoid in individuals with an estimated glomerular filtration rate (eGFR) below 30 mL/min informed through a scoping review and modified Delphi panel of nephrology, geriatric and primary care pharmacists.

**Methods:**

A scoping review was undertaken to identify higher risk medications common to community pharmacy practice, which require a dose adaptation in individuals with advanced CKD. A 3-round modified Delphi was conducted, informed by the medications identified in our scoping review, to establish consensus on which medications community pharmacists should adjust or avoid in individuals with stage 4 and 5 CKD (non-dialysis).

**Results:**

Ninety-two articles and 88 medications were identified from our scoping review. Of which, 64 were deemed relevant to community pharmacy practice and presented for consideration to 27 panel experts. The panel consisted of Canadian pharmacists practicing in nephrology (66.7%), geriatrics (18.5%) and primary care (14.8%). All participants completed rounds 1 and 2 and 96% completed round 3. At the end of round 3, the top 40 medications to adjust or avoid were identified. All round 3 participants selected metformin, gabapentin, pregabalin, non-steroidal anti-inflammatory drugs, nitrofurantoin, ciprofloxacin and rivaroxaban as the top ranked medications.

**Conclusion:**

Medications eliminated by the kidneys may accumulate and cause harm in individuals with advanced chronic kidney disease. This study provides an expert consensus of the top 40 medications that community pharmacists should collaboratively adjust or avoid to enhance medication safety and prescribing for individuals with an eGFR below 30 mL/min.

**Supplementary Information:**

The online version contains supplementary material available at 10.1186/s12882-024-03829-y.

## Background

Chronic kidney disease (CKD) is common affecting nearly 4 million Canadians or 1 out of 10 people [1]. In Canadian primary care practices, the prevalence of CKD is higher in rural settings compared to urban settings [2]. Multiple comorbidities, advanced age and polypharmacy are common in individuals with CKD [3]. As kidney function declines with CKD, drug pharmacokinetics and pharmacodynamics are altered [4]. Medications eliminated by the kidneys may accumulate and cause harm. Considering these factors the risk for adverse drug events is high in this population [5–7]. Although drug dosing resources are available, inappropriate medication prescribing in individuals with lower kidney function is common in primary care and has been associated with adverse health outcomes [8–12]. In a recent study, the incidence rate of serious adverse drug reactions was reported to be significantly higher in individuals with an estimated glomerular filtration rate (eGFR) < 30 ml/min/1.73m^2^ compared to those with eGFR ≥ 30 mL/min/1.73m^2^ [13].

Community pharmacists commonly see individuals with CKD and can mitigate harm from inappropriate prescribing. The pharmacotherapy assessment in chronic renal disease (PAIR) instrument study reported that 21% of drug therapy problems in community pharmacy were related to inappropriate use or use of a contraindicated medication [14]. A recent qualitative study interviewing community pharmacists revealed barriers to assessing kidney function, dosing, and prescribing in CKD [15]. These pharmacists emphasized the need for an evidence and expert informed drug dosing tool which would contain an up-to-date relevant list of common and or high-risk medications to adjust or avoid in primary care in individuals with eGFR < 30 mL/min.

A member of this research team in 2020 published a multidisciplinary modified Delphi of Canadian medications used routinely in primary care to be dose adjusted or avoided in individuals with an eGFR < 60 mL/min CKD (M.B) [16]. In 2023, the American Geriatrics Society presented updated Beer’s Criteria for potentially inappropriate medication use in older adults where dosages should be adjusted based on kidney function [12]. This study undertook a scoping review of research on adverse drug events among CKD patients in community care, and, a modified Delphi panel of nephrology, geriatric and primary care pharmacists. Our scoping review expands on the comprehensive findings of two reviews [12, 16]. The present modified Delphi included pharmacists who routinely manage medications for individuals with CKD, targeting community pharmacists, in contrast to the earlier modified Delphi study, which primarily involved physicians targeting primary care physicians [16]. We provide an updated medication list intended for community pharmacists to adjust or avoid in individuals with an eGFR < 30 mL/min. Ultimately, the list of medications will inform the development of an electronic drug dosing decision support tool for community pharmacists aimed to reduce inappropriate drug exposure and harm through appropriate prescribing.

## Methods

### Study design

This study consisted of two phases. In the first phase, a scoping review was undertaken to identify higher risk medications used in primary care, in particular community pharmacy practice, which require a dose adaptation in individuals with an eGFR less than 30 mL/min. In the second phase, we conducted a modified Delphi, informed by the medications identified in our scoping review, to establish consensus on which medications community pharmacists should adjust or avoid in individuals with stage 4 and 5 CKD (non-dialysis).

### Scoping review

In the first phase, a scoping review was conducted using the methodological framework outlined by Arksey and O’Malley and further developed by Levac and colleagues [17, 18]. In brief, we: *identified the research question, identified relevant studies, selected studies, charted and extracted data and summarized and reported results.* The study was registered with Open Science Framework and reported according to recommendations of the Preferred Reporting Items for Systematic Reviews and Meta-Analyses extension for Scoping Reviews (PRISMA-ScR) along with updated guidelines from Peters and colleagues [18–21].

The purpose of this project was to inform the development of a list of drugs for community pharmacists to dose adjust or avoid. The following research question guided the review: What drug related adverse effects are reported in the literature in individuals with advanced kidney disease (CKD stage 4 and CKD 5 non-dialysis dependent [eGFR < 30 ml/min/1.73m^2^])? To answer our research question, we employed a population (chronic kidney disease), concept (risk or development of adverse effects/reaction) and context (primary care or community pharmacist or any geographical content [drug related hospital admission]) framework [20]. To *identify relevant studies*, a search strategy using both controlled vocabulary and natural language was developed with a librarian and tested for retrieval performance using a set of known articles. It was then peer reviewed using the PRESS checklist [22]. We conducted searches in MEDLINE (Ovid), Embase (Elsevier), and CINAHL (Ebsco) databases on March 3, 2024. The final search strategy for these databases is provided [Additional file 1]. This review considered literature published from 2022 to 2024 to capture studies not identified from two previous reviews (up to May 31, 2022) with a focus on relevant medications used in primary care such as community pharmacy practice [12, 16]. To identify relevant sources not indexed in these databases, citation chaining of included studies was conducted. Grey literature was identified through targeted searches of the following organizational websites: Kidney Disease Improving Global Outcomes, National Kidney Foundation and Kidney Health Australia.

*Study selection* included literature published in English which described medication harm in adults with advanced CKD in the context of primary care setting. Studies were excluded if: they did not pertain to population of interest (dialysis, pediatrics, animals), the medication was not approved for use in Canada, it was administered by the intravenous route, it included non-prescription or more specialized medications (cancer therapies), and the publications were case series, reports, editorials, letters, and drug interaction studies. The article selection process was managed using Covidence Systematic Review Software. After removal of duplicates, two team reviewers independently screened title and abstracts for eligibility and a third team reviewer resolved any discrepancies (J.W, K.H, H.N). Full-text articles deemed eligible were screened by the same process as title and abstract screening. *Data extraction* of studies entailed utilizing a predefined template in Microsoft Excel and included the following: publication, author, date, country, objective or aim, study design, participants, setting, methodology, analysis, outcomes, relevant medication(s) to adjust or avoid. Data was extracted by team members and reviewed by 2 other members for verification, and consensus. Articles were *summarized* and categorized from all sources and according to whether it was sourced from a bibliographic database or through supplemental grey literature searching. Medications identified from the present scoping review and two previous reviews were combined [12, 16]. Investigators in nephrology, geriatrics and primary care engaged in discourse to formulate a list of medications to adjust or avoid relevant to community pharmacy practice which would be presented for consideration to our modified Delphi panel. The team aimed to identify medications for which community pharmacist would have the necessary competency to make medication adaptations, leading to the exclusion of less common specialized medications (e.g. cancer therapies).

### Modified Delphi

The modified Delphi was informed by the RAND methodological guidance and followed the Conducting and REporting DElphi Studies [23, 24]. A modified Delphi approach was used as the process ensures anonymity of responses thereby promoting consensus decision making without influence from dominant individuals. Three iterative rounds of modified Delphi survey were completed using an online secure survey tool (Opinio 7.5, Oslo, Norway) [25]. The survey was pre-tested by two pharmacists for content, clarity of questions and survey format with team members (J.W, M.S). Institutional Research Ethics Board approval was obtained for this study.

### Participants

Canadian pharmacists practicing in nephrology, geriatrics or primary care with five or more years of experience who routinely manage individuals with stage 4 and 5 CKD (non-dialysis dependent) were recruited. Purposeful sampling was employed by the research team to identify information-rich participants. In addition, snowball sampling was used, where participants could suggest other pharmacists to include with relevant expertise. Informed consent was obtained from pharmacists willing to participate. Target enrollment consisted of 20–30 pharmacist experts to capture perspectives from most Canadian provinces, reduce the potential of individual expert influences and to account for attrition. Participants received no financial compensation.

### Survey rounds

Three survey rounds were self-administered by participants, using a secure online link and were completed between June 26, 2024, and August 19, 2024. Participants had an average of 10 days to finish each round, and the survey took approximately 60 min to complete. Prior to each round, a team member (N.R) emailed participants with when to expect the survey link, provided survey instructions and a drug dosing resource of drugs for consideration in the rounds to assist with completing the survey questions [Additional file 2]. Each survey contained the rationale, purpose of the modified Delphi process and were divided into medication categories followed by individual medications [Additional file 3]. In round 1 for each medication, participants were asked to select from a drop down menu whether to dose adjust or avoid the medication based on eGFR category (15–29 mL/min and < 15 mL/min) and rate their level of agreement on a 5 point Likert scale (1 = strongly disagree, 2 = disagree, 3 = neither agree nor disagree, 4 = agree, 5 = strongly agree) on the importance of community pharmacists adjusting or avoiding the select medication. At the end of the survey, participants could add additional medications not included in the current round for consideration in round 2. In round 2, based on ratings from round 1, participants were asked to rate their top 45 medications which community pharmacists should dose adjust or avoid for eGFR < 30 mL/min. For newly added medications suggested from round 1 for round 2, they were again asked to select whether they would dose-adjust or avoid the medication and rate their level of agreement on the importance of community pharmacists adjusting or avoiding the select medication as they had done in round 1. In round 3, participants were asked to rate their top 40 medications which community pharmacists should dose adjust or avoid for eGFR < 30 mL/min. They also were asked to select whether they would dose adjust or avoid these medications along with providing a rating on their level of agreement for community pharmacists providing drug adaptations as they had done in the previous 2 rounds. Throughout the rounds, the only guidance provided to Delphi panel members was to consider the frequency of medication use and risk of potential medication harm in determining their ratings. Participants were also able to share comments in a free text box. At the end of each survey, they could include their email to receive a summary of their individual responses (i.e., dose adjust or avoid selection and ratings) through Opinio survey software to support the consensus building process with subsequent rounds. Only participants who completed all the questions in round 1 and round 2 were invited to take part in subsequent rounds.

### Data collection and analysis

Deidentified data from the surveys was collected by the Opinio software tool. After each survey round, participant selections (dose adjust or avoid) and ratings were collated. The percentages of participants who selected dose adjust or avoid per eGFR category for the medications was tabulated for rounds 1–3. We determined consensus for a medication to move on from round 1 to round 2 if it achieved a mean panel score greater than 3 from participants based on their rating on a 5-point Likert scale regarding the importance of community pharmacists dose adjusting or avoiding the medication for the eGFR category. Percentages of the top 45 and 40 medications were calculated for round 2 and 3, respectively. Descriptive statistics were used to report demographic and clinical characteristics of the panel. Qualitative data from the free text fields were analyzed for emerging themes where comments were reported more than twice.

## Results

### Scoping review

**Fig. 1:**
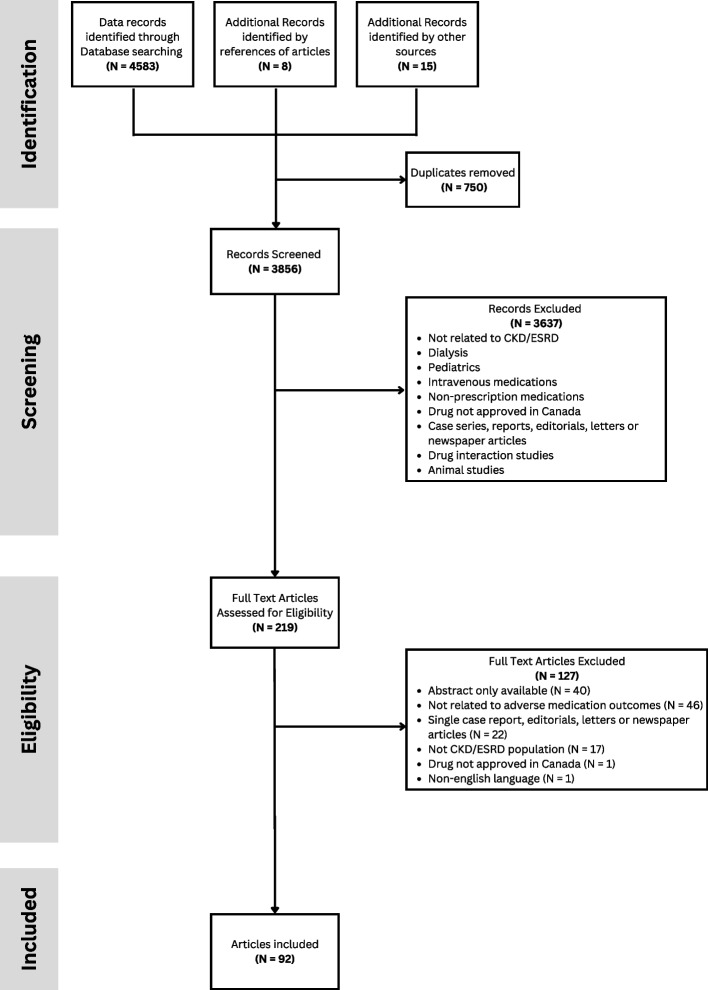
Preferred reporting items for systematic reviews and meta-analysis flowchart for study selection

Figure 1 outlines the PRISMA-ScR flow diagram for which we retrieved 4583 studies from the electronic database searches. Twenty-three additional records were identified through other sources: citations from included studies, and targeted searching of known kidney organization websites. After removing duplicates, 3856 articles were included for screening. Title and abstract screening using our inclusion criteria yielded 219 studies for full text review screening. Of these, 127 were excluded which led to the inclusion of 92 articles related to our research question. The two most common reasons for article exclusion were no adverse medication outcome reported or insufficient reporting detail (abstract only). From the 92 articles included, 88 medications were identified which were associated with harm requiring dose adjustment or avoidance. Based on relevance to community pharmacy practice and availability, consensus from the research team led to 64 medications being presented to for consideration by the Modified Delphi panel. Fifty-two of the 64 medications were identified from two previous reviews [Additional file 4] [12, 16]. Twelve were unique medications to our scoping review. Data extraction for the twelve unique medications are available [Additional file 5].

### Modified Delphi

Twenty-seven pharmacists in 8 of 10 provinces participated in the Modified Delphi panel (Table 1). The survey response rate was 100% of participants for round 1 and 2 and 96% (26/27) in round 3. The majority (81.4%) of participants were female. Nephrology was the most common participant area of practice (66.7%), with other pharmacist participants working in geriatrics (18.5%) and primary care (14.8%). The mean ± standard deviation (SD) for participant years of experience in their practice area was 10.9 ± 4.0 years. Most participants (55.6%) had a Bachelor of Science in Pharmacy as their highest academic qualifications. The mean ± SD number of individuals with CKD managed per month by participants was 34.4 ± 18.1.

**Table 1:** Modified Delphi Panel Characteristics

Characteristic	Participants *N* = 27
Sex, female, *n* (%)	22 (81.4)
Practice Location, *n* (%)	
New Brunswick	6 (22.2)
Nova Scotia	6 (22.2)
Ontario	4 (14.8)
British Columbia	3 (11.1)
Manitoba	3 (11.1)
Quebec	2 (7.4)
Saskatchewan	2 (7.4)
Prince Edward Island	1 (3.7)
Highest Academic Credential, *n* (%)	
Bachelor of Science in Pharmacy ± ACPR	15 (55.6)
Postgraduate PharmD	6 (22.2)
Master’s degree	5 (18.5)
Undergraduate PharmD	1 (3.7)
Area of Practice, *n* (%)	
Nephrology	18 (66.7)
Geriatrics	5 (18.5)
Primary Care	4 (14.8)
Area of Practice, years (mean ± SD)	10.9 ± 4.0
No. CKD Patients managed/month (mean ± SD)	34.4 ± 18.1

*ACPR* Accredited Canadian Pharmacy Residency, *CKD* Chronic Kidney Disease, *N* Number, *No.* Number of, *PharmD* Doctor of Pharmacy, *SD* Standard Deviation

**Fig. 2:**
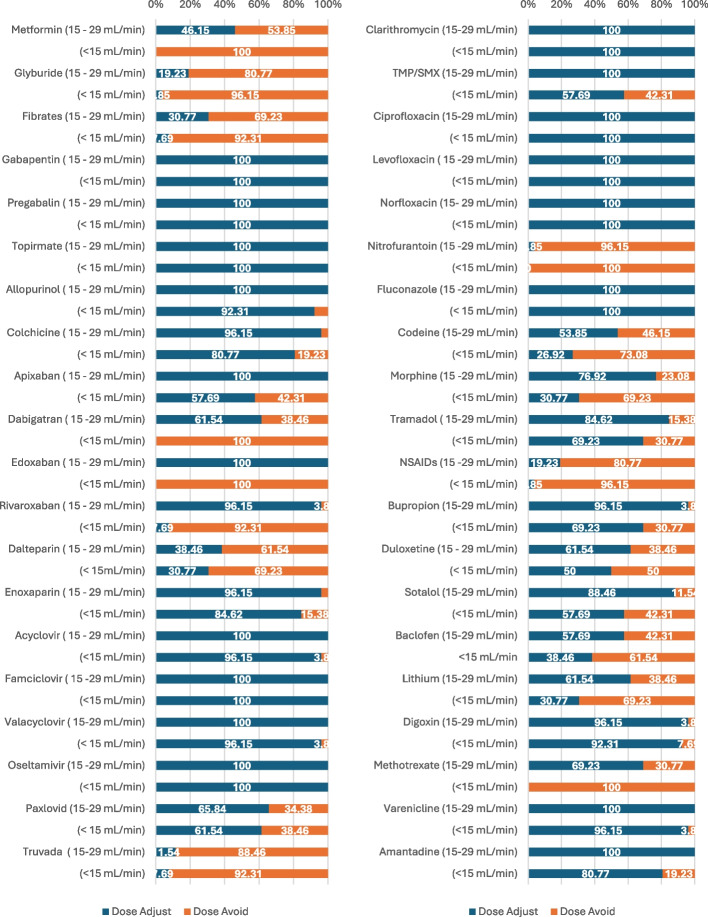
Modified Delphi panel selection of top 40 medications to dose adjust or avoid as percentages

Sixty-four medications were presented to participants in round 1 with an additional 5 medications added in round 2 of the modified Delphi process [Additional file 4]. In round 1, all participants selected to avoid nitrofurantoin for eGFR 15–29 and eGFR < 15 mL/min. For eGFR < 15 mL/min, all 27 pharmacists choose to avoid *metformin, dabigatran, bezafibrate, ubrogepant* as well as *nitrofurantoin*. Seventy-five percent and 89% of medications achieved a mean score ≥ 4 by participants who agreed or strongly agreed on importance of community pharmacists dose adjusting or avoiding the medication for eGFR 15–29 mL/min and eGFR < 15 mL/min, respectively. All 64 medications from round 1 achieved a mean panel score of > 3 by participants on the importance of community pharmacists to dose adjust or avoid the medication for both eGFR categories and were included in round 2. An additional 26 medications were suggested by pharmacist Delphi panel for inclusion in round 2. Of which, 5 were added based on relevance to community pharmacy practice and where more than two panel members suggested the medication. For eGFR 15–29 mL/min and eGFR < 15 mL/min (round 1 and 2), all participants choose to dose adjust 32 and 15 medications, respectively [Additional file 6]. At the end of round 2, the top 45 medications to dose adjust or avoid by community pharmacists were identified by the Delphi panel, which included a total of 47 medications, (due to ties) to be presented to round 3. At the end of round 3, the top 40 medications to dose adjust or avoid by community pharmacist were identified [Additional file 7]. All panelists selected *metformin, gabapentin, pregabalin, rivaroxaban, ciprofloxacin, nitrofurantoin, and non-steroidal anti-inflammatory drugs* as the top ranked medications. The percentages of participants who selected dose adjust or avoid per eGFR category for these 40 medications are outlined in Fig. 2.

**Fig. 3:**
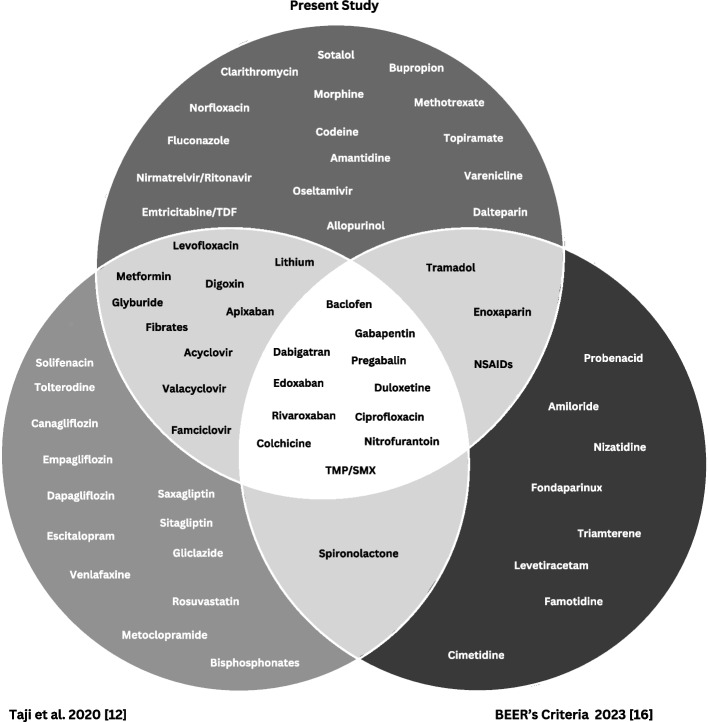
Venn diagram representing the relationship between medications identified in the present study to recent reviews

Eleven of these medications were common in the present study as well as the two previous reviews [Fig. 3]. Compared to the two previous reviews, 16 medications were unique to the present study [12, 16]. The most common comments by panel members in the survey free-text field included dosing must consider indication (prophylaxis versus treatment), patient specific factors, and need for collaboration and engagement with prescriber to support decision making.

## Discussion

We conducted a scoping review followed by a modified Delphi by pharmacists practicing in nephrology, geriatrics and primary care to develop a list of 40 relevant medications for community pharmacists to adjust or avoid in individuals with an eGFR < 30 mL/min with stable CKD. Compared to the two recent reviews, we identified 16 medications unique to the present study including *nirmatrelvir/ritonavir, emtricitabine/tenofovir disoproxil fumarate, varenicline, methotrexate, fluconazole, clarithromycin, norfloxacin, oseltamivir, amantadine, allopurinol, topiramate, dalteparin, sotalol, morphine, codeine and bupropion* [12, 16]. Common medications in the present study and two previous reviews were *gabapentinoids, direct oral acting anticoagulants, baclofen, colchicine, duloxetine, ciprofloxacin, nitrofurantoin, and trimethoprim/ sulfamethoxazole* [12, 16]*.* While bisphosphonates, proton pump inhibitors, and RASI were identified medication classes in our scoping review, we elected not to include these therapies in our modified Delphi. Rather, we plan to provide general guidance on management of these therapies in community pharmacy practice.

In addition to the two reviews included for comparison in the present study, a German study developed a renally relevant drug list (RRD-list) of 16 drug groups from a prospective outpatient nephrology clinic evaluation based on pharmacists review of medications, drug-therapy problems and nephrologists’ recommendations [12, 26] The RRD-list differs from the present study as it included medications used for treatment of CKD-related diseases such as antacids, immunosuppressives, diuretics, renin–angiotensin–aldosterone system inhibitors (RASI) and antihypertensives.

Inappropriate dosing of medications is common in those with kidney disease [5–12]. A review in nonhospital settings identified the prevalence of inappropriate prescribing in individuals with kidney disease to be as high as 60% and to be associated with a high risk for hospitalization and all-cause mortality [27]. A recent prospective study identified that more than 27% of adverse drug reactions (ADRs) are preventable or potentially preventable with eGFR being a major risk factor for serious ADRs [13].

Pharmacists in the community setting are well positioned to support the safe use of medications needed to protect and preserve kidney function in individuals with eGFR < 30 mL/min. Two studies reported the important role community pharmacists play in the detection of nephrotoxic drugs and dose adjustment, and drug related problems in those with CKD [28, 29]. A key component of these studies was pharmacists’ collaboration with other prescribers. This was also a common theme identified from comments in the present study. In recent year, the scope of practice for pharmacists in Canada has expanded, varying by province, to enable pharmacists to prescribe adaptations (e.g., modify a dose or regimen) or prescribe a medication for chronic condition based on patient-specific factors (e.g., age, weight, organ function, medical conditions, adverse drug reactions and others) [30]. However, inconsistencies between medication resources can complicate community pharmacists’ decision making to adjust a medication [15]. Community pharmacists have expressed challenges in suggesting doses given the different renal function formulas and recommendations from resources using different kidney function cut points [15]. For example, most resources recommend avoiding metformin in those with eGFR < 30 mL/min but very limited data also exists to support metformin 500 mg per day in those with eGFR 15–29 mL/min. A qualitative study of interviews of community pharmacists conducted by one team member (J.W.), highlighted the need for an evidence and expert-informed tool to enable community pharmacists to confidently dose adjust harmful medications in those with eGFR < 30 mL/min [15]. The identification of the top medications to adjust or avoid in the present study for community pharmacy practice is an important first step to inform decision support tools to facilitate appropriate dosing in this population.

This study has several strengths. Individuals with advanced kidney disease are often excluded from clinical trials. To ensure retrieval of all relevant reports, our scoping review was not limited to clinical trial reports; instead, we included a wide range of study designs. Secondly, our modified Delphi included a diverse group of expert pharmacists across eight provinces practicing in nephrology, geriatrics and primary care. Their iterative feedback refined and informed the final list of relevant medications for community pharmacists to adjust or avoid in those with eGFR < 30 mL/min. We tailored our medication list to individuals with the highest potential for medication harm (eGFR < 30 mL/min versus eGFR < 60 mL/min), which may be more manageable and actionable by community pharmacists in jurisdictions within or outside Canada. Finally, we used eGFR (mL/min) as the preferred method of estimating kidney function in our modified Delphi as it is widely available, accepted and clinically applicable. There are several potential limitations. In our scoping review, we did not assess publication bias, nor the quality of the different types of studies and methodologies included. Extraction of data in all domains of interest was challenging given the variety of studies assessed. Despite this, we were able to identify key medications requiring dose adjustment or avoidance in eGFR < 30 mL/min. It is possible that our search was not exhaustive as we limited literature published in English and placed limits on years for our bibliographic database searches. Therefore, we may have missed medications removed by the kidneys which require dose adaptations to mitigate harm. To account for this, we allowed Delphi panelist to add medications for consideration for subsequent rounds. Our final list will periodically require ongoing updates as medication information in CKD becomes available, otherwise, it will become outdated. While our combined scoping review and modified Delphi provides a pragmatic list of common and/or higher risk medication requiring dose adjustment or avoidance in individuals with eGFR < 30 mL/min, it does not provide the decision support and collaboration required too effectively and safely do so. Participants noted that context is needed alongside selecting whether to adjust or avoid a medication in this population. While ccommunity pharmacists were not included in this stage of the research, they will be key stakeholders to validate the content and face validity of the algorithms for each of the medications identified in the modified Delphi process, as they bring valuable contextual insights regarding practical applicability. Therefore, the next phase of this research is to develop and validate medication algorithms for eGFR < 30 mL/min for each medication on our list for inclusion in an electronic decision support tool for community pharmacists.

## Conclusion

This study identified a list of the top 40 common and higher risk medications for community pharmacists to adjust or avoid to enhance prescribing safety in individuals with an eGFR < 30 mL/min. This collection of medications includes new and frequently prescribed medication relevant to community pharmacy practice. Future research will focus on creating evidence and export-informed decision support tool of these medications to facilitate community pharmacist in mitigating harm from inappropriate prescribing in this population.

## Supplementary Information

Additional file 1: Search Strategy.

Additional file 2: Drug Dosing Resource for Modified Delphi.

Additionsl file 3: Modified Delphi Survey Rounds 1, 2, 3.

Additional file 4: Medications presented to Modified Delphi.

Additional file 5: Scoping Review Data Extraction of 12 Unique Medications.

Additional file 6: Medications Selected to Dose Adjust by all Participants for Round 1 and 2.

Additional file 7: Top 40 Medication List Selected by Modified Delphi Panel.

## Data Availability

No datasets were generated or analysed during the current study.

## Abbreviations

CKD: Chronic Kidney Disease
eGFR: Estimated glomerular filtration rate
PAIR: Pharmacotherapy assessment in chronic renal disease
PRISMA-ScR: PRISMA Extension for Scoping Reviews
