# Supplementary material for: Medications for community pharmacists to dose adjust or avoid to enhance prescribing safety in individuals with advanced chronic kidney disease: a scoping review and modified Delphi
Source: BMC Nephrol. 2024 Oct 29;25:386. doi: 10.1186/s12882-024-03829-y (PMC11523796; doi:10.1186/s12882-024-03829-y)
Supplement: Supplementary file 1 — Additional file 1: Search Strategy. [file 12882_2024_3829_MOESM1_ESM.pdf]

Additional File 1. Search Strategy

Medline (Ovid). Date of search: March 3, 2024.

No.	Query	Results
1	exp "Drug-Related Side Effects and Adverse Reactions"/ or Drug Monitoring/ or drug monitoring.ti,ab,kf. or pharmacovigilance.ti,ab,kf. or Pharmacovigilance/ or (patient safety/ and (dose or medication? or prescription? or drug?).ti,ab,kf.) or inappropriate prescribing/ or exp Medication Errors/ or Potentially Inappropriate Medication List/ or Deprescriptions/ or contraindications, drug/ or deprescri*.ti,ab,kf. or ((dose or medication? or prescription? or drug?) adj2 (side-effect? or safety or harm* or toxic* or appropriate or inappropriate or discrepanc* or incorrect or high-risk or avoid* or error? or contraindication? or adjust* or cessation* or reduction* or discontinu* or taper* or acute kidney injur* or nephrotoxic* or adverse or pharmacotoxic*)).ti,ab,kf. or (PIM List? or potentially inappropriate medication? or overprescri* or over prescri*).ti,ab,kf. or ((Kidney or renal) and ADR?).ti,ab,kf.	349686
2	exp Renal insufficiency, chronic/ or ((chronic or diabetic or advanced) adj2 (kidney or renal)).ti,ab,kf. or (end stage kidney or end stage renal or endstage kidney or endstage renal).ti,ab,kf. or ckd.ti,ab,kf. or ((kidney or renal) adj1 (disease or insufficiency)).ti,ab,kf.	277817
3	(exp infant/ or exp child/ or adolescent/) not exp adult/	2185885
4	(letter or comment or editorial or newspaper article).pt.	2237339
5	exp animals/ not humans/	5200495
6	1 and 2	6870
7	3 or 4 or 5	9395524
8	6 not 7	6203
9	limit 8 to (english language and yr="2022 -Current")	775

Embase (Elsevier). Date of search: March 3, 2024.

No.	Query	Results
1	'adverse drug reaction'/de OR 'drug fatality'/de OR 'unspecified side effect'/de OR 'pharmacovigilance'/exp OR 'pharmacovigilance':ti,ab,kw OR 'drug monitoring'/de OR 'drug monitoring':ti,ab,kw OR ((dose:ti,ab,kw OR medication$:ti,ab,kw OR prescription$:ti,ab,kw OR drug$:ti,ab,kw) AND 'patient safety'/exp) OR 'prescribing error'/exp OR 'medication error'/exp OR 'deprescription'/exp OR 'drug contraindication'/de OR deprescri*:ti,ab,kw OR (((dose OR medication$ OR prescription$ OR drug$) NEAR/2 ('side-effect$' OR safety OR harm$ OR toxic* OR appropriate OR inappropriate OR discrepanc* OR incorrect OR 'high risk' OR avoid* OR error$ OR contraindication$ OR adjust* OR	789815

	adjust* OR cessation* OR reduction$ OR discontinu* OR taper OR 'acute kidney injur*' OR nephrotoxic* OR adverse OR pharmacotoxic*)):ti,ab,kw) OR 'pim list':ti,ab,kw OR 'potentially inappropriate medication$':ti,ab,kw OR overprescri*:ti,ab,kw OR 'over prescri*':ti,ab,kw OR ((kidney:ti,ab,kw OR renal:ti,ab,kw) AND adr$:ti,ab,kw)	
2	'chronic kidney failure'/exp OR (((chronic OR diabetic OR advanced) NEAR/2 (kidney OR renal)):ti,ab,kw) OR 'end stage kidney':ti,ab,kw OR 'end stage renal':ti,ab,kw OR 'endstage kidney':ti,ab,kw OR 'endstage renal':ti,ab,kw OR ckd:ti,ab,kw OR (((kidney OR renal) NEAR/1 (disease OR insufficiency)):ti,ab,kw)	430698
3	('child'/exp OR 'adolescent'/de) NOT ('adult'/exp NOT 'adult plant'/de)	2794610
4	letter:it OR editorial:it OR note:it	3054911
5	'animal'/exp NOT 'human'/exp	6089658
6	1 AND 2	20444
7	3 OR 4 OR 5	11575305
8	6 NOT 7	18759
9	8 AND [english]/lim AND [2022-2024]/py	3345

CINAHL (Ebsco). Date of search: March 3, 2024.

No.	Query	Results
1	MH “adverse drug event” OR MH “pharmacovigilance” OR MH “drug monitoring” OR TX (“pharmacovigilance” OR “drug monitoring”) OR MH “medication errors+” OR (MH “patient safety” AND TX (“dose” OR “medication#” OR “prescription#” OR “drug#”)) OR OR MH “deprescribing” OR TX (deprescri*) OR TX ((dose OR medication# OR prescription# OR drug#) N2 (“side effect#” OR safety OR harm* OR toxic* OR inappropriate OR discrepanc* OR incorrect OR “high risk” OR avoid* OR error* OR contraindication* OR adjust* OR cessation* OR reduction* OR discontinu* OR 'acute kidney injur*' OR nephrotoxic* OR adverse OR pharmacotoxic*)) OR TX (“pim list*” OR “potentially inappropriate medication#” OR “overprescri*” OR “over prescri*” OR “treatment contraindication*”) OR TX ((kidney OR renal) AND (adr#))	183784
2	MH “renal insufficiency, chronic+” OR ((chronic OR diabetic OR advanced) N2 (kidney OR renal)) OR (("end stage kidney" OR "end stage renal" OR "endstage kidney" OR "endstage renal" OR "ckd")) OR ((kidney OR renal) N1 (disease OR insufficiency))	80508
3	(((MH "Child+") OR (MH "adolescent+") OR (MH "minor")) NOT (MH "adult+"))	584616
4	PT letter OR editorial OR note OR comment OR "newspaper article"	442746
5	(MH “animals+” NOT MH “human”)	93581

6	S1 AND S2	4088
7	S3 OR S4 OR S5	1086884
8	S6 NOT S7	3774
9	Limiters – Publication Date: 20220101-20241231	468
10	Narrow by Language: English	463
