# Supplementary material for: Medications for community pharmacists to dose adjust or avoid to enhance prescribing safety in individuals with advanced chronic kidney disease: a scoping review and modified Delphi
Source: BMC Nephrol. 2024 Oct 29;25:386. doi: 10.1186/s12882-024-03829-y (PMC11523796; doi:10.1186/s12882-024-03829-y)
Supplement: Supplementary file 2 — Additional file 2: Drug Dosing Resource for Modified Delphi. [file 12882_2024_3829_MOESM2_ESM.pdf]

Additional File 2. Drug Dosing Resource for Modified Delphi

**Abbreviations and Acronyms**	
ADA	American Diabetes Association
BID	Twice daily
CI	Contraindicated
CPS	Compendium of Pharmaceuticals and Specialties
CrCl	Creatinine Clearance
eGFR	estimated Glomerular Filtration Rate
ESRD	End Stage Renal Disease
FDA	Food and Drug Administration
H	Hours
KDIGO	Kidney Disease Improving Global Outcomes
L	Litre
Max	Maximum
Mg	Milligram
min	Minute
mL	Millilitres
q	Every
RPT	Renal Pharmacotherapy
SCr	Serum Creatinine
Subcut	Subcutaneous
UFH	Unfractionated Heparin
µmol	Micromoles

Note: For the purpose of this drug resource, kidney function is documented directly from the individual drug resources as either Cockcroft-Gault creatinine clearance (mL/min) or an eGFR (mL/min or mL/min/1.73m^2).

Table of Contents (Hyperlinked)

Antihyperglycemics....3
- Metformin....3
- Glyburide....3
- Saxagliptin....4
- Sitagliptin....4

Lipid Lowering Agents....4
- Bezafibrate....4
- Fenofibrates....5
- Rosuvastatin....5

Antimuscarinic Agents....6
- Solifenacin....6
- Tolterodine....6

Anticonvulsants....7
- Gabapentin....7
- Pregabalin....8
- Topiramate....8
- Cenobamate....9

Urate Lowering Agents....9
- Allopurinol....9
- Colchicine....10
- Febuxostat....10

Anticoagulants....11
- Apixaban (Atrial Fibrillation)....11
- Dabigatran (Atrial Fibrillation)....11
- Edoxaban (Atrial Fibrillation)....12
- Rivaroxaban (Atrial Fibrillation)....12
- Dalteparin (VTE Treatment)....13
- Tinzaparin (VTE Treatment)....13
- Enoxaparin (VTE Prophylaxis and Treatment)....13

Antivirals....14
- Acyclovir....14
- Famciclovir....15
- Valacyclovir....16
- Oseltamivir....17
- Paxlovid (nirmatrelvir/ritonavir)....18
- Truvada (emtricitabine/ tenofovir disoproxil fumarate)....18

Antimicrobials....19
- Amoxicillin....19
- Amoxicillin/Clavulanic Acid....20
- Cephalexin....21
- Clarithromycin....21
- Sulfamethoxazole-Trimethoprim....22
- Ciprofloxacin....23
- Levofloxacin....24
- Norfloxacin....25
- Nitrofurantoin....25

Antifungals....26
- Fluconazole....26

Analgesics/Opioids....27
- NSAIDs....27
- Codeine....28
- Morphine....28
- Tramadol....29

H2RA Antagonists....29
- Ranitidine....29
- Famotidine....30

Antidepressants....31
- Duloxetine....31
- Bupropion....31
- Venlafaxine....32
- Escitalopram....32
- Mirtazapine....32

Mineralocorticoid Receptor Antagonists....33
- Eplerenone....33
- Spironolactone....33

Non-Steroidal Mineralocorticoid Receptor Antagonist....34
- Finerenone....34

CGRP Antagonists....34
- Atogepant....34
- Ubrogepant....34

Other Drugs....35
- Baclofen....35
- Lithium....35
- Metoclopramide....36
- Digoxin....37
- Methotrexate....38
- Tizanidine....39
- Risperidone....39
- Abrocitinib....40
- Varenicline....40
- Sildenafil....40
- Tadalafil....41
- Sotalol....42
- Amantadine....43
- Memantine....43

Antihyperglycemics

Resource	Metformin
CPS	CrCl < 30 mL/min: avoid.
Lexicomp	eGFR < 30 mL/min/1.73 m^2: contraindicated.
Micromedex	eGFR < 30 mL/min/1.73 m^2: contraindicated.
Monographs	Canadian: contraindicated in renal impairment (males with SCr ≥ 136 µmol/L, females with SCr ≥124 µmol/L) or abnormal CrCl <60 mL/min.<br>American: eGFR < 30 mL/min/1.73 m^2: contraindicated.
RPT	FDA-approved product labeling: eGFR < 30 mL/min: contraindicated.<br>Alternative adjustment: GFR 15 - 29 mL/min: 250 mg BID with food, or 500 mg ER once daily (limited data) with close monitoring.<br>GFR ≤ 15 mL/min: Avoid due to metabolic complications (lactic acidosis).
RxFiles	eGFR < 30 mL/min: avoid.<br>Some evidence suggesting that eGFR ≥ 15 mL/min: 500 mg once daily.
Diabetes Canada	eGFR < 30 mL/min/1.73 m^2: use alternative agent.<br>eGFR 15-29 mL/min/1.73 m^2: 500 mg daily (limited data).<br>eGFR < 15 mL/min/1.73 m^2 : avoid
KDIGO	eGFR < 30 mL/min/1.73 m^2: discontinue.
ADA	eGFR < 30 mL/min/1.73 m^2: contraindicated.
KDIGO/ADA Consensus	eGFR < 30 mL/min/1.73 m^2: contraindicated.

Resource	Glyburide
CPS	CrCl < 30 mL/min: avoid.
Lexicomp	eGFR < 60 mL/min/1.73 m^2: avoid.
Micromedex	Dosage in Renal Failure: avoid. If glyburide is required, consider an initial dosage of 1.25 mg/day with a conservative maintenance dosing.
Monographs	Canadian: Contraindicated in renal impairment.<br>American: Both initial and maintenance dosing should be conservative to avoid hypoglycemia.
RPT	GFR < 30 mL/min: avoid.
RxFiles	eGFR < 60 mL/min: contraindicated.
Diabetes Canada	eGFR < 60 mL/min/1.73m^2: use alternative agent.
KIDGO/ADA Consensus	eGFR < 45 mL/min/1.73m^2: use not recommended. Sulfonylureas that are renally cleared or long-acting should be avoided in severe renal impairment.

Resource	Saxagliptin
CPS	eGFR < 30 mL/min/1.73 m^2: 2.5 mg daily.
Lexicomp	eGFR < 45 mL/min/1.73m^2: 2.5 mg daily.
Micromedex	eGFR < 45 mL/min/1.73m^2: 2.5 mg daily.
Monographs	Canadian:<br>eGFR < 45 mL/min/1.73m^2: 2.5 mg once daily.<br>American:<br>eGFR ≤ 50 mL/min/1.73m^2: 2.5 mg daily.
RPT	FDA-approved product labelling: CrCl≤ 50 mL/min: 2.5 mg daily.<br>Alternative adjustment: CrCl ≤ 55 mL/min/1.73m^2: 2.5 – 5 mg daily.
RxFiles	eGFR < 50 mL/min: 2.5 mg daily.<br>eGFR < 15 mL/min: use alternative agent.
Diabetes Canada	eGFR < 45 – 30 mL/min/1.73m^2: 2.5 mg daily.<br>eGFR < 30 mL/min/1.73m^2: use alternative agent.
KDIGO/ADA Consensus	eGFR < 45 mL/min/1.73m^2: maximum 2.5 mg daily.

Resource	Sitagliptin
CPS	eGFR < 30 mL/min/1.73m^2: 25 mg once daily.
Lexicomp	eGFR < 30 mL/min/1.73m^2: 25 mg once daily.
Micromedex	eGFR < 30 mL/min/1.73m^2: 25 mg once daily.
Monographs	Canadian: CrCl < 30 mL/min: 25 mg once daily.<br>American: eGFR < 30 mL/min/1.73m^2: 25 mg once daily
RPT	FDA-approved product labelling: GFR < 30 mL/min/1.73m^2: 25 mg once daily.<br>Alternative adjustment: GFR < 30 mL/min/1.73m2: 12.5 – 25 mg once daily, or 50 mg every 48 hours.
RxFiles	eGFR < 30 mL/min: 25 mg daily.
Diabetes Canada	eGFR < 30 mL/min/1.73m^2: 25 mg once daily.
KDIGO/ADA Consensus	eGFR < 30 mL/min/1.73m^2: 25 mg once daily.

Lipid Lowering Agents

Resource	Bezafibrate
CPS	CrCl < 60 mL/min: avoid.
Lexicomp	CrCl < 60 mL/min or SCr > 1.5 mg/dL (135 µmol/L): contraindicated.
Micromedex	CrCl 40 – 60 mL/min: 400 mg daily.<br>CrCl 15 – 40 mL/min: 200 mg daily or every other day.
Monographs	Canadian: CrCl < 60 mL/min (or SCr < 135 µmol/L): should not be used.<br>American: Not FDA approved.
RPT	Not included in this reference.

Resources	Fenofibrates
CPS	All formulations:<br>CrCl < 20 mL/min: avoid.<br>Use lower initial doses for CrCl 21 – 100 mL/min, adjust according to tolerance and response.
Lexicomp	CrCl > 30 – 80 mL/min: use lowest tablet strength in a formulation that is ≤ 67 mg.<br>CrCl ≤ 30 mL/min: contraindicated.
Micromedex	All formulations:<br>CrCl 30 – 80 mL/min: initiate at lowest available daily dose, may adjust based on renal function and lipid levels.<br>CrCl < 30 mL/min: contraindicated.
Monographs	Canadian:<br>CrCl 20 – 85 mL/min (women) or 20 – 95 mL/min (men): initiated at 67 mg/day (for micronized) or 100 mg/day (for non-micronized) and increased based on tolerance and lipid response.<br>CrCl < 20 mL/min: fenofibrate should not be used.<br>American:<br>CrCl 30 – 59 mL/min/1.73m^2: reduce initial doses, increase only after assessing renal function and lipid concentration.<br>CrCl < 30 mL/min/1.73m^2: avoid.
RPT	FDA-approved product labelling:<br>CrCl 30 – 80 mL/min: 48 mg daily, increasing based on renal function and lipid levels.<br>CrCl < 30 mL/min: avoid.<br>Alternative adjustment:<br>GFR 30 – 59 mL/min: 48 – 145 mg daily.<br>GFR < 30 mL/min: data not available.

Resource	Rosuvastatin
CPS	CrCl < 30 mL/min/1.73m^2: 10 mg daily maximum.
Lexicomp	CrCl < 30 mL/min: 5 to 10 mg daily.
Micromedex	CrCl < 30 mL/min/1.73m^2: 5 mg daily initially, 10 mg daily maximum.
Monographs	Canadian:<br>CrCl < 30 mL/min/1.73m^2: 5 mg daily initially, 10 mg daily maximum.<br>American:<br>CrCl < 30 mL/min: 5 mg daily initially, 10 mg daily maximum.
RPT	FDA-approved product labeling:<br>CrCl < 30 mL/min: 5 mg daily initially, 10 mg daily maximum.<br>Alternative adjustment:<br>GFR < 30 mL/min: 5 mg daily initially, 10 mg daily maximum.

Antimuscarinic Agents

**Resource**	Solifenacin
CPS	CrCl < 30 mL/min: 5 mg daily maximum.
Lexicomp	CrCl < 30 mL/min: 5 mg daily maximum.
Micromedex	CrCl < 30 mL/min: 5 mg daily maximum.
Monographs	Canadian:<br>CrCl < 30 mL/min: 5 mg daily maximum.<br>American:<br>CrCl < 30 mL/min: 5 mg daily.
RPT	FDA-approved product labelling:<br>CrCl < 30 mL/min: 5 mg daily.
RxFiles	CrCl < 30 mL/min: 5 mg daily.

**Resources**	Tolterodine
CPS	Immediate release:<br>GFR < 30 mL/min: 1 mg BID maximum.<br>Extended release:<br>GFR < 30 mL/min: 2 mg daily maximum.
Lexicomp	Immediate release:<br>CrCl 10 – 30 mL/min: 1 mg BID.<br>Extended release:<br>CrCl 10 – 30 mL/min: 2 mg daily.<br>CrCl < 10 mL/min: not recommended.
Micromedex	Immediate release:<br>Renal impairment: 1 mg BID.<br>Extended release:<br>CrCl 10 – 30 mL/min: 2 mg once daily.<br>CrCl < 10 mL/min: not recommended.
Monographs	Canadian:<br>Immediate release: Renal impairment: 1 mg BID maximum.<br>Extended release: Renal impairment: 2 mg daily maximum.<br>American:<br>Immediate release: Renal impairment: 1 mg BID.<br>Extended release: Renal impairment: 2 mg daily.
RPT	FDA-approved product labelling:<br>Immediate release:<br>CrCl 10 – 30 mL/min: 1 mg BID.<br>CrCl < 10 mL/min: not recommended.<br>Extended release:<br>CrCl 10 – 30 mL/min: 2 mg daily.<br>CrCl < 10 mL/min: not recommended.
RxFiles	CrCl 10 – 30 mL/min: 2 mg daily.

Anticonvulsants

Resource	Gabapentin
CPS	CrCl > 15 – 29 mL/min: 200 – 700 mg total daily dose (single daily dose).<br>CrCl < 15 mL/min: 100 – 300 mg daily dose. (single daily dose. Reduce dose in proportion to CrCl. CrCl = 7.5 ml/min will ½ dose compared to a CrCl = 15 mL/min).
Lexicomp	CrCl 15 – 29 mL/min: 600 mg total daily dose (daily or BID).<br>CrCl < 15 mL/min: 300 mg total daily dose. (single daily dose. Reduce dose in proportion to CrCl. CrCl = 7.5 ml/min will ½ dose compared to a CrCl = 15 mL/min).
Micromedex	CrCl 15 – 29 mL/min: 200 – 700 mg total daily dose (single daily dose).<br>CrCl < 15 mL/min: 100 – 300 mg total daily dose. (single daily dose. Reduce dose in proportion to CrCl. CrCl = 7.5 ml/min will ½ dose compared to a CrCl = 15 mL/min).
Monographs	Canadian:<br>CrCl > 15 – 29: 200 – 700 mg total daily dose (single daily dose).<br>CrCl < 15 mL/min: 100 – 300 mg total daily dose. (single daily dose. Reduce dose in proportion to CrCl. CrCl = 7.5 ml/min will ½ dose compared to a CrCl = 15 mL/min).<br>American:<br>CrCl > 15 – 29 mL/min: 200 – 700 mg total daily dose (daily or BID).<br>CrCl < 15 mL/min: 100 – 300 mg total daily dose. (single daily dose. Reduce dose in proportion to CrCl. CrCl = 7.5 ml/min will ½ dose compared to a CrCl = 15 mL/min).
RPT	FDA-approved product labelling:<br>CrCl 15 – 29 mL/min: 200 – 700 mg total daily dose (single daily dose).<br>CrCl < 15 mL/min: 100-300 mg total daily dose (single daily dose).<br>Alternative adjustment:<br>GFR 15 – 29 mL/min: 200-600 mg daily.<br>GFR < 15 mL/min: 100 mg daily.
RxFiles	CrCl 15 – 30 mL/min: 600 mg total daily dose.<br>CrCl < 15 mL/min: 300 mg/day total daily dose.

**Resources**	Pregabalin
CPS	CrCl 15 – 30 mL/min: 25 – 150 mg total daily dose (daily or BID).<br>CrCl < 15 mL/min: 25 – 75 mg total daily dose.
Lexicomp	CrCl 15 – < 30 mL/min: 25 – 150 mg total daily dose (daily or BID).<br>CrCl < 15 mL/min: 25 – 75 mg total daily dose.
Micromedex	CrCl 15 – 30 mL/min: 25 – 150 mg total daily dose (daily or BID).<br>CrCl < 15 mL/min: 25 – 75 mg total daily dose.
Monographs	Canadian:<br>CrCl 15 – 30 mL/min: 150 mg total daily dose (daily or BID).<br>CrCl < 15 mL/min: 75 mg total daily dose.<br>American:<br>CrCl 15 – 30 mL/min: 150 mg total daily dose (daily or BID).<br>CrCl < 15 mL/min: 75 mg total daily dose.
RPT	FDA-approved product labelling:<br>CrCl 15 – 30 mL/min: 150 mg total daily dose (daily or BID).<br>CrCl < 15 mL/min: 75 mg total daily dose.<br>Alternative adjustment:<br>GFR. 15 – 29 mL/min: 25 – 75 mg every 12 to 24 hours (daily or BID).<br>GFR < 15 mL/min: 25 – 75 mg daily.
RxFiles	CrCl 15 – 30 mL/min: 150 mg/day total daily dose.<br>CrCl < 15 mL/min: 75 mg/day total daily dose.

**Resources**	Topiramate
CPS	CrCl < 70 mL/min/1.73 m^2: half the usual adult dose recommended.
Lexicomp	CrCl < 70 mL/min/1.73 m^2: half the usual adult dose recommended; titrate slowly.
Micromedex	CrCl < 70 mL/min/1.73 m^2: half the usual adult dose recommended.
Monographs	Canadian:<br>CrCl < 70 mL/min/1.73 m^2: half the usual adult dose recommended.<br>American:<br>CrCl < 70 mL/min/1.73 m^2: half the usual adult dose recommended.
RPT	FDA – approved labeling:<br>CrCl < 70 mL/min: half the usual recommended dose.<br>Alternative adjustment:<br>GFR 15-29 mL/min: 50 – 100 mg orally every 12 hours.<br>GFR < 15 mL/min: 20 – 50 mg enterally every 12 hours.

Resources	Cenobamate
CPS	CrCl < 30 mL/min: 200 mg maximum.<br>End stage renal disease: avoid.
Lexicomp	CrCl< 90 mL/min: use with caution.<br>End stage renal disease: avoid use.
Micromedex	Mild, moderate or severe (CrCl <30 mL/min) renal impairment: use with caution.
Monographs	Canadian:<br>Mild, moderate, severe (CrCl <30 mL/min) renal impairment: 200 mg maximum.<br>ESRD or dialysis: use not recommended.<br>American:<br>Mild, moderate, or severe (CrCl <30 mL/min) renal impairment: use with caution.<br>ESRD: avoid use.
RPT	Use with caution in renal impairment; use not recommended in ESRD.

Urate Lowering Agents

Resource	Allopurinol
CPS	CrCl < 30 mL/min: initially 50 mg daily, dose titration based on adverse effects and signs of drug toxicity.<br>Some clinicians recommend: CrCl 10 – 20 mL/min: 200 mg daily; CrCl 3 – 10 mL/min: ≤ 100 mg daily; CrCl < 3 mL/min: ≤ 100 mg at extended intervals.
Lexicomp	eGFR > 15 – 30 mL/min/1.73m^2: initially 50 mg every other day.<br>eGFR 5 – 15 mL/min/1.73m^2: initially 50 mg twice weekly.<br>eGFR < 5 mL/min/1.73m^2: initially 50 mg once weekly.
Micromedex	eGFR: 15 – 30 mL/min/1.73 m^2: 50 mg every 2 days.<br>eGFR: 5- 15 mL/min/1.73 m^2: 50 mg twice weekly.<br>eGFR < 5 mL/min/1.73 m^2: 50 mg weekly.
Monographs	Canadian:<br>CrCl 10 – 20 mL/min: 200 mg daily.<br>CrCl < 10 mL/min: 100 mg daily.<br>CrCl < 3 mL/min: dosing intervals may need to be lengthened.<br>American:<br>CrCl 10 – 20 mL/min: 200 mg daily.<br>CrCl < 10 mL/min: ≤ 100 mg daily.<br>CrCl < 3 mL/min: increase dosing interval (such as 300 mg twice weekly).
RPT	FDA-approved product labelling:<br>CrCl > 20 mL/min: 200 – 300 mg daily for mild gout; 400 – 600 mg in divided doses for moderately severe tophaceous gout.<br>CrCl 10 – 20 mL/min: 200 mg daily.<br>CrCl 3 – 10 mL/min: 100 mg daily.<br>CrCl < 3 mL/min: 100 mg daily or at extended intervals.<br>Alternative adjustment:<br>GFR 15 – 59 mL/min: 150 mg daily, up to 150 – 200 mg BID after titration according to serum urate.<br>GFR < 15 mL/min: 100 mg daily or 150 mg every other day, up to 300 mg daily if needed.
RxFiles	CKD, initiate at 50 mg daily, and increase by 50 mg every 2-4 weeks.

Resources	Colchicine
CPS	CrCl < 30 mL/min:<br>Gout flare, treatment: no dose adjustment; should not repeat < 2weeks.<br>Gout flare, prophylaxis, Familial Mediterranean fever: 0.3 mg daily.
Lexicomp	CrCl < 30 mL/min:<br>Gout flare, treatment: consider alternate therapy, or 1.2 mg stat and 0.6 mg after 1 hour. Treatment should not be repeated within 2 weeks.<br>Gout flare, prophylaxis: consider alternate therapy, or 0.3 mg daily, or 0.6 mg every other day.<br>Familial Mediterranean fever: initially 0.3 mg daily, titrate if required with monitoring.
Micromedex	CrCl < 30 mL/min:<br>Gout flare, treatment: no dose adjustment (1.2 mg stat and 0.6 mg after 1 hour), do not repeat within two weeks.<br>Gout flare, prophylaxis: initially 0.3 mg daily.<br>Familial Mediterranean fever: initially 0.3 mg daily.
Monographs	Canadian:<br>CrCl < 30 mL/min: No dose adjustment provided. Contraindicated in serious renal disease.<br>American:<br>CrCl < 30 mL/min:<br>Gout flare, treatment: no dose adjustment; should not be repeated within 2 weeks.<br>Gout flare, prophylaxis: 0.3 mg daily.<br>Familial Mediterranean fever: 0.3 mg daily initially, increases with monitoring.
RPT	FDA-approved product labelling:<br>CrCl < 30 mL/min:<br>Gout flare, treatment: no dose adjustment; do not repeat within 2 weeks.<br>Gout flare, prophylaxis: 0.3 mg daily, titrate dose with monitoring.<br>Familial Mediterranean fever: 0.3 mg daily, titrate dose if required with monitoring.<br>Alternative adjustment:<br>GFR 15 – 59 mL/min: 0.3 – 0.6 mg once or twice daily.<br>GFR < 15 mL/min: preferably avoid; if necessary, use 0.3 mg daily.
RxFiles	If decreased renal function, decrease dose to every other day if using prolonged treatment (prolonged treatment of 0.6 mg daily to twice daily for 3 – 6 months if starting allopurinol).

Resources	Febuxostat
CPS	Insufficient data for severe renal disease.
Lexicomp	CrCl < 30 mL/min: initial 20 – 40 mg once daily.
Micromedex	CrCl 15 –29 mL/min: maximum 40 mg once daily.
Monographs	Canadian:<br>CrCl < 30 mL/min: use not recommended.<br>American:<br>CrCl 15-29 mL/min: maximum 40 mg once daily.
RPT	Caution in severe renal impairment.

Anticoagulants

Resource	Apixaban (Atrial Fibrillation)
CPS	CrCl 25 – 30 mL/min: 5 mg BID or 2.5 mg BID (individuals with at least two of the following: age ≥ 80 years, body weight ≤60 kg or serum creatinine (Scr) ≥133 µmol/L).<br>CrCl 15 – 24 mL/min: insufficient clinical data to make a recommendation<br>CrCl < 15 mL/min: not recommended.
Lexicomp	CrCL 15 – 29: 5 mg BID or "some experts recommend 2.5 mg BID".<br>CrCl 15 – 29 mL/min: "some experts recommend 2.5 mg twice daily".
Micromedex	Age ≥ 80 years, body weight ≤60 kg or Scr ≥133 µmol/L: 2.5 mg BID.
Monographs	Canadian:<br>5 mg BID or 2.5 mg BID (individuals with at least two of the following: age ≥ 80 years, body weight ≤60 kg or serum creatinine (Scr) ≥133 µmol/L).<br>CrCl 15 – 24 mL/min: no recommendation, limited data.<br>CrCl < 15: not recommended.
RPT	FDA-approved product labeling & alternative adjustment:<br>CrCl <15, GFR 15-29 or < 15 ml/min: No dosage adjustment recommended.
RxFiles	CrCl < 30 mL/min: cautious use.<br>CrCl < 15 mL/min: avoid.
Thrombosis Canada	5 mg BID or 2.5 mg BID (in individuals with at least two of the following: age ≥ 80 years, body weight ≤60 kg or serum creatinine ≥133 µmol/L).<br>CrCl 15 – 24 mL/min: limited data, no dosing recommendation from manufacturer.<br>CrCl < 15 mL/min: not recommended.

Resources	Dabigatran (Atrial Fibrillation)
CPS	CrCl < 30 mL/min: contraindicated.
Lexicomp	CrCl 15 – 30 mL/min: 75 mg BID.<br>CrCl < 15 mL/min: avoid.
Micromedex	CrCL 15 – 30 mL/min: 75 mg BID.<br>CrCl < 15 mL/min: no recommendations.
Monographs	Canadian: CrCl < 30 mL/min: contraindicated.<br>American:<br>CrCl 15 – 30 mL/min: 75 mg BID.<br>CrCL < 15 ml/min: no recommendation.
RPT	FDA-approved product labelling:<br>CrCl 15 – 30 mL/min: 75 mg BID<br>CrCL < 15 ml/min: No dosing recommendations can be provided. Data not available.
RxFiles	CrCl < 30 mL/min: contraindicated.
Thrombosis Canada	CrCl: < 30 mL/min: contraindicated.

Resources	Edoxaban (Atrial Fibrillation)
CPS	CrCl 15 – 50 mL/min: 30 mg daily.<br>CrCl < 15 mL/min: not recommended.
Lexicomp	CrCl 15 – 50 mL/min: 30 mg daily.<br>CrCl < 15 mL/min: not recommended.
Micromedex	CrCl 15 – 50 mL/min: 30 mg daily.<br>CrCl < 15 mL/min: not recommended.
Monographs	Canadian:<br>CrCl 15 – 50 mL/min: 30 mg daily.<br>CrCl < 15 mL/min: not recommended.<br>American:<br>CrCl 15 – 50 mL/min: reduce dose to 30 mg daily.<br>CrCl < 15 mL/min: not recommended.
RPT	FDA-approved product labelling:<br>CrCl 15 – 50 mL/min: 30 mg daily.<br>CrCl < 15 mL/min: not recommended due to limited clinical data.<br>Alternative adjustment:<br>GFR 15 – 29 mL/min: 15 mg daily (limited data).<br>GFR < 15 mL/min : data limited to single-dose studies.
RxFiles	CrCl 15 – 50 mL/min: 30 mg daily.<br>CrCl < 15 mL/min: avoid due to increased bleeding risk.
Thrombosis Canada	CrCl 15 – 50 mL/min: 30 mg daily.<br>CrCl < 15 mL/min: edoxaban should not be used.

Resources	Rivaroxaban (Atrial Fibrillation)
CPS	CrCl 15 - 30 mL/min: 15 mg daily.<br>CrCL < 15 mL/min: not recommended.
Lexicomp	CrCl: 15 – 50 mL/min: 15 mg daily.<br>CrCl < 15 mL/min: avoid (excluded from clinical trials).
Micromedex	CrCl < 50 mL/min: 15 mg daily.
Monographs	Canadian:<br>CrCl 15– 30 mL/min: 15 mg daily.<br>CrCl < 15 mL/min: not recommended.<br>American:<br>CrCl ≤ 50 mL/min: reduce dose to 15 mg daily.
RPT	FDA-Approved product labelling:<br>CrCl ≤ 50 mL/min: 15 mg daily.<br>Alternative adjustment:<br>GFR 15 – 29 mL/min: 15 mg daily.<br>GFR < 15 mL/min, not on dialysis: contraindicated.
RxFiles	CrCl 15 – 30 mL/min: cautious use.<br>CrCl < 15 mL/min: avoid.
Thrombosis Canada	CrCl 15 – 49 mL/min: 15 mg daily (use cautiously in those with a CrCl 15 – 29 mL/min).<br>CrCl < 15 mL/min: not recommended.

Resources	Dalteparin (VTE Treatment)
CPS	CrCl < 30 mL/min: dose adjustment not specified. Doses for other indications (except VTE prophylaxis) may need to be reduced. Consider monitoring anti-Xa activity.
Lexicomp	CrCl < 30 mL/min: use not recommended in VTE treatment.
Micromedex	CrCl < 30 mL/min : UFH preferred in VTE treatment.
Monographs	Canadian:<br>Consider dose adjustment in renal failure.<br>American:<br>Monitor anti-Xa levels in CrCl < 30 mL/min.
RPT	FDA-approved Product Labeling<br>CrCl < 30 mL/min: monitor anti-Xa levels and adjust.<br>Alternative Adjustment<br>GFR < 30 mL/min: not recommended.
Thrombosis Canada	CrCl < 30 ml/min: VTE Treatment: therapeutic doses generally avoided.

Resources	Tinzaparin (VTE Treatment)
CPS	CrCl <30 mL/min: dose adjustment not specified. Doses for other indications (except VTE prophylaxis) may need to be reduced. Consider monitoring anti-Xa activity.
Lexicomp	CrCl 20-<30 mL/min: no dose adjustment necessary; increase risk of bleeding after 14 days<br>CrCl <20 mL/min: use of alternative agent should be considered.
Micromedex	CrCl > 20 mL/min: 175 U/kg subcut daily based on total body weight.
Monographs	Canadian:<br>CrCl < 30 mL/min: adjust dosage according to anti-Xa levels.<br>American:<br>Consider alternative agent.
RPT	FDA – approved product labeling:<br>CrCl < 60 mL/min: CI in patient above the age of 90.<br>Alternative adjustment:<br>GFR< 60 mL/min: 175 U/kg subcut q24H.
Thrombosis Canada	Available evidence suggest no accumulation in patients with CrCl < 20 ml/min.

Resources	Enoxaparin (VTE Prophylaxis and Treatment)
CPS	CrCl < 30 mL/min: VTE prophylaxis: 20 – 30 mg subcut. daily. VTE Treatment: 1 mg/kg subcut. daily.
Lexicomp<br>Micromedex<br>Monographs<br>RPT	<table><tr><th></th><th>VTE Prophylaxis (most indications)</th><th>VTE treatment</th></tr><tr><td>CrCl < 30 mL/min</td><td>30 mg subcut. daily</td><td>1mg/kg subcut. daily</td></tr></table>
Thrombosis Canada	CrCl < 30 mL/min : VTE Prophylaxis: 30 mg subcut daily or use unfractionated heparin.<br>VTE Treatment: 1 mg subcut daily or use UFH.

Antivirals

<table>
<tr><th>Resource</th><th>Acyclovir</th></tr>
<tr><td>CPS</td><td>
<table>
<tr><th></th><th>If normal dose is 800 mg q4h:</th><th>If normal dose is 400 mg q12h:</th><th>If normal dose is 200 mg q4h:</th></tr>
<tr><td>CrCl > 10 – 25 mL/min/1.73m^2</td><td>800 mg q8h.</td><td>No dose adjustment.</td><td>200 mg q4h, 5 times daily.</td></tr>
<tr><td>CrCl < 10 mL/min/1.73m^2</td><td>800 mg q12h.</td><td>200 mg q12h.</td><td>200 mg q12h.</td></tr>
</table>
</td></tr>
<tr><td>Lexicomp</td><td>
<table>
<tr><th></th><th>If normal dose is 800 mg, 5x daily:</th><th>If normal dose is 400 mg q12h:</th><th>If normal dose is 200 mg, 5x daily:</th></tr>
<tr><td>CrCl 10 – < 25 mL/min/1.73m^2</td><td>800 mg q8h.</td><td>No dose adjustment or reduce to 200 mg q12h.</td><td>No dose adjustment or reduce to 200 mg q8h.</td></tr>
<tr><td>CrCl < 10 mL/min/1.73m^2</td><td>200 mg q12h, or 400 mg q12h for severe infections.</td><td>200 mg q12h.</td><td>200 mg q12h.</td></tr>
</table>
</td></tr>
<tr><td>Micromedex</td><td>
<table>
<tr><th></th><th>If normal dose is 800 mg q4h:</th><th>If normal dose is 400 mg q12h OR 200 mg q4h:</th></tr>
<tr><td>CrCl 10 – 25 mL/min/1.73m^2</td><td>800 mg q8h.</td><td>No dose adjustment.</td></tr>
<tr><td>CrCl < 10 mL/min/1.73m^2</td><td>800 mg q12h.</td><td>200 mg q12h.</td></tr>
</table>
</td></tr>
<tr><td>Monographs RPT</td><td>
<table>
<tr><th></th><th>If normal dose is 800 mg q4h:</th><th>If normal dose is 400 mg q12h:</th><th>If normal dose is 200 mg q4h:</th></tr>
<tr><td>CrCl 10 – 25 mL/min/1.73m^2</td><td>800 mg q8h.</td><td></td><td></td></tr>
<tr><td>CrCl > 10 mL/min/1.73m^2</td><td>800 mg q8h.</td><td>No dose adjustment.</td><td>No dose adjustment.</td></tr>
<tr><td>CrCl < 10 mL/min/1.73m^2</td><td>800 mg q12h.</td><td>200 mg q12h.</td><td>200 mg q12h.</td></tr>
</table>
</td></tr>
<tr><td>RxFiles</td><td>No specific dose adjustment information.</td></tr>
<tr><td>Firstline</td><td>CrCl 10 – 30 mL/min: 400 mg TID.
CrCl < 10 mL/min: 400 mg BID.</td></tr>
<tr><td>Bugs & Drugs</td><td>CrCl 10 – 50 mL/min: 400 mg TID.
CrCl < 10 mL/min : 400 mg q12h.</td></tr>
<tr><td>Sanford Guide</td><td>
<table>
<tr><th></th><th>If normal dose is 800 mg q4h:</th><th>If normal dose is 400 mg q12h:</th><th>If normal dose is 200 mg q4h:</th></tr>
</table>
</td></tr>
</table>

<table>
<tr><td></td><td>
<table>
<tr><td>CrCl 10 – 25 mL/min</td><td>800 mg q8h.</td><td>No dose adjustment.</td><td>No dose adjustment.</td></tr>
<tr><td>CrCl < 10 mL/min</td><td>800 mg q12h.</td><td>200 mg q12h.</td><td>200 mg q12h.</td></tr>
</table>
</td></tr>
</table>

<table>
<tr><th>Resources</th><th>Famciclovir</th></tr>
<tr><td>CPS</td><td>No renal drug dosing recommendations provided. Active metabolite. Assume ≥75% renal elimination for dosage adjustment.</td></tr>
<tr><td>Lexicomp
Micromedex
American Monograph
RPT</td><td>
<table>
<tr><th></th><th>Herpes zoster</th><th>Recurrent genital herpes, or recurrent herpes labialis</th><th>Suppression/ recurrent genital herpes</th><th>Orolabial/genital herpes in HIV patients,</th></tr>
<tr><td>CrCl 20 – 39 mL/min</td><td>500 mg daily.</td><td>500 mg as a single dose.</td><td>125 mg q12h.</td><td>500 mg daily</td></tr>
<tr><td>CrCl < 20 mL/min</td><td>250 mg daily.</td><td>250 mg as a single dose.</td><td>125 mg daily.</td><td>500 mg daily</td></tr>
</table>
</td></tr>
<tr><td>Monographs</td><td>Canadian:
<table>
<tr><th></th><th>Herpes zoster, or Recurrent orolabial/genital herpes in HIV patients</th><th>Recurrent or suppression genital herpes</th></tr>
<tr><td>CrCl 20 – 39 mL/min/1.73m^2</td><td>500 mg daily.</td><td>125 mg q12h.</td></tr>
<tr><td>CrCl < 20 mL/min/1.73m^2</td><td>250 mg daily.</td><td>125 mg daily.</td></tr>
</table>
</td></tr>
<tr><td>RPT</td><td>Alternative adjustment:
GFR < 30 mL/min: 500 mg daily.</td></tr>
<tr><td>RxFiles</td><td>No specific dose adjustment information.</td></tr>
<tr><td>Firstline</td><td>No renal drug dosing recommendations using this reference.</td></tr>
<tr><td>Bugs and Drugs</td><td>CrCl 10 – 50 mL/min: 500 mg q12-24 hours.
CrCl < 10 mL/min (anuric): 250 mg daily.</td></tr>
<tr><td>Sanford Guide</td><td>CrCl 10 – 50 mL/min: 500 mg q12-24h.
CrCl < 10 mL/min: 250 mg daily.</td></tr>
</table>

<table>
<tr><th>Resources</th><th>Valacyclovir</th></tr>
<tr><td>CPS / Monographs (American & Canadian) / RPT/ Micromedex</td><td>
<table>
<tr><th></th><th>Herpes zoster, initial episode of genital herpes</th><th>Recurrent or suppression genital herpes</th><th>Herpes labialis</th></tr>
<tr><td>CrCl 10 – < 30 mL/min</td><td>1000 mg daily.</td><td>500 mg daily. (q48 h if immunocompetent and ≤9 episodes/y)</td><td>500 mg q12h for 1 day.</td></tr>
<tr><td>CrCl < 10 mL/min</td><td>500 mg daily.</td><td>500 mg daily. (q48 h if immunocompetent and ≤9 episodes/y)</td><td>500 mg as a single dose.</td></tr>
</table>
</td></tr>
<tr><td>Lexicomp</td><td>
<table>
<tr><th></th><th>If normal dose is 500 mg daily:</th><th>If normal dose is 1000 mg daily OR 500 mg q12h:</th><th>If normal dose is 1000 mg q12h OR 1000 mg q8h:</th><th>If normal dose is 2000 mg q12h for 2 doses:</th></tr>
<tr><td>CrCl 10 – 30 mL/min</td><td>500 mg q48h.</td><td>500 mg daily.</td><td>1000 mg daily.</td><td>500 mg q12h for 2 doses.</td></tr>
<tr><td>CrCl < 10 mL/min</td><td>500 mg q48h.</td><td>500 mg daily.</td><td>500 mg daily.</td><td>500 mg as a single dose.</td></tr>
</table>
</td></tr>
<tr><td>RxFiles</td><td>No specific dose adjustment information.</td></tr>
<tr><td>Firstline</td><td>
<table>
<tr><th></th><th>Herpes zoster</th><th>Genital herpes</th><th>Herpes labialis</th></tr>
<tr><td>CrCl 10 – 30 mL/min</td><td>1000 mg daily.</td><td>500 – 1000 mg daily (higher doses required for initial episode or if immunocompromised).</td><td>500 mg q12h for 1 day.</td></tr>
<tr><td>CrCL < 10 mL/min:</td><td>500 mg daily.</td><td>500 mg daily.</td><td>500 mg as a single dose.</td></tr>
</table>
</td></tr>
<tr><td>Bugs and Drugs</td><td>CrCl 10 – 50 mL/min: 1000 mg q12-24 hours.
CrCl < 10 mL/min (anuric): 500 mg daily.</td></tr>
<tr><td>Sanford Guide</td><td>Reference dosing: 1000 mg q8h.
CrCl 10 – 50 mL/min: 1000 mg q12-24h.
CrCl < 10 mL/min: 500 mg q24h.</td></tr>
</table>

<table>
<tr><th>Resources</th><th>Oseltamivir</th></tr>
<tr><td>CPS Monographs (Canadian & American)</td><td>
<table>
<tr><th></th><th>Influenza treatment</th><th>Influenza prevention</th></tr>
<tr><td>CrCl 10 – 30 mL/min</td><td>30 mg once daily for 5 days.</td><td>30 mg every other day for 10-14 days.</td></tr>
</table>
</td></tr>
<tr><td>Lexicomp</td><td>
<table>
<tr><th></th><th>Influenza treatment</th><th>Influenza prevention</th></tr>
<tr><td>CrCl > 10 – 30 mL/min</td><td>30 mg daily.</td><td>30 mg q48h.</td></tr>
<tr><td>CrCl ≤ 10 mL/min</td><td>30 mg q48h.</td><td>30 mg once weekly.</td></tr>
</table>
</td></tr>
<tr><td>Micromedex
Sanford Guide</td><td>
<table>
<tr><th></th><th>Influenza treatment</th><th>Influenza prevention</th></tr>
<tr><td>CrCl > 10 – 30 mL/min</td><td>30 mg daily for 5 days.</td><td>30 mg every other day.</td></tr>
<tr><td>CrCl ≤ 10 mL/min not on dialysis</td><td>Not recommended.</td><td>Not recommended.</td></tr>
</table>
</td></tr>
<tr><td>Monographs</td><td>American specific:
CrCl < 10 mL/min: not recommended</td></tr>
<tr><td>RPT</td><td>FDA-Approved product labelling:
<table>
<tr><th></th><th>Influenza treatment</th><th>Influenza prevention</th></tr>
<tr><td>CrCl 11 – 30 mL/min</td><td>30 mg daily for 5 days.</td><td>30 mg every other day.</td></tr>
</table>
</td></tr>
<tr><td>RxFiles</td><td>
<table>
<tr><th></th><th>Influenza treatment</th><th>Influenza prevention</th></tr>
<tr><td>CrCl 10- 30 mL/min</td><td>30 mg daily for 5 days</td><td>30 mg every other day for 7-10 days for up to 1 week after end of outbreak</td></tr>
</table>
</td></tr>
<tr><td>Firstline Bugs and Drugs</td><td>
<table>
<tr><th></th><th>Influenza treatment</th><th>Influenza prevention</th></tr>
<tr><td>CrCl 10-30 mL/min</td><td>30 mg daily</td><td>30 mg every 48 hours for duration of outbreak</td></tr>
<tr><td>CrCl <10 mL/min</td><td>75 mg as a single dose</td><td>Minimal data, consider 30 mg as a single dose and weekly*</td></tr>
</table>
</td></tr>
</table>

Resources	Paxlovid (nirmatrelvir/ritonavir)
CPS	eGFR < 30 mL/min: not recommended.
Lexicomp	eGFR < 30 mL/min: use not recommended by manufacturer; toxicity risk minimal with a 5 day course of treatment (Nirmatrelvir 300 mg and ritonavir 100 mg once on day 1, then 150 mg nirmatrelvir and 100 mg ritonavir once daily for 4 more days).
Micromedex	eGFR < 30 mL/min: use not recommended.
Monographs	Canadian:<br>eGFR < 30 mL/min: use not recommended.<br>American:<br>eGFR <30 mL/min : use not recommended.
RPT	No dosage recommendations from this resource.
Firstline	GFR< 30 mL/min:<br>Day 1: Nirmatrelvir 300 mg and ritonavir 100 mg once.<br>Days 2-5: Nirmatrelvir 150 mg and ritonavir 100 mg once daily for 4 days.

Resources	Truvada (emtricitabine/ tenofovir disoproxil fumarate)
CPS	Pre-Exposure Prophylaxis: not recommended with CrCl < 60 mL/min.<br>Treatment of HIV: Not recommended with CrCl < 30 mL/min.
Lexicomp<br>Micromedex<br>Monographs (Canadian & American)	Treatment of HIV: CrCl < 30 mL/min: avoid<br>Pre-Exposure Prophylaxis: CrCl < 60 mL/min: avoid
RPT	FDA – approved product labeling:<br>CrCl < 30 mL/min: avoid.

Antimicrobials

<table>
<tr><th>Resource</th><th>Amoxicillin</th></tr>
<tr><td>CPS</td><td>CrCl 10 - 50 mL/min: every 8-12 hours
CrCl < 10 mL/min: every 24 hours</td></tr>
<tr><td>Lexicomp</td><td>
<table>
<tr><th>GFR (mL/min)</th><th>If normal dose if 250 – 500 mg every 8 hours</th><th>If normal dose is 875 mg to 1 g every 12 hours</th><th>If normal dose is 1 g every 8 hours</th></tr>
<tr><td>10 – 30</td><td>250 - 500 mg q12h</td><td>500 mg q12h</td><td>1 g q12h</td></tr>
<tr><td>< 10</td><td>250 – 500 mg q12-24h</td><td>500 mg q12-24h</td><td>500 mg q12h</td></tr>
</table>
</td></tr>
<tr><td>Micromedex</td><td>GFR 10-30 mL/min: do not give 875 mg dose; 250 – 500 mg ever 12 hours depending on severity of infection
GFR < 10 mL/min: 250 – 500 mg every 24 hours depending on severity of infection</td></tr>
<tr><td>Monographs</td><td>Canadian:
Reduce dosage proportional to reduced renal function
American:
GFR < 30 mL/min: should not receive 875 mg dose
GFR 10 – 30 mL/min: should receive 500 mg or 250 mg q12h
GFR < 10 mL/min: 500 mg or 250 mg q24h</td></tr>
<tr><td>RPT</td><td>FDA-approved product labeling:
GFR 10 – 30 mL/min: 250 – 500 mg orally or enterally every 12 hours; do not give 875 mg dose
GFR < 10 mL/min: 250 – 500 mg orally or enterally every 24 hours depending on the severity of infection
Alternative Adjustment:
GFR 15-50 mL/min: 250 – 500 mg enterally every 12 hours
GFR < 15 mL/min: 250 – 500 mg enterally every 24 hours</td></tr>
<tr><td>Firstline</td><td>CrCl 10 – 30 mL/min: 500 mg po q12h
CrCl < 10 mL/min: 500 mg po q24h</td></tr>
<tr><td>Bugs & Drugs</td><td>CrCl 10 – 50 mL/min: 500 mg po q8 – 12 h
CrCl < 10 mL/min: 500 mg po q 12 – 24h</td></tr>
</table>

**Resource**	Amoxicillin/Clavulanic Acid
CPS	CrCl 10 – 30 mL/min: 500/125 mg BID<br>CrCl < 10 mL/min: 500/125 once daily<br>Should not give 7:1 ratio to patient with CrCl < 30 mL/min
Lexicomp	CrCl 10 – < 30 mL/min: 250 to 500 mg q12h<br>CrCl <10 mL/min: 250 to 500 mg every 12 to 24 hours
Micromedex	Dose reduction or extension of dosing interval is necessary
Monographs	Canadian:<br>Should not give 7:1 ratio to patients with CrCl < 30 mL/min.<br>CrCl 10 – 30 mL/min: 500/125 BID<br>CrCl < 10 mL/min: 500/125 once daily<br>American:<br>Periodic assessment of renal function is recommended.
RPT	FDA-approved product labeling:<br>GFR 10 – 30 mL/min: 250 – 500 mg orally q12h<br>GFR < 10 mL/min: 250 – 500 mg orally q24h<br>Do not give 875 mg dose<br>Alternative adjustment:<br>GFR 15 – 50 mL/min: 250 – 500 mg enterally q12h<br>GFR < 15 mL/min: 250 – 500 mg enterally q24h
Firstline	CrCl 10 – 30 mL/min: 500 mg po q12h<br>CrCl < 10 mL/min: 500 mg po q24h
Bugs & Drugs	CrCl 10 – 30 mL/min: 500 mg po q12h<br>CrCl < 10 mL/min: 500 mg po q24h<br>Do not use 875 mg tablet if CrCl <30 mL/min

**Resource**	Cephalexin
CPS	CrCl 10 – 50 mL/min: dosing interval of q8 – 12h<br>CrCl< 10 mL/min: dosing interval q12 – 24h
Lexicomp	CrCl 15 - <30 mL/min: 250 – 500 mg q8 – 12h<br>CrCl < 15 mL/min: 250 – 500 mg q12 – 24h
Micromedex	CrCl 15 – <30 mL/min: 250 mg q8 – 12h<br>CrCl 5 – 14 mL/min: 250 mg q24h<br>CrCl 1 – 4 mL/min (not on dialysis): 250 mg q48 – 60 h
Monographs	Canadian:<br>Administer with caution in impaired renal function<br>American:<br>Use with caution in impaired renal function
RPT	FDA-approved product labeling:<br>CrCl 15 - <30 mL/min: 250 mg q8 – 12 h<br>CrCl 5 – 14 mL/min: 250 mg q24h<br>CrCl 1 – 4 mL/min: 250 mg q48 – 60h<br>Alternative adjustment:<br>GFR 15 – 59 mL/min: 250 – 500 mg q6 – 8h<br>GFR < 15 mL/min: 250 – 500 mg q12h
Firstline	CrCl 10 – 30 mL/min: 500 mg po q8 – 12h<br>CrCl < 10 mL/min: 500 mg po q12 – 24h
Bugs & Drugs	CrCl 10 – 50 mL/min: 500 mg q8 – 12h<br>CrCl < 10 mL/min: 500 mg q12 – 24h

**Resource**	Clarithromycin
CPS	CrCl < 30 mL/min: reduce dose by half<br>Dosage should not be continued beyond 14 days
Lexicomp	CrCl < 30 mL/min: reduce total daily dose by half
Micromedex	CrCl < 30 mL/min: reduce dose by half
Monographs	Canadian:<br>CrCl < 30 mL/min: reduce total daily dose by half<br>American:<br>CrCl < 30 mL/min: reduce dose by half
RPT	FDA-approved product labeling:<br>CrCl< 30 mL/min: reduce dosage by 50 %<br>Alternative Adjustment:<br>GFR <60 mL/min: 125 – 500 mg orally q12h
Firstline	CrCl< 30 mL/min: 500 mg po q24h
Bugs & Drugs	CrCl 10 – 50 mL/min: 250 – 500 mg po q 12 – 24h<br>CrCl < 10 mL/min: 250 – 500 mg po q24h

<table>
<tr><th>Resource</th><th>Sulfamethoxazole-Trimethoprim</th></tr>
<tr><td>CPS</td><td>CrCl 15 – 30 mL/min: Either (1) half normal dosing or (2) double dosing interval.
CrCl < 15 mL/min: Not recommended. If required, sulfamethoxazole-trimethoprim 800/160 mg once, then sulfamethoxazole-trimethoprim 400/80 mg daily.</td></tr>
<tr><td>Lexicomp</td><td>CrCl 15 – 30 mL/min: reduce dose to ~50% of normal dose.
CrCl < 15 mL/min: reduce dose to ~25 – 50% of normal dose.</td></tr>
<tr><td>Micromedex</td><td>CrCl 15 – 30 mL/min: decrease dose by 50%.
CrCl < 15 mL/min: not recommended.</td></tr>
<tr><td>Monographs</td><td>Canadian:
CrCl 15 – 30 mL/min: one-half the usual regimen.
CrCl < 15 mL/min: not recommended.
American:
CrCl 15 – 30 mL/min: one-half the usual regimen.
CrCl < 15 mL/min: not recommended.</td></tr>
<tr><td>RPT</td><td>FDA-approved product labeling:
CrCl 15 – 30 mL/min: one-half the usual regimen.
CrCl < 15 mL/min: not recommended.
Alternative adjustment:
GFR 15 – 59 mL/min: 800/160 mg once, then 400/80 mg q12h.
GFR < 15 mL/min: avoid; if no suitable alternative, 800/160 mg once, followed by 400/80 mg daily.</td></tr>
<tr><td>Firstline</td><td>
<table>
<tr><th>Indication</th><th>Skin and urinary infection</th><th>Pneumocystis jirovecii infection</th><th>Stenotrophomonas infection</th></tr>
<tr><td>CrCl 10 – 30 mL/min:</td><td>800/160 mg daily.</td><td>800/160 mg TID – QID.</td><td>800/160 mg TID.</td></tr>
<tr><td>CrCl < 10 mL/min (not on dialysis)</td><td>Not recommended.</td><td>Not recommended, if used: 5 mg/kg PO q24h.</td><td>Not recommended, if used, 5 mg/kg PO q24h.</td></tr>
</table>
</td></tr>
<tr><td>Bugs & Drugs</td><td>CrCl 15 – 25 mL/min: 50% dose reduction.
CrCl < 15 mL/min: do not use.</td></tr>
<tr><td>Sanford Guide</td><td>No oral dosing information available.</td></tr>
</table>

**Resources**	Ciprofloxacin
CPS	CrCl ≤ 30 mL/min: 500 mg daily.
Lexicomp	CrCl < 30 mL/min: 500 mg daily.
Micromedex	CrCl 5 – 29 mL/min: 250 – 500 mg q18h.
Monographs	Canadian:<br>CrCl ≤ 30 mL/min/1.73m^2: 500 mg daily.<br>American:<br>CrCl 5 – 29 mL/min: 250 – 500 mg q18h.
RPT	FDA-approved product labelling:<br>CrCl 5 – 29 mL/min: 250 – 500 mg q18h.<br>Alternative adjustment:<br>GFR 15 – 59 mL/min: 250 – 500 mg q12h.<br>GFR < 15 mL/min or hemodialysis: 250 mg q12h
Firstline	CrCl < 30 mL/min: 500 – 750 mg daily
Bugs and Drugs	CrCl < 30 mL/min: 250 – 750 mg daily.
Sanford Guide	CrCl 5 – 29 mL/min: 500 – 750 mg q24h.

**Resources**	Levofloxacin

CPS

	If normal dose is 750 mg daily:	If normal dose is 500 mg daily:	If normal dose is 250 mg daily:
CrCl 20 – 49 mL/min	750 mg q48h.	500 mg once, then 250 mg daily.	No dose adjustment.
CrCl 10 – 19 mL/min	750 mg once, then 500 mg q48h.	500 mg once, then 250 mg q48h.	250 mg q48h.
CrCl < 10 mL/min	500 mg once, then 500 mg q48h.	500 mg once, then 250 mg q48h.	250 mg q48h.

Lexicomp Micromedex

	If normal dose is 750 mg daily:	If normal dose is 500 mg daily:	If normal dose is 250 mg daily:
CrCl 20 – < 50 mL/min	750 mg q48h.	500 mg once, then 250 mg qh q24h	No dose adjustment.
CrCl < 20 mL/min	750 mg once, then 500 mg q48h.	500 mg once, then 250 mg q48h.	250 mg q48h (except for uncomplicated UTI, where there is no dose adjustment).

Monographs RPT

Canadian and American:

	If normal dose is 750 mg daily:	If normal dose is 500 mg daily:	If normal dosing is 250 mg daily:
CrCl 20 – 49 mL/min	750 mg q48h.	500 mg once, then 250 mg daily.	No dose adjustment.
CrCl 10 – 19 mL/min	750 mg once, then 500 mg q48h.	500 mg once, then 250mg q48h.	250 mg q48h.

RPT

Alternative adjustment:
GFR < 30 mL/min: 500 mg every 24 – 48 hours.

Firstline

Levofloxacin
CrCl 20 – 50 mL/min: 750 mg q48h.
CrCl < 20 mL/min, hemodialysis, or peritoneal dialysis: 750 mg once, then 500 mg q48h.

Bugs & Drugs

Levofloxacin

	If normal dose is 750 mg daily:	If normal dose is 500 mg daily:
CrCl 10 – 50 mL/min	750 mg q48h.	500 mg once, then 250 mg daily.
CrCl < 10 mL/min (anuric)	750mg once, then 500 mg q48h.	500 mg once, then 250 mg q48h.

Sanford Guide

CrCl 20 – 49 mL/min: 750 mg q48h.
CrCl < 20 mL/min: 750 mg once, then 500 mg q48h.

Resources	Norfloxacin
CPS	CrCl < 30 mL/min: 400 mg every 24 hours
Lexicomp	eGFR < 30 mL/min/1.73 m^2: 400 mg once daily
Micromedex	CrCl < 30 mL/min/1.73m^2: 400 mg once daily for the usual duration for UTI treatment<br>CrCl< 10 mL/min: 50% reduction in dose or doubling of interval is recommended
Monographs	Canadian and American:<br>GFR < 30mL/min/1.73m^2: 400 mg once daily
RPT	FDA Approved Product Labeling:<br>CrCl < 30 mL/min: 400 mg once daily
Bugs and Drugs	CrCl 10 – 50 mL/min: 400 mg every 12-24 hours<br>CrCl <10 mL/min: 400 mg every 24 hours

Resource	Nitrofurantoin
CPS	CrCl < 30 mL/min: contraindicated.
Lexicomp	CrCl 30 – 60 mL/min: limited data suggests safe and effective.<br>CrCl < 30 mL/min: avoid.
Micromedex	CrCl 30 – 60 mL/min: study data suggests short term use safe and clinical efficacy similar to those with relatively high eGFR.<br>CrCl < 30 mL/min: avoid.
Monographs	Canadian and American:<br>CrCl < 60 mL/min: contraindicated.
RPT	FDA-approved product labeling:<br>CrCl < 60 mL/min: contraindicated.<br>Alternative adjustment:<br>GFR 10 – 59 mL/min: potentially ineffective with increased risk of pulmonary toxicity; avoid.<br>GFR < 15 mL/min: ineffective; avoid.
Firstline	CrCl > 30 mL/min: no renal adjustment required.<br>CrCl < 30 mL/min: avoid.
Bugs & Drugs	CrCl < 40 – 60 mL/min: contraindicated.
Sanford Guide	CrCl > 30 mL/min: study data suggests can be used with relative safety and efficacy.<br>ESRD: not effective.

Antifungals

**Resources**	Fluconazole
CPS	No specific dosing recommendations from this resource
Lexicomp	CrCl < 50 mL/min: reduce dose by 50%
Micromedex	CrCl < 50 mL/min: reduce dose by 50%<br>CrCl 20 – 50 mL/min: usual dose may be given every 48 hours<br>CrCl < 20 mL/min: 50% of usual dose given every 48 hours<br>CrCl < 50 mL/min in patients with a UTI: oral loading dose of 200 mg, then 4 doses of 50 mg
Monographs	Canadian:<br>CrCl 21 – 50 mL./min: 50% of recommended dose<br>CrCl 11 – 20 mL/min: 25% of recommended dose<br>American:<br>CrCl < 50 mL/min: give 50% of usual dosing
RPT	FDA -Approved Product Labeling:<br>CrCl < 50 mL/min: 50 % of recommended dose<br>Alternative Dosing:<br>GFR 15-59 mL/min: 50-200 mg every 24 hours (50 % dose decrease)<br>GFR < 15 mL/min: 25 – 100 mg every 24 hours (75% dose decrease)
Firstline	CrCl 30 – 50 mL/min: 200- 400 mg po every 24 hours<br>CrCl < 30 mL/min: 200 mg po every 24 hours
Bugs and Drugs	CrCl 10 – 50 mL/min 400 mg po every 24 – 48 hours<br>CrCl<10 mL/min 400 mg po every 48 –72 hours
Sanford guide	CrCl < 50 mL/min : 50 – 300 mg every 24 hours

Analgesics/Opioids

**Resource**	NSAIDs
CPS	CrCl < 30 mL/min: contraindicated.
Lexicomp	CrCl ≤ 30 mL/min: avoid.
Micromedex	Ibuprofen:<br>Advanced renal disease: not recommended.<br>Naproxen:<br>CrCl < 30 mL/min: not recommended.<br>Meloxicam<br>CrCl > 15 mL/min: no adjustment necessary.<br>CrCl < 15 mL/min: not recommended.
Monographs	Canadian:<br>CrCl < 30 mL/min: contraindicated.<br>American:<br>Ibuprofen:<br>Advanced renal disease: avoid.<br>Naproxen:<br>CrCl < 30 mL/min: not recommended.<br>Meloxicam:<br>Severe renal impairment: not recommended.
RPT	FDA-approved product labeling:<br>Ibuprofen:<br>Advanced renal disease: not recommended.<br>Naproxen:<br>CrCl < 30 mL/min: not recommended.<br>Meloxicam:<br>Advanced renal disease: avoid unless benefits outweigh risk; monitor for worsening renal function.

Resource	Codeine
CPS	Renal impairment: dose reduction recommended.
Lexicomp	eGFR < 30 mL/min/1.73m^2: avoid.
Micromedex	GFR 10 – 50 mL/min: 75% of normal dose.<br>GFR < 10 mL/min: 50% of normal dose.
Monographs	Canadian:<br>CrCl 10 – 50 mL/min: 25% dose reduction and titrate as needed.<br>CrCl < 10 mL/min: 50% dose reduction and titrate as needed.<br>American:<br>Severe renal impairment: cautious use.
RPT	FDA-approved product labeling:<br>Renal failure: lower than normal dose or longer dosing intervals due to altered pharmacokinetics and decreased clearance; increased monitoring.<br>Alternative adjustment:<br>GFR 15 – 59 mL/min: 10 – 45 mg q4h prn.<br>GFR < 15 mL/min: avoid unless no suitable alternative; if necessary, 10 – 15 mg initiation with close monitoring.

Resource	Morphine
CPS	Renal impairment: cautious administration.
Lexicomp	CrCl 15 – < 30 mL/min: avoid; if necessary, initiate at 25 – 50% of usual dose.<br>CrCl < 15 mL/min: avoid.
Micromedex	GFR 10 – 50 mL/min: reduce dose by 25%.<br>GFR < 10 mL/min: reduce dose by 50% or consider alternative.
Monographs	Canadian:<br>Renal impairment: reduce dose; cautious use.<br>American:<br>Severe renal impairment: reduce dose; cautious use.
RPT	FDA-approved product labeling:<br>Kidney disease: start patients at a lower than usual dosage; titrate slowly and monitor for signs of respiratory depression, sedation, and hypotension.<br>Alternative adjustment:<br>GFR 15 – 59 mL/min: 75% of usual dose.<br>GFR < 15 mL/min: preferably avoid; if used, 50% of usual dose.

Resource	Tramadol
CPS	Severe Renal impairment: Contraindicated
Lexicomp	CrCl <30 mL/min: increase dosing interval to every 12 hours; maximum 200 mg/day (avoid extended release formulations).
Micromedex	CrCl <30 mL/min: increase dosing interval to every 12 hours; maximum 200 mg/day (avoid extended release formulations).<br>CrCl < 10mL/min: 50 mg q12h
Monographs	Canadian:<br>CrCl <30 mL/min: contraindicated.<br>American:<br>CrCl <30 mL/min: dose reduction is recommended; increase interval to 12 hours with maximum daily dose of 200 mg.
RPT	FDA-approved product labeling:<br>CrCl <30mL/min: increase dosing interval to ever 12 hours, maximum daily dose of 200 mg/day.<br>Alternative Adjustment:<br>GFR 15-59 mL/min: 50 – 100 mg orally every 6-12 hours as needed for pain.<br>GFR <15 mL/min and hemodialysis: 50 mg every 12 hours as needed for pain.

H2RA Antagonists

Resource	Ranitidine
CPS	CrCl 10 – 50 mL/min: 150 mg/day maximum.<br>CrCl < 10 mL/min: 75 – 150 mg daily.
Lexicomp	CrCl < 50 mL/min: Decrease dose by 50%; 150 mg daily is recommended, adjust dose cautiously if necessary.
Micromedex	CrCl < 30 mL/min: 75 mg BID.
Monographs	Canadian:<br>CrCl < 50 mL/min: 150 mg daily.<br>American:<br>CrCl < 50 mL/min: 150 mg daily (may cautiously increase to q12h or more frequently).
RPT	FDA-approved product labeling:<br>CrCl < 50 mL/min: 150 mg daily.<br>Alternative adjustment:<br>CrCl ≤ 59 mL/min: 75 mg BID or 150 mg daily.

<table>
<tr><th>Resource</th><th>Famotidine</th></tr>
<tr><td>CPS</td><td>CrCl 10 – 50 mL/min: reduce to half the dose or increase dosing interval to q36-48h.
CrCl < 10 mL/min: increase dosing interval to q36-48h.</td></tr>
<tr><td>Lexicomp</td><td>
<table>
<tr><th></th><th colspan="3">Reference Dose</th></tr>
<tr><th></th><th>10 mg BID</th><th>20 mg daily</th><th>20 mg BID</th></tr>
<tr><td>CrCl < 30 mL/min</td><td>10 mg every other day</td><td>10 mg every other day</td><td>10 mg daily or 20 mg every other day</td></tr>
</table>
</td></tr>
<tr><td>Micromedex American monograph</td><td>
<table>
<tr><th></th><th>Prevention of recurrent duodenal ulcer</th><th>Active duodenal or gastric ulcer</th><th>Symptomatic non-erosive GERD</th><th>Erosive esophagitis</th></tr>
<tr><td>CrCl < 30 mL/min</td><td>10 mg every other day</td><td>20 mg every other day or 10 mg daily</td><td>20 mg every other day or 10 mg daily</td><td>20 mg every other day or 10 mg daily (if usual dose of 20 mg BID) or 20 mg daily (if usual dose of 40 mg BID)</td></tr>
</table>
CrCl < 60 mL/min (pathologic hypersecretory conditions): avoid use.
</td></tr>
<tr><td>Monographs</td><td>Canadian:
CrCl < 50 mL/min: reduce to half the dose or increase dosing interval to 36 to 48 hours.</td></tr>
<tr><td>RPT</td><td>FDA-approved product labeling:
CrCl < 50 mL/min: 20 mg daily at bedtime or 40 mg q36-48h.
Alternative adjustment:
GFR 15 – 59 mL/min: 10 – 20 mg daily at bedtime.
GFR < 15 mL/min: 5 – 10 mg daily at bedtime.</td></tr>
</table>

Antidepressants

Resource	Duloxetine
CPS	CrCl < 30 mL/min: contraindicated.
Lexicomp	CrCl < 30 mL/min: avoid.<br>Some experts recommend cautious initiation of 30 mg daily, titrating slowly and not exceeding 60 mg daily.
Micromedex	GFR < 30 mL/min: avoid.
Monographs	Canadian:<br>CrCl < 30 mL/min: contraindicated.<br>American:<br>CrCl < 30 mL/min: do not administer.
RPT	FDA-approved product labelling:<br>CrCl < 30 mL/min: should not be used.<br>Alternative adjustment:<br>GFR < 30 mL/min: data not available.
RxFiles	CrCl < 30 mL/min: avoid.

Resource	Bupropion
CPS	Use with caution; reduce dose or increase interval
Lexicomp	CrCl 15 – 60 mL/min: use with caution; consider a maximum daily dose of 150 mg/day<br>CrCl < 15 mL/min: use of alternative is preferred; initiate therapy at 100 mg q48h or 150 mg q72h, titrate gradually to 150 mg/day
Micromedex	GFR < 90 mL/min: consider reducing dose or increasing interval
Monographs	Canadian:<br>Risk of accumulation, reduce dosage or increase interval<br>American:<br>Consider reducing dose.
RPT	Caution in severe renal impairment

Resources	Venlafaxine
CPS	GFR 10 – 70 mL/min: dose decrease by 25 – 50%.
Lexicomp	CrCl < 30 mL/min: 37.5 mg daily initially, not to exceed 50% of the maximum recommended dose (manufacturer lists a maximum recommended dose of 112.5 mg/day).
Micromedex	CrCl < 30 mL/min: total daily dose decrease by 50% or more.
Monographs	Canadian:<br>GFR 10 – 70 mL/min: total daily dose decrease by 25 – 50%.<br>American:<br>CrCl < 30 mL/min: 50% total daily dose reduction.
RPT	FDA-approved product labelling:<br>10 – 70 mL/min: 112.5 – 150 mg/day divided BID or TID (25% decrease).<br>Alternative adjustment:<br>GFR 15 – 59 mL/min, GFR < 15 mL/min, or hemodialysis: 37.5 – 187.5 mg (ER) daily (50% decrease).
RxFiles	Requires renal dose adjustment.

Resource	Escitalopram
CPS	CrCl < 30 mL/min: cautious use.
Lexicomp	CrCl ≥ 20 mL/min: no dose adjustment.<br>CrCl < 20 mL/min: 5 mg initially, gradually titrate with close monitoring.
Micromedex	CrCl < 20 mL/min: specific recommendations not available.
Monographs	Canadian:<br>CrCl < 30 mL/min: cautious use.<br>American:<br>Severe renal impairment: cautious use.
RPT	Caution in severe renal impairment.

Resources	Mirtazapine
CPS	Exercise caution in impaired renal function.
Lexicomp	eGFR < 30 mL/min/1.73m^2: initial 7.5 mg to 15 mg once daily, titrate slowly.
Micromedex	Decrease in dosage may be necessary.
Monographs	Canadian:<br>Monitor dose titration.<br>American:<br>Decreased dosage may be necessary.
RPT	Caution, consider dose reduction in renal impairment; clearance is decreased 50% if CrCl < 10 mL/min.

Mineralocorticoid Receptor Antagonists

**Resources**	Eplerenone
CPS	No dosing information from this resource.
Lexicomp	Heart Failure: eGFR < 30 mL/min: not recommended.<br>Hypertension: CrCl< 50 mL/min or serum creatinine > 2 mg/dL[178 umol/L] (males) or > 1.8 mg/dL [160 umol/L](females): contraindicated.
Micromedex	CrCl < 30 mL/min: contraindicated.<br>CrCl < 50 mL/min in hypertension: contraindicated.
Monographs	Canadian:<br>eGFR < 30 mL/min/1.73 m^2 : contraindicated.<br>eGFR < 50 mL/min/1.73m^2: contraindicated for hypertension treatment.<br>American:<br>CrCl < 30 mL/min : contraindicated.<br>CrCl <50 mL/min : contraindicated for hypertension treatment.
RPT	No dosing information in this resource.

**Resources**	Spironolactone
CPS	No dosage recommendations on this resource.
Lexicomp	eGFR:<30 mL/min/1.73m^2: use not recommended.
Micromedex	No dosage recommendations in this resource for severe renal impairment.
Monographs	Canadian:<br>GFR < 30 mL/min/1.73 m^2: contraindicated.<br>American:<br>Monitor for risk of hyperkalemia.
RPT	FDA approved product labeling:<br>Monitor for risk of hyperkalemia.<br>Alternative Adjustment:<br>GFR < 30 mL/min: reno-protective effects with 25 mg once daily; monitor for hyperkalemia.

Non-Steroidal Mineralocorticoid Receptor Antagonist

**Resources**	Finerenone
CPS, Lexicomp, Micromedex, Monographs	eGFR > 25 – < 60 mL/min/m²: 10 mg once daily initially.<br>eGFR <25 mL/min/m²: Use not recommended.
RPT	No dosing information from this resource.

CGRP Antagonists

<table>
<tr><th>Resources</th><th>Atogepant</th></tr>
<tr><td>CPS</td><td>No dosing recommendation from this resource.</td></tr>
<tr><td>Lexicomp
Micromedex</td><td><table><tr><th></th><th>Chronic Migraine prevention</th><th>Episodic Migraine Prevention</th></tr><tr><td>CrCl < 30 mL/min</td><td>Avoid use</td><td>10 mg once daily</td></tr></table></td></tr>
<tr><td>Monographs</td><td>Canadian: CrCl < 30 mL/min: 10 mg daily.
American: CrCl < 30 mL/min: 10 mg daily.</td></tr>
<tr><td>RPT</td><td>No dosing recommendation from this resource.</td></tr>
</table>

**Resources**	Ubrogepant
CPS	No dosing recommendation from this resource.
Lexicomp<br>Monographs<br>RPT	CrCl 15 – 29 mL/min: 50 mg as a single dose, may repeat dose after 2 hours; maximum 100 mg daily.<br>CrCl < 15 mL/min: avoid use.
Micromedex	CrCl 15 – 29 mL/min: 50 mg as a single dose, may repeat dose after 2 hours.<br>CrCl < 15 mL/min: avoid use.

Other Drugs

**Resource**	Baclofen
CPS	Renal impairment, not on dialysis: use with caution, and generally with a reduced dose.<br>ESRD, not on dialysis: only administer if expected benefits are acceptable when considering potential risks.
Lexicomp	CrCl < 30 mL/min: avoid. If cannot be avoided, initiate at 2.5 mg q12h or less, titrating with extreme caution. Do not exceed 20 mg daily or ~33% of usual maximum daily dose (whichever is less).
Micromedex	CrCl < 30 mL/min: reduce dose by 2/3.
Monographs	Canadian:<br>Renal impairment, not on dialysis: use with caution, and generally with a reduced dose.<br>ESRD, not on dialysis: only administer if expected benefits are acceptable when considering potential risks.<br>American:<br>Impaired renal function: use with caution, dose may need to be reduced.
RPT	FDA-approved product labeling:<br>Kidney disease: use with caution, may be necessary to reduce dose.<br>Alternative adjustment:<br>GFR < 30 mL/min, not on dialysis: 5 mg/day in divided doses.

**Resource**	Lithium
CPS	eGFR 10 – 50 mL/min: 25 – 50% dose reduction.<br>eGFR < 10 mL/min: 50 – 75% dose reduction.
Lexicomp	CrCl < 30 mL/min: avoid.
Micromedex	CrCl < 30 mL/min: not recommended.
Monographs	Canadian:<br>Significant renal disease: contraindicated.<br>American:<br>Significant renal disease: contraindicated.
Renal Pharmaco-therapy	FDA-approved product labeling:<br>CrCl < 30 mL/min: should not be used; increased risk of lithium intoxication.<br>Alternative adjustment:<br>GFR 15 – 59 mL/min: 300 – 600 mg/day in divided doses (25 – 50% dose reduction).<br>GFR < 15 mL/min: 150 – 450 mg/day in divided doses (50 – 75% dose reduction).

**Resource**	Metoclopramide
CPS	CrCl 10 – 50 mL/min: 75% of usual dose.<br>CrCl < 10 mL/min: 50% of usual dose.
Lexicomp	CrCl > 10 – 60 mL/min: ~50% of usual total daily dose.<br>CrCl ≤ 10 mL/min: ~33% (or less) of usual total daily dose.
Micromedex	*Diabetic Gastroparesis:*<br>CrCl < 60 mL/min: 5 mg QID (30 minutes before each meal and at bedtime); maximum 20 mg/day.<br>ESRD: 5 mg BID; maximum 10 mg/day.<br>*GERD:*<br>CrCl < 60 mL/min: 5 mg QID (30 minutes before each meal and at bedtime) OR 10 mg TID; maximum 30 mg/day.<br>ESRD: 5 mg QID (30 minutes before each meal and at bedtime) OR 10 mg BID; maximum 20 mg/day.
Monographs	Canadian:<br>CrCl < 60 mL/min: dose should be reduced.<br>American:<br>*Diabetic Gastroparesis:*<br>CrCl < 60 mL/min: 5 mg QID (30 minutes before each meal and at bedtime); maximum 20 mg/day.<br>ESRD: 5 mg BID; maximum 10 mg/day.<br>GERD:<br>CrCl ≤ 60 mL/min: 5 mg QID (30 minutes before each meal and at bedtime) OR 10 mg TID; maximum 30 mg/day.<br>ESRD: 5 mg QID (30 minutes before each meal and at bedtime) OR 10 mg BID; maximum 20 mg/day.
RPT	FDA-approved product labeling:<br>CrCl < 40 mg/dL: initiate at 50% recommended dosage, may be increased or decreased depending on efficacy and safety.<br>Alternative adjustment:<br>GFR 15 – 59 mL/min: 7.5 mg QID.<br>GFR > 15 mL/min: 5 mg QID.

Resource	Digoxin

CPS

Usual Daily Maintenance Dose Requirements (mg):

Corrected CrCl (mL/min per 70 kg)	Lean Body Weight (kg)					
	50	60	70	80	90	100
0	0.0625	0.125	0.125	0.125	0.1875	0.1875
10	0.125	0.125	0.125	0.1875	0.1875	0.1875
20	0.125	0.125	0.1875	0.1875	0.1875	0.25
30	0.125	0.1875	0.1875	0.1875	0.25	0.25

Lexicomp

Atrial Fibrillation:

Maintenance Dose:
CrCl < 30 mL/min: 0.0625 mg q48h or alternative agent.
Heart Failure:

Ideal Body Weight (kg)	CrCl (mL/min)	Digoxin Dose
45 – 50	15 – 60	0.0625 mg daily
> 50 – 60	15 – 45	0.0625 mg daily
> 60 – 70	15 – 35	0.0625 mg daily
> 70 – 80	> 20 – 80	0.125 mg daily
	15 – 20	0.0625 mg daily
> 80	15 – 70	0.125 mg daily

Monographs

Canadian:
Renal Insufficiency: Lower maintenance doses needed.
Usual Daily Maintenance Dose Requirements (mg):

Corrected CrCl (mL/min per 70 kg)	Body Weight (kg)					
	50	60	70	80	90	100
0	0.0625	0.125	0.125	0.125	0.1875	0.1875
10	0.125	0.125	0.125	0.1875	0.1875	0.1875
20	0.125	0.125	0.1875	0.1875	0.1875	0.25
30	0.125	0.1875	0.1875	0.1875	0.25	0.25

American:
Renal impairment: reduce and titrate dose carefully according to clinical response and serum concentrations.
Recommended Daily Maintenance Dose (mg):

Corrected CrCl (mL/min per 70 kg)	Lean Body Weight (kg)						
	40	50	60	70	80	90	100
10	0.0625	0.125	0.125	0.1875	0.1875	0.1875	0.25
20	0.125	0.125	0.125	0.1875	0.1875	0.25	0.25

<table>
<tr><td></td><td>
<table>
<tr><td>30</td><td>0.125</td><td>0.125</td><td>0.1875</td><td>0.1875</td><td>0.25</td><td>0.25</td><td>0.3125</td></tr>
</table>
</td></tr>
<tr><td>RPT</td><td>FDA-approved product labeling:
Usual Daily Maintenance Dose Requirements (mg):
<table>
<tr><th rowspan="2">CrCl (mL/min)</th><th colspan="6">Lean Body Weight (kg)</th></tr>
<tr><th>50</th><th>60</th><th>70</th><th>80</th><th>90</th><th>100</th></tr>
<tr><td>0</td><td>0.0625</td><td>0.125</td><td>0.125</td><td>0.125</td><td>0.1875</td><td>0.1875</td></tr>
<tr><td>10</td><td>0.125</td><td>0.125</td><td>0.125</td><td>0.1875</td><td>0.1875</td><td>0.1875</td></tr>
<tr><td>20</td><td>0.125</td><td>0.125</td><td>0.1875</td><td>0.1875</td><td>0.1875</td><td>0.25</td></tr>
<tr><td>30</td><td>0.125</td><td>0.1875</td><td>0.1875</td><td>0.1875</td><td>0.25</td><td>0.25</td></tr>
</table>
Alternative adjustment:
GFR 15 – 59 mL/min: 0.0625 mg q24 –36h.
GFR < 15 mL/min: 0.0625 mg q48h.</td></tr>
</table>

<table>
<tr><th>Resources</th><th>Methotrexate</th></tr>
<tr><td>CPS</td><td>
<table>
<tr><td>Rheumatoid Arthritis</td><td>CrCl < 30mL/min</td><td>Avoid</td></tr>
</table>
</td></tr>
<tr><td>Lexicomp</td><td>CrCl 10 – 50 mL/min: 50% dose adjustment.
CrCl <10 mL/min: avoid use.</td></tr>
<tr><td>Micromedex</td><td>GFR 20 –50 mL/min: 50% original dose.
GFR <20 mL/min: avoid use.</td></tr>
<tr><td>Monograph</td><td>Canadian:
CrCl <50 mL/min: use alternative therapy.
American:
Reduce the dosage or discontinue as appropriate.</td></tr>
<tr><td>RPT</td><td>FDA Approved Labeling: Should only be used if benefit outweigh risk.
Alternative Adjustment:
GFR 15 – 59 mL/min: 2.5 mg orally once weekly.
GFR < 15 mL/min: avoid unless no suitable alternative exists; 2.5 – 5 mg orally once weekly.</td></tr>
</table>

**Resources**	Tizanidine
CPS	CrCl <25 mL/min: use with caution, reduce dose.
Lexicomp	CrCl >25 - < 60 mL/min: use with caution.<br>CrCl <25 mL/min: initial: 2 mg once daily, may increase based on response and tolerability by 2 mg oer day (minimum 1 to 4 days between dose increases).
Micromedex	CrCl < 25 mL/min: reduce individual doses of tizanidine during titration.
Monographs	Canadian & American:<br>CrCl < 25 mL/min: reduce individual doses during titration; if higher doses are required individual doses rather than frequency should be increased.
RPT	FDA – approved product labeling:<br>CrCl > 25 mL/min: initial 2 mg every 6 – 8 hours as needed, maximum three doses in 24 hours; increase dose gradually by 2 – 4 mg with 1 to 4 days between dosage increases. Total daily dose not to exceed 36 mg.<br>CrCl <25 mL/min: reduce individual doses during titration; if higher doses are required individual doses rather than frequency should be increased.<br>Alternative Adjustment:<br>GFR > 25 mL/min: 2– 8 mg orally every 6 – 8 hours as necessary or 4 – 8mg sublingually at bedtime.<br>GFR <25 mL/min: 2 mg orally once or twice daily; titrate according to response and tolerance.

**Resources**	Risperidone
CPS	Renal Impairment: starting and consecutive dosing should be halved.<br>Recommended initial dose is 0.5 mg BID and dosage increases should be in increments of no more than 0.5 mg BID. Increases to dosages above 1.5 mg BID at intervals of 1 week.
Lexicomp	CrCl 10 - <30 mL/min: initially 50% of usual dosage, titrate in increments of no more than 0.5 mg BID; doses greater than 1.5 mg BID should be titrated at intervals of 1 week.
Micromedex	CrCl < 30 mL/min: initial 0.5 mg BI, doses may be increased by 0.5 mg BID; increases in dosages above 1.5 mg BID should be done at intervals of 1 week.
Monographs	Canadian:<br>Recommended initial dose is 0.5 mg BID and dosage increases should be in increments of no more than 0.5 mg BID. Increases to dosages above 1.5 mg BID at intervals of 1 week.<br>American:<br>CrCl < 30 mL/min: initial 0.5 mg BI, doses may be increased by 0.5 mg BID; increases in dosages above 1.5 mg BID should be done at intervals of 1 week.
RPT	FDA- Approved Product Labeling:<br>CrCl <30 mL/min: 0.5 mg orally BID; may increase in increments of 0.5 mg or less BID; for doses >1.5 mg BID, increase in intervals of 1 week or greater.<br>Alternative Dosing:<br>GFR 15- 59 mL/min: 0.5 – 2 mg orally twice daily.<br>GFR <15 mL/min: 0.5 – 2 mg orally once daily; begin modestly and titrate carefully.

**Resources**	Abrocitinib
CPS	No dosing information available from this resource.
Lexicomp	American labeling:<br>eGFR < 30 mL/min: use not recommended.<br>Canadian labeling:<br>eGFR < 60 mL/min: reduce dose by 50%.
Micromedex	eGFR 15 – 29 mL/min: use not recommended.
Monographs	Canadian:<br>eGFR<30 mL/min: reduce dose by 50%.<br>American:<br>eGFR <30 mL/min: use not recommended.
RPT	No dosing information available from this resource.

**Resources**	Varenicline
CPS	CrCl <30 mL/min: initiate at 0.5 mg once daily for the first 3 days then increase to 0.5 mg BID as maintenance dose.
Lexicomp	CrCl <30 mL/min: initial of 0.5 mg once daily; maximum maintenance dose of 0.5 mg twice daily.
Micromedex	CrCl < 30 mL/min: initial dose 0.5 mg orally once daily; may increase to 0.5 mg BID.
Monographs	Canadian: CrCl < 30 mL/min: initial dose 0.5 mg once daily for 3 days, increase to 0.5 mg BID.<br>American: CrCl <30 mL/min: initiate at 0.5 mg once daily then titrate to 0.5 mg BID.
RPT	FDA – Approved Labeling:<br>CrCl < 30 mL/min: 0.5 mg orally once daily and titrate to 0.5 mg BID.

<table>
<tr><th>Resources</th><th>Sildenafil</th></tr>
<tr><td>CPS</td><td>CrCl < 30 mL/min: starting dose of 25 mg.</td></tr>
<tr><td>Lexicomp
Micromedex</td><td><table><tr><td></td><td>Erectile Dysfunction (ED)</td></tr><tr><td>CrCl < 30 mL/min</td><td>Initial dose of 25 mg once daily prn 1 hour before sexual activity; maximum 100 mg daily titrated cautiously</td></tr></table></td></tr>
<tr><td>Monographs</td><td>Canadian CrCl < 30 mL/min: initiate dose at 25 mg daily.
American CrCl <30 mL/min: initiate dose at 25 mg daily.</td></tr>
<tr><td>RPT</td><td>CrCl < 30 mL/min: caution, consider starting at 25 mg.</td></tr>
</table>

<table>
<tr><th>Resources</th><th>Tadalafil</th></tr>
<tr><td>CPS</td><td>CrCl <30 mL/min: caution for on demand dosing; initiate 10 mg prior to anticipated sexual activity, no more than on alternate days not to exceed 3 times a week.
CrCl < 30 mL/min: not recommended for daily dosing (ex./ BPH treatment).</td></tr>
<tr><td>Lexicomp</td><td><table>
<tr><th></th><th>BPH, ED (daily dosing)</th><th>ED (on-demand)</th></tr>
<tr><td>CrCl < 30 mL/min</td><td>avoid</td><td>5 mg 30 min before sexual activity; repeat no more than every 72 hours</td></tr>
</table></td></tr>
<tr><td>Micromedex</td><td><table>
<tr><th></th><th>BPH, ED (daily dosing)</th><th>ED (on-demand)</th></tr>
<tr><td>CrCl < 30 mL/min</td><td>avoid</td><td>5 mg daily; repeat no more than every 72 hours</td></tr>
</table></td></tr>
<tr><td>Monographs</td><td>Canadian:
CrCl <30 mL/min: Daily dosing not recommended.
American:
CrCl < 30 mL/min: 5 mg prn every 72 hours.</td></tr>
<tr><td>RPT</td><td>FDA – approved product labeling:<table>
<tr><th></th><th>BPH</th><th>ED</th></tr>
<tr><td>CrCl <30 mL/min</td><td>Maximum 5 mg every 72 hours</td><td>Maximum 5 mg every 72 hours</td></tr>
</table></td></tr>
</table>

Resource	Sotalol
CPS	CrCl 10-30 mL/min: increase dosing interval to 36 – 48 hours.<br>CrCl < 10 mL/min: individualize.
Lexicomp	Atrial Fibrillation:<br>CrCl < 40 mL/min: contraindicated.<br>Ventricular arrhythmia:<br>CrCl 10-29 mL/min: administer every 36-48 hours.<br>CrCl < 10 mL/min: avoid use.
Micromedex	Administer initial dose of 80 mg then:<br>CrCl 10 – 29 mL/min every 36 – 48 hours.<br>CrCl < 10 mL/min: individualize dosing.
Monographs	Canadian:<br>CrCl 10-30 mL/min: dosing interval every 36-48 hours.<br>CrCl < 10 mL/min: individualize dosing.<br>American:<br>CrCl 10-30 mL/min: dosing interval 48 hours.
RPT	FDA – approved product labeling:<br>Ventricular Arrhythmias<br>CrCl: 10-29 mL/min 80 mg every 36 – 48 hours.<br>CrCl < 10 mL/min: individualize.<br>Atrial Fibrillation:<br>CrCl< 40 mL/min: contraindicated.<br>Alternative adjustment:<br>GFR 15-59 mL/min: 80 – 160 mg orally every 24 – 48 hours.<br>GFR < 15 mL/min: 80-160 mg every 48 – 72 hours.

<table>
<tr><th>Resources</th><th>Amantadine</th></tr>
<tr><td>CPS</td><td>
<table>
<tr><th>CrCl (mL/min/1.73m^2)</th><th><65 years</th><th>≥ 65 years</th></tr>
<tr><td>15 – 29</td><td>200 mg 1 day, then 100 mg every other day</td><td>100 mg every other day</td></tr>
<tr><td><15</td><td>200 mg every 7 days</td><td>100 mg every 7 days</td></tr>
</table>
</td></tr>
<tr><td>Lexicomp</td><td>Immediate Release:
CrCl 15 – <30 mL/min: 200 mg on day 1, then 100 mg every other day.
CrCl < 15 mL/min: 200 mg every 7 days.</td></tr>
<tr><td>Micromedex</td><td>Immediate Release:
CrCl 15 – <30 mL/min/1.73 m^2: 200 mg on day 1, then 100 mg every other day.
CrCl < 15 mL/min/1.73 m^2: 200 mg every 7 days.</td></tr>
<tr><td>Monographs</td><td>Canadian
CrCl 15 – 35 mL/min: 100mg every 2-3 days.
CrCl <15mL/min : contraindicated.
American
CrCl 15 – <30 mL/min/1.73 m2 : 200 mg on day 1, then 100 mg every other day.
CrCl < 15 mL/min/1.73 m2 : 200 mg every 7 days.</td></tr>
<tr><td>RPT</td><td>FDA Approved product labeling:
Amantadine Immediate Release:
CrCl 15 – 29 mL/min: 200 mg on day 1 followed by 100 mg every other day.
CrCl < 15 mL: 200 mg orally every 7 days.
Alternative Adjustment:
GFR 15 – 59 mL/min: 100 mg orally ever 24 – 48 hours.
GFR < 15 mL/min 100 mg every 7 days.</td></tr>
</table>

<table>
<tr><th>Resources</th><th>Memantine</th></tr>
<tr><td>CPS</td><td>CrCl 15-29 mL/min: 10 mg daily.</td></tr>
<tr><td>Lexicomp</td><td>Immediate Release:
CrCl <30 mL/min: initial 5 mg once daily, may increase to 5 mg BID after 1 week.</td></tr>
<tr><td>Micromedex
RPT</td><td>Immediate Release:
CrCl 5 – 29 mL/min: 5 mg bid.</td></tr>
<tr><td>Monographs</td><td>
<table>
<tr><th></th><th>Immediate Release</th></tr>
<tr><td>Canadian (15 – 29 mL/min)</td><td>10 mg daily</td></tr>
<tr><td>American (CrCl 5 – 29 mL/min)</td><td>5 mg BID</td></tr>
</table>
</td></tr>
</table>
