# Supplementary material for: Medications for community pharmacists to dose adjust or avoid to enhance prescribing safety in individuals with advanced chronic kidney disease: a scoping review and modified Delphi
Source: BMC Nephrol. 2024 Oct 29;25:386. doi: 10.1186/s12882-024-03829-y (PMC11523796; doi:10.1186/s12882-024-03829-y)
Supplement: Supplementary file 3 — Additionsl file 3: Modified Delphi Survey Rounds 1, 2, 3. [file 12882_2024_3829_MOESM3_ESM.pdf]

Round 1: Modified Delphi Survey - Drugs to Adjust\Avoid in Non-Dialysis CKD eGFR below 30

Background

Chronic kidney disease (CKD) is a common condition in primary care. The kidneys are responsible for the removal of many drugs from the body. These drugs may require adjustment to avoid buildup which can be harmful. Individuals with CKD tend to have multiple comorbidities, are older and taking many medications. Considering these factors the risk for unwanted drug effects or harm are high. Community pharmacists commonly see individuals with CKD and are in an ideal position to protect or preserve kidney function through appropriate prescribing.

Purpose

The purpose of this research is to create a pragmatic list of medications to adjust or avoid in individuals with advanced non-dialysis dependent kidney disease (eGFR <30 mL/min) by primary care community pharmacists. The long-term goal is to develop an electronic drug dosing tool of these medications for community pharmacists which:

1.Increases awareness of higher-risk medications used in CKD.

1.Decreases risk of exposure to nephrotoxic medications that can lower kidney function.

1.Provides evidence and expert informed dosing recommendations.

Survey Instructions:

1. A **drug dosing resource** of Round 1 Modifed Delphi drugs has been e-mailed to you. This drug dosing resource may assist you in completing the survey questions.

2. At the end of the survey, there will be space to **add additional medications** which were not included in Round 1 that you believe are important for consideration for Round 2 of the Modified Delphi process.

3. For each medication in the survey, please **select** from a drop-down menu whether you would **dose adjust or avoid** based on the eGFR category (15-29 mL/min and <15 mL/min). Rate your level of agreement on a 5-point Likert scale (1= strongly disagree, 2 = disagree, 3 = neither agree or disagree, 4 = agree, 5 = strongly agree) on the importance of community pharmacists adjusting or avoiding the select medication.

4. This survey will take approximately 60 minutes to complete. Please ensure you add your email address in the box provided at the end of the survey and select 'Finish' to recieve your individual responses. Please **complete** Round 1 of the Modified Delphi Survey by **Monday July 8, 2024**.

Thank you very much for your time.

The second round will follow shortly. (Anticipated July 10, 2024).

Please Note: For the purposes of this Modified Delphi, eGFR (mL/min) is being used as it is the preferred method to evaluate kidney function.

Reach out if you have any questions.

Dr. Jo-Anne Wilson, Research Lead, jo-anne.wilson@dal.ca

Natalie Ratajczak, Pharmacy Research Assistant, natalieratajczak@dal.ca

Demographics

Q1: Which province do you practice as a pharmacist?

- ◯ Alberta
- ◯ British Columbia
- ◯ Manitoba
- ◯ New Brunswick
- ◯ Newfoundland and Labrador
- ◯ Nova Scotia
- ◯ Ontario
- ◯ Prince Edward Island
- ◯ Quebec
- ◯ Saskachewan

Q2: How many years have you practiced as a pharmacist?

◯ 5-10 ◯ 11-15 ◯ 16-20 ◯ 20+

Q3: What is your area of practice?

- ◯ Nephrology
- ◯ Geriatric
- ◯ Primary Care
- ◯ Other - Please specify below

Q4: How many years have you practiced based on your response in question 3?

◯ 5-10 ◯ 11-15 ◯ 16-20 ◯ 20+

Q5: What is your highest level of education achieved?

- ◯ Bachelor of Pharmacy
- ◯ Undergraduate PharmD
- ◯ Masters Degree
- ◯ Post-Graduate PharmD
- ◯ Other - Please provide your answer in the space below

Q6: Approximately how many patients per month with an eGFR<30 mL/min do you provide medication management?

◯ 0-10 ◯ 11-20 ◯ 21-30 ◯ 31-40 ◯ 41-50 ◯ >50

For each medication in the survey, please **select** whether you would **dose adjust or avoid** based on the eGFR category (15-29 mL/min and <15 mL/min). Rate your level of agreement on a 5-point Likert scale (1= strongly disagree, 2 = disagree, 3 = neither agree or disagree, 4 = agree, 5 = strongly agree) on the importance of community pharmacists adjusting or avoiding the select medication.

Antihyperglycemics

Q7: Metformin

eGFR 15-29	1	2	3	4	5
Select one<br>◯ Dose ADJUST<br>◯ Dose AVOID	◯	◯	◯	◯	◯
eGFR <15	1	2	3	4	5
Select one<br>◯ Dose ADJUST<br>◯ Dose AVOID	◯	◯	◯	◯	◯

Q8: Glyburide

eGFR 15-29	1	2	3	4	5
Select one<br>◯ Dose ADJUST<br>◯ Dose AVOID	◯	◯	◯	◯	◯
eGFR <15	1	2	3	4	5

Select one<br>◯ Dose ADJUST<br>◯ Dose AVOID	◯	◯	◯	◯	◯

Q9: Saxagliptin

eGFR 15-29	1	2	3	4	5
Select one<br>◯ Dose ADJUST<br>◯ Dose AVOID	◯	◯	◯	◯	◯
eGFR <15	1	2	3	4	5
Select one<br>◯ Dose ADJUST<br>◯ Dose AVOID	◯	◯	◯	◯	◯

Q10: Sitagliptin

eGFR 15-29	1	2	3	4	5
Select one<br>◯ Dose ADJUST<br>◯ Dose AVOID	◯	◯	◯	◯	◯
eGFR <15	1	2	3	4	5
Select one<br>◯ Dose ADJUST<br>◯ Dose AVOID	◯	◯	◯	◯	◯

For each medication in the survey, please **select** whether you would **dose adjust or avoid** based on the eGFR category (15-29 mL/min and <15 mL/min). Rate your level of agreement on a 5-point Likert scale (1= strongly disagree, 2 = disagree, 3 = neither agree or disagree, 4 = agree, 5 = strongly agree) on the importance of community pharmacists adjusting or avoiding the select medication.

Lipid Lowering Agents

Q11: Bezafibrate

eGFR 15-29	1	2	3	4	5
Select one<br>◯ Dose ADJUST<br>◯ Dose AVOID	◯	◯	◯	◯	◯
eGFR <15	1	2	3	4	5
Select one<br>◯ Dose ADJUST<br>◯ Dose AVOID	◯	◯	◯	◯	◯

Q12: Fenofibrate

eGFR 15-29	1	2	3	4	5
Select one<br>◯ Dose ADJUST<br>◯ Dose AVOID	◯	◯	◯	◯	◯
eGFR <15	1	2	3	4	5
Select one<br>◯ Dose ADJUST<br>◯ Dose AVOID	◯	◯	◯	◯	◯

Q13: Rosuvastatin

eGFR 15-29	1	2	3	4	5
Select one<br>◯ Dose ADJUST<br>◯ Dose AVOID	◯	◯	◯	◯	◯
eGFR <15	1	2	3	4	5
Select one<br>◯ Dose ADJUST<br>◯ Dose AVOID	◯	◯	◯	◯	◯

For each medication in the survey, please **select** whether you would **dose adjust or avoid** based on the eGFR category (15-29 mL/min and <15 mL/min). Rate your level of agreement on a 5-point Likert scale (1= strongly disagree, 2 = disagree, 3 = neither agree or disagree, 4 = agree, 5 = strongly agree) on the importance of community pharmacists adjusting or avoiding the select medication.

Antimuscarinics

Q14: Solifenacin

eGFR 15-29	1	2	3	4	5
Select one<br>◯ Dose ADJUST<br>◯ Dose AVOID	◯	◯	◯	◯	◯
eGFR <15	1	2	3	4	5
Select one<br>◯ Dose ADJUST<br>◯ Dose AVOID	◯	◯	◯	◯	◯

Q15: Tolterodine

eGFR 15-29	1	2	3	4	5
Select one<br>◯ Dose ADJUST<br>◯ Dose AVOID	◯	◯	◯	◯	◯
eGFR <15	1	2	3	4	5
Select one<br>◯ Dose ADJUST<br>◯ Dose AVOID	◯	◯	◯	◯	◯

For each medication in the survey, please **select** whether you would **dose adjust or avoid** based on the eGFR category (15-29 mL/min and <15 mL/min). Rate your level of agreement on a 5-point Likert scale (1= strongly disagree, 2 = disagree, 3 = neither agree or disagree, 4 = agree, 5 = strongly agree) on the importance of community pharmacists adjusting or avoiding the select medication.

Anticonvulsants

Q16: Gabapentin

eGFR 15-29	1	2	3	4	5
Select one<br>◯ Dose ADJUST<br>◯ Dose AVOID	◯	◯	◯	◯	◯
eGFR <15	1	2	3	4	5
Select one<br>◯ Dose ADJUST<br>◯ Dose AVOID	◯	◯	◯	◯	◯

Q17: Pregabalin

eGFR 15-29	1	2	3	4	5
Select one<br>◯ Dose ADJUST<br>◯ Dose AVOID	◯	◯	◯	◯	◯
eGFR <15	1	2	3	4	5
Select one<br>◯ Dose ADJUST<br>◯ Dose AVOID	◯	◯	◯	◯	◯

Q18: Topiramate

eGFR 15-29	1	2	3	4	5
Select one<br>◯ Dose ADJUST<br>◯ Dose AVOID	◯	◯	◯	◯	◯
eGFR <15	1	2	3	4	5
Select one<br>◯ Dose ADJUST<br>◯ Dose AVOID	◯	◯	◯	◯	◯

Q19: Cenobamate

eGFR 15-29	1	2	3	4	5
Select one<br>◯ Dose ADJUST<br>◯ Dose AVOID	◯	◯	◯	◯	◯
eGFR <15	1	2	3	4	5
Select one<br>◯ Dose ADJUST<br>◯ Dose AVOID	◯	◯	◯	◯	◯

For each medication in the survey, please **select** whether you would **dose adjust or avoid** based on the eGFR category (15-29 mL/min and <15 mL/min). Rate your level of agreement on a 5-point Likert scale (1= strongly disagree, 2 = disagree, 3 = neither agree or disagree, 4 = agree, 5 = strongly agree) on the importance of community pharmacists adjusting or avoiding the select medication.

Urate Lowering Agents

Q20: Allopurinol

eGFR 15-29	1	2	3	4	5
Select one<br>◯ Dose ADJUST<br>◯ Dose AVOID	◯	◯	◯	◯	◯
eGFR <15	1	2	3	4	5
Select one<br>◯ Dose ADJUST<br>◯ Dose AVOID	◯	◯	◯	◯	◯

Q21: Colchicine

eGFR 15-29	1	2	3	4	5
Select one<br>○ Dose ADJUST<br>○ Dose AVOID	○	○	○	○	○
eGFR <15	1	2	3	4	5
Select one<br>○ Dose ADJUST<br>○ Dose AVOID	○	○	○	○	○

Q22: Febuxostat

eGFR 15-29	1	2	3	4	5
Select one<br>○ Dose ADJUST<br>○ Dose AVOID	○	○	○	○	○
eGFR <15	1	2	3	4	5
Select one<br>○ Dose ADJUST<br>○ Dose AVOID	○	○	○	○	○

For each medication in the survey, please **select** whether you would **dose adjust or avoid** based on the eGFR category (15-29 mL/min and <15 mL/min). Rate your level of agreement on a 5-point Likert scale (1= strongly disagree, 2 = disagree, 3 = neither agree or disagree, 4 = agree, 5 = strongly agree) on the importance of community pharmacists adjusting or avoiding the select medication.

Q23: Apixaban

eGFR 15-29	1	2	3	4	5
Select one<br>◯ Dose ADJUST<br>◯ Dose AVOID	◯	◯	◯	◯	◯
eGFR <15	1	2	3	4	5
Select one<br>◯ Dose ADJUST<br>◯ Dose AVOID	◯	◯	◯	◯	◯

Q24: Dabigatran

eGFR 15-29	1	2	3	4	5
Select one<br>◯ Dose ADJUST<br>◯ Dose AVOID	◯	◯	◯	◯	◯
eGFR <15	1	2	3	4	5
Select one<br>◯ Dose ADJUST<br>◯ Dose AVOID	◯	◯	◯	◯	◯

Q25: Edoxaban

eGFR 15-29	1	2	3	4	5
Select one<br>◯ Dose ADJUST<br>◯ Dose AVOID	◯	◯	◯	◯	◯
eGFR <15	1	2	3	4	5
Select one<br>◯ Dose ADJUST<br>◯ Dose AVOID	◯	◯	◯	◯	◯

Q26: Rivaroxaban

eGFR 15-29	1	2	3	4	5
Select one<br>◯ Dose ADJUST<br>◯ Dose AVOID	◯	◯	◯	◯	◯
eGFR <15	1	2	3	4	5
Select one<br>◯ Dose ADJUST<br>◯ Dose AVOID	◯	◯	◯	◯	◯

For each medication in the survey, please **select** whether you would **dose adjust or avoid** based on the eGFR category (15-29 mL/min and <15 mL/min). Rate your level of agreement on a 5-point Likert scale (1= strongly disagree, 2 = disagree, 3 = neither agree or disagree, 4 = agree, 5 = strongly agree) on the importance of community pharmacists adjusting or avoiding the select medication.

Low Molecular Weight Heparins for VTE Treatment

Q27: Dalteparin

eGFR 15-29	1	2	3	4	5
Select one<br>○ Dose ADJUST<br>○ Dose AVOID	○	○	○	○	○
eGFR <15	1	2	3	4	5
Select one<br>○ Dose ADJUST<br>○ Dose AVOID	○	○	○	○	○

Q28: Tinzaparin

eGFR 15-29	1	2	3	4	5
Select one<br>○ Dose ADJUST<br>○ Dose AVOID	○	○	○	○	○
eGFR <15	1	2	3	4	5
Select one<br>○ Dose ADJUST<br>○ Dose AVOID	○	○	○	○	○

Q29: Enoxaparin

eGFR 15-29	1	2	3	4	5
Select one<br>◯ Dose ADJUST<br>◯ Dose AVOID	◯	◯	◯	◯	◯
eGFR <15	1	2	3	4	5
Select one<br>◯ Dose ADJUST<br>◯ Dose AVOID	◯	◯	◯	◯	◯

For each medication in the survey, please **select** whether you would **dose adjust or avoid** based on the eGFR category (15-29 mL/min and <15 mL/min). Rate your level of agreement on a 5-point Likert scale (1= strongly disagree, 2 = disagree, 3 = neither agree or disagree, 4 = agree, 5 = strongly agree) on the importance of community pharmacists adjusting or avoiding the select medication.

Antivirals

Q30: Acyclovir

eGFR 15-29	1	2	3	4	5
Select one<br>◯ Dose ADJUST<br>◯ Dose AVOID	◯	◯	◯	◯	◯
eGFR <15	1	2	3	4	5
Select one<br>◯ Dose ADJUST<br>◯ Dose AVOID	◯	◯	◯	◯	◯

Q31: Famciclovir

eGFR 15-29	1	2	3	4	5
Select one<br>◯ Dose ADJUST<br>◯ Dose AVOID	◯	◯	◯	◯	◯
eGFR <15	1	2	3	4	5
Select one<br>◯ Dose ADJUST<br>◯ Dose AVOID	◯	◯	◯	◯	◯

Q32: Valacyclovir

eGFR 15-29	1	2	3	4	5
Select one<br>◯ Dose ADJUST<br>◯ Dose AVOID	◯	◯	◯	◯	◯
eGFR <15	1	2	3	4	5
Select one<br>◯ Dose ADJUST<br>◯ Dose AVOID	◯	◯	◯	◯	◯

Q33: Oseltamivir

eGFR 15-29	1	2	3	4	5
Select one<br>◯ Dose ADJUST<br>◯ Dose AVOID	◯	◯	◯	◯	◯
eGFR <15	1	2	3	4	5
Select one<br>◯ Dose ADJUST<br>◯ Dose AVOID	◯	◯	◯	◯	◯

Q34: Paxlovid (nirmatrelvir/ritonavir)

eGFR 15-29	1	2	3	4	5
Select one<br>◯ Dose ADJUST<br>◯ Dose AVOID	◯	◯	◯	◯	◯
eGFR <15	1	2	3	4	5
Select one<br>◯ Dose ADJUST<br>◯ Dose AVOID	◯	◯	◯	◯	◯

Q35: Truvada (emtricitabine / tenofovir disoproxil fumarate)

eGFR 15-29	1	2	3	4	5
Select one<br>◯ Dose ADJUST<br>◯ Dose AVOID	◯	◯	◯	◯	◯

eGFR <15	1	2	3	4	5
Select one<br>◯ Dose ADJUST<br>◯ Dose AVOID	◯	◯	◯	◯	◯

For each medication in the survey, please **select** whether you would **dose adjust or avoid** based on the eGFR category (15-29 mL/min and <15 mL/min). Rate your level of agreement on a 5-point Likert scale (1= strongly disagree, 2 = disagree, 3 = neither agree or disagree, 4 = agree, 5 = strongly agree) on the importance of community pharmacists adjusting or avoiding the select medication.

Antibiotics

Q36: Sulfamethoxazole/Trimethoprim

eGFR 15-29	1	2	3	4	5
Select one<br>◯ Dose ADJUST<br>◯ Dose AVOID	◯	◯	◯	◯	◯
eGFR <15	1	2	3	4	5
Select one<br>◯ Dose ADJUST<br>◯ Dose AVOID	◯	◯	◯	◯	◯

Q37: Ciprofloxacin

eGFR 15-29	1	2	3	4	5
Select one<br>◯ Dose ADJUST<br>◯ Dose AVOID	◯	◯	◯	◯	◯

eGFR <15	1	2	3	4	5
Select one<br>◯ Dose ADJUST<br>◯ Dose AVOID	◯	◯	◯	◯	◯

Q38: Levofloxacin

eGFR 15-29	1	2	3	4	5
Select one<br>◯ Dose ADJUST<br>◯ Dose AVOID	◯	◯	◯	◯	◯
eGFR <15	1	2	3	4	5
Select one<br>◯ Dose ADJUST<br>◯ Dose AVOID	◯	◯	◯	◯	◯

Q39: Norfloxacin

eGFR 15-29	1	2	3	4	5
Select one<br>◯ Dose ADJUST<br>◯ Dose AVOID	◯	◯	◯	◯	◯
eGFR <15	1	2	3	4	5
Select one<br>◯ Dose ADJUST<br>◯ Dose AVOID	◯	◯	◯	◯	◯

Q40: Nitrofurantoin

eGFR 15-29	1	2	3	4	5
Select one<br>◯ Dose ADJUST<br>◯ Dose AVOID	◯	◯	◯	◯	◯
eGFR <15	1	2	3	4	5
Select one<br>◯ Dose ADJUST<br>◯ Dose AVOID	◯	◯	◯	◯	◯

For each medication in the survey, please **select** whether you would **dose adjust or avoid** based on the eGFR category (15-29 mL/min and <15 mL/min). Rate your level of agreement on a 5-point Likert scale (1= strongly disagree, 2 = disagree, 3 = neither agree or disagree, 4 = agree, 5 = strongly agree) on the importance of community pharmacists adjusting or avoiding the select medication.

Antifungals

Q41: Fluconazole

eGFR 15-29	1	2	3	4	5
Select one<br>◯ Dose ADJUST<br>◯ Dose AVOID	◯	◯	◯	◯	◯
eGFR <15	1	2	3	4	5
Select one<br>◯ Dose ADJUST<br>◯ Dose AVOID	◯	◯	◯	◯	◯

For each medication in the survey, please **select** whether you would **dose adjust or avoid** based on the eGFR category (15-29 mL/min and <15 mL/min). Rate your level of agreement on a 5-point Likert scale (1= strongly disagree, 2 = disagree, 3 = neither agree or disagree, 4 = agree, 5 = strongly agree) on the importance of community pharmacists adjusting or avoiding the select medication.

Analgesics / Opioids

Q42: Codeine

eGFR 15-29	1	2	3	4	5
Select one<br>◯ Dose ADJUST<br>◯ Dose AVOID	◯	◯	◯	◯	◯
eGFR <15	1	2	3	4	5
Select one<br>◯ Dose ADJUST<br>◯ Dose AVOID	◯	◯	◯	◯	◯

Q43: Morphine

eGFR 15-29	1	2	3	4	5
Select one<br>◯ Dose ADJUST<br>◯ Dose AVOID	◯	◯	◯	◯	◯
eGFR <15	1	2	3	4	5
Select one<br>◯ Dose ADJUST<br>◯ Dose AVOID	◯	◯	◯	◯	◯

Q44: Tramadol

eGFR 15-29	1	2	3	4	5
Select one<br>◯ Dose ADJUST<br>◯ Dose AVOID	◯	◯	◯	◯	◯
eGFR <15	1	2	3	4	5
Select one<br>◯ Dose ADJUST<br>◯ Dose AVOID	◯	◯	◯	◯	◯

Q45: NSAIDs

eGFR 15-29	1	2	3	4	5
Select one<br>◯ Dose ADJUST<br>◯ Dose AVOID	◯	◯	◯	◯	◯
eGFR <15	1	2	3	4	5
Select one<br>◯ Dose ADJUST<br>◯ Dose AVOID	◯	◯	◯	◯	◯

For each medication in the survey, please **select** whether you would **dose adjust or avoid** based on the eGFR category (15-29 mL/min and <15 mL/min). Rate your level of agreement on a 5-point Likert scale (1= strongly disagree, 2 = disagree, 3 = neither agree or disagree, 4 = agree, 5 = strongly agree) on the importance of community pharmacists adjusting or avoiding the select medication.

H2RA Antagonists

Q46: Ranitidine

eGFR 15-29	1	2	3	4	5
Select one<br>◯ Dose ADJUST<br>◯ Dose AVOID	◯	◯	◯	◯	◯
eGFR <15	1	2	3	4	5
Select one<br>◯ Dose ADJUST<br>◯ Dose AVOID	◯	◯	◯	◯	◯

Q47: Famotidine

eGFR 15-29	1	2	3	4	5
Select one<br>◯ Dose ADJUST<br>◯ Dose AVOID	◯	◯	◯	◯	◯
eGFR <15	1	2	3	4	5
Select one<br>◯ Dose ADJUST<br>◯ Dose AVOID	◯	◯	◯	◯	◯

For each medication in the survey, please **select** whether you would **dose adjust or avoid** based on the eGFR category (15-29 mL/min and <15 mL/min). Rate your level of agreement on a 5-point Likert scale (1= strongly disagree, 2 = disagree, 3 = neither agree or disagree, 4 = agree, 5 = strongly agree) on the importance of community pharmacists adjusting or avoiding the select medication.

Antidepressants

Q48: Mirtazapine

eGFR 15-29	1	2	3	4	5
Select one<br>◯ Dose ADJUST<br>◯ Dose AVOID	◯	◯	◯	◯	◯
eGFR <15	1	2	3	4	5
Select one<br>◯ Dose ADJUST<br>◯ Dose AVOID	◯	◯	◯	◯	◯

Q49: Duloxetine

eGFR 15-29	1	2	3	4	5
Select one<br>◯ Dose ADJUST<br>◯ Dose AVOID	◯	◯	◯	◯	◯
eGFR <15	1	2	3	4	5
Select one<br>◯ Dose ADJUST<br>◯ Dose AVOID	◯	◯	◯	◯	◯

Q50: Venlafaxine

eGFR 15-29	1	2	3	4	5
Select one<br>◯ Dose ADJUST<br>◯ Dose AVOID	◯	◯	◯	◯	◯
eGFR <15	1	2	3	4	5
Select one<br>◯ Dose ADJUST<br>◯ Dose AVOID	◯	◯	◯	◯	◯

Q51: Escitalopram

eGFR 15-29	1	2	3	4	5
Select one<br>◯ Dose ADJUST<br>◯ Dose AVOID	◯	◯	◯	◯	◯
eGFR <15	1	2	3	4	5
Select one<br>◯ Dose ADJUST<br>◯ Dose AVOID	◯	◯	◯	◯	◯

For each medication in the survey, please **select** whether you would **dose adjust or avoid** based on the eGFR category (15-29 mL/min and <15 mL/min). Rate your level of agreement on a 5-point Likert scale (1= strongly disagree, 2 = disagree, 3 = neither agree or disagree, 4 = agree, 5 = strongly agree) on the importance of community pharmacists adjusting or avoiding the select medication.

Mineralocorticoid Receptor Antagonists

Q52: Eplerenone

eGFR 15-29	1	2	3	4	5
Select one<br>○ Dose ADJUST<br>○ Dose AVOID	○	○	○	○	○
eGFR <15	1	2	3	4	5
Select one<br>○ Dose ADJUST<br>○ Dose AVOID	○	○	○	○	○

Q53: Spironolactone

eGFR 15-29	1	2	3	4	5
Select one<br>○ Dose ADJUST<br>○ Dose AVOID	○	○	○	○	○
eGFR <15	1	2	3	4	5
Select one<br>○ Dose ADJUST<br>○ Dose AVOID	○	○	○	○	○

For each medication in the survey, please **select** whether you would **dose adjust or avoid** based on the eGFR category (>25 mL/min and <25 mL/min). Rate your level of agreement on a 5-point Likert scale (1= strongly disagree, 2 = disagree, 3 = neither agree or disagree, 4 = agree, 5 = strongly agree) on the importance of community pharmacists adjusting or avoiding the select medication.

Non-Steroidal Mineralocorticoid Receptor Antagonists

Q54: Finerenone

eGFR > 25	1	2	3	4	5
Select one<br>◯ Dose ADJUST<br>◯ Dose AVOID	◯	◯	◯	◯	◯
eGFR <25	1	2	3	4	5
Select one<br>◯ Dose ADJUST<br>◯ Dose AVOID	◯	◯	◯	◯	◯

For each medication in the survey, please **select** whether you would **dose adjust or avoid** based on the eGFR category (15-29 mL/min and <15 mL/min). Rate your level of agreement on a 5-point Likert scale (1= strongly disagree, 2 = disagree, 3 = neither agree or disagree, 4 = agree, 5 = strongly agree) on the importance of community pharmacists adjusting or avoiding the select medication.

CGRP Antagonists

Q55: Atogepant

eGFR 15-29	1	2	3	4	5
Select one<br>◯ Dose ADJUST<br>◯ Dose AVOID	◯	◯	◯	◯	◯
eGFR <15	1	2	3	4	5

Select one<br>◯ Dose ADJUST<br>◯ Dose AVOID	◯	◯	◯	◯	◯

Q56: Ubrogepant

eGFR 15-29	1	2	3	4	5
Select one<br>◯ Dose ADJUST<br>◯ Dose AVOID	◯	◯	◯	◯	◯
eGFR <15	1	2	3	4	5
Select one<br>◯ Dose ADJUST<br>◯ Dose AVOID	◯	◯	◯	◯	◯

For each medication in the survey, please **select** whether you would d**ose adjust or avoid** based on the eGFR category (15-29 mL/min and <15 mL/min). Rate your level of agreement on a 5-point Likert scale (1= strongly disagree, 2 = disagree, 3 = neither agree or disagree, 4 = agree, 5 = strongly agree) on the importance of community pharmacists adjusting or avoiding the select medication.

Other Drugs

Q57: Baclofen

eGFR 15-29	1	2	3	4	5
Select one<br>◯ Dose ADJUST<br>◯ Dose AVOID	◯	◯	◯	◯	◯
eGFR <15	1	2	3	4	5

Select one<br>◯ Dose ADJUST<br>◯ Dose AVOID	◯	◯	◯	◯	◯

Q58: Lithium

eGFR 15-29	1	2	3	4	5
Select one<br>◯ Dose ADJUST<br>◯ Dose AVOID	◯	◯	◯	◯	◯
eGFR <15	1	2	3	4	5
Select one<br>◯ Dose ADJUST<br>◯ Dose AVOID	◯	◯	◯	◯	◯

Q59: Metoclopramide

eGFR 15-29	1	2	3	4	5
Select one<br>◯ Dose ADJUST<br>◯ Dose AVOID	◯	◯	◯	◯	◯
eGFR <15	1	2	3	4	5
Select one<br>◯ Dose ADJUST<br>◯ Dose AVOID	◯	◯	◯	◯	◯

Q60: Digoxin

eGFR 15-29	1	2	3	4	5
Select one<br>◯ Dose ADJUST<br>◯ Dose AVOID	◯	◯	◯	◯	◯
eGFR <15	1	2	3	4	5
Select one<br>◯ Dose ADJUST<br>◯ Dose AVOID	◯	◯	◯	◯	◯

Q61: Methotrexate

eGFR 15-29	1	2	3	4	5
Select one<br>◯ Dose ADJUST<br>◯ Dose AVOID	◯	◯	◯	◯	◯
eGFR <15	1	2	3	4	5
Select one<br>◯ Dose ADJUST<br>◯ Dose AVOID	◯	◯	◯	◯	◯

Q62: Tizanidine

eGFR 15-29	1	2	3	4	5
Select one<br>◯ Dose ADJUST<br>◯ Dose AVOID	◯	◯	◯	◯	◯
eGFR <15	1	2	3	4	5
Select one<br>◯ Dose ADJUST<br>◯ Dose AVOID	◯	◯	◯	◯	◯

Q63: Risperidone

eGFR 15-29	1	2	3	4	5
Select one<br>◯ Dose ADJUST<br>◯ Dose AVOID	◯	◯	◯	◯	◯
eGFR <15	1	2	3	4	5
Select one<br>◯ Dose ADJUST<br>◯ Dose AVOID	◯	◯	◯	◯	◯

Q64: Abrocitinib

eGFR 15-29	1	2	3	4	5
Select one<br>◯ Dose ADJUST<br>◯ Dose AVOID	◯	◯	◯	◯	◯

eGFR <15	1	2	3	4	5
Select one<br>◯ Dose ADJUST<br>◯ Dose AVOID	◯	◯	◯	◯	◯

Q65: Varenicline

eGFR 15-29	1	2	3	4	5
Select one<br>◯ Dose ADJUST<br>◯ Dose AVOID	◯	◯	◯	◯	◯
eGFR <15	1	2	3	4	5
Select one<br>◯ Dose ADJUST<br>◯ Dose AVOID	◯	◯	◯	◯	◯

Q66: Sildenafil

eGFR 15-29	1	2	3	4	5
Select one<br>◯ Dose ADJUST<br>◯ Dose AVOID	◯	◯	◯	◯	◯
eGFR <15	1	2	3	4	5
Select one<br>◯ Dose ADJUST<br>◯ Dose AVOID	◯	◯	◯	◯	◯

Q67: Tadalafil

eGFR 15-29	1	2	3	4	5
Select one<br>○ Dose ADJUST<br>○ Dose AVOID	○	○	○	○	○
eGFR <15	1	2	3	4	5
Select one<br>○ Dose ADJUST<br>○ Dose AVOID	○	○	○	○	○

Q68: Sotalol

eGFR 15-29	1	2	3	4	5
Select one<br>○ Dose ADJUST<br>○ Dose AVOID	○	○	○	○	○
eGFR <15	1	2	3	4	5
Select one<br>○ Dose ADJUST<br>○ Dose AVOID	○	○	○	○	○

Q69: Amantadine

eGFR 15-29	1	2	3	4	5
Select one<br>◯ Dose ADJUST<br>◯ Dose AVOID	◯	◯	◯	◯	◯
eGFR <15	1	2	3	4	5
Select one<br>◯ Dose ADJUST<br>◯ Dose AVOID	◯	◯	◯	◯	◯

Q70: Memantine

eGFR 15-29	1	2	3	4	5
Select one<br>◯ Dose ADJUST<br>◯ Dose AVOID	◯	◯	◯	◯	◯
eGFR <15	1	2	3	4	5
Select one<br>◯ Dose ADJUST<br>◯ Dose AVOID	◯	◯	◯	◯	◯

Additional Questions

Q71: Please provide any general comments about the drugs listed in this survey.

Q72: Please include any medications that you would like considered for inclusion in the next round.

Consensus Building

Q73: Please provide your email for the survey system to send you your individual scores to support consensus building process for the next survey round. Please select 'Finish' to submit your responses.

Round 2: Modified Delphi Survey - Drugs to Adjust\Avoid in Non-Dialysis CKD eGFR below 30

Dear participant, this is **Round 2** of a Modified Delphi Survey process.
In Round 1, you were asked to select whether a medication should be dose adjusted or avoided for eGFR categories (15-29 mL/min and <15 mL/min) and to rate on a 5-point Likert scale (1= strongly disagree to 5 = strongly agree) your level of agreement on the importance of **COMMUNITY PHARMACISTS** adjusting or avoiding the select medication. We also asked you to list additional medications for consideration in Round 2.
In **Round 2,** for the additional medications you suggested from Round 1, you will be asked to select whether the medication should be dose adjusted or avoided based on eGFR categories (15-29 mL/min and <15 mL/min) and to rate on a 5-point Likert scale (1= strongly disagree to 5 = strongly agree) your level of agreement on the importance of **COMMUNITY PHARMACISTS** adjusting or avoiding the select medication. Importantly, you will be asked to select the **top 45 medications** you believe **COMMUNITY PHARMACISTS** should dose adjust or avoid in the community pharmacy setting.
The **aim of this Modified Delphi process** is to reach consensus on the **top medications** for **community pharmacists** to adjust or avoid in the community pharmacy setting. This medication list will be used to develop an electronic drug dosing tool for community pharmacists in individuals with eGFR of less than 30 mL/min.
Thank you for your participation.

Additional Medications for Consideration for Round 2

For each medication, please select whether you would dose adjust or avoid based on the eGFR category (15-29 mL/min and <15 mL/min). Rate your level of agreement on a 5-point Likert scale (1= strongly disagree, 2 = disagree, 3 = neither agree or disagree, 4 = agree, 5 = strongly agree) on the importance of **COMMUNITY PHARMACISTS** adjusting or avoiding the select medication.

Q1: Amoxicillin

eGFR 15-29	1	2	3	4	5
Select one<br>○ Dose ADJUST<br>○ Dose AVOID	○	○	○	○	○
eGFR <15	1	2	3	4	5

Select one<br>◯ Dose ADJUST<br>◯ Dose AVOID	◯	◯	◯	◯	◯

Q2: Amoxicillin / Clavulanic acid

eGFR 15-29	1	2	3	4	5
Select one<br>◯ Dose ADJUST<br>◯ Dose AVOID	◯	◯	◯	◯	◯
eGFR <15	1	2	3	4	5
Select one<br>◯ Dose ADJUST<br>◯ Dose AVOID	◯	◯	◯	◯	◯

Q3: Cephalexin

eGFR 15-29	1	2	3	4	5
Select one<br>◯ Dose ADJUST<br>◯ Dose AVOID	◯	◯	◯	◯	◯
eGFR <15	1	2	3	4	5
Select one<br>◯ Dose ADJUST<br>◯ Dose AVOID	◯	◯	◯	◯	◯

Q4: Clarithromycin

eGFR 15-29	1	2	3	4	5
Select one<br>◯ Dose ADJUST<br>◯ Dose AVOID	◯	◯	◯	◯	◯
eGFR <15	1	2	3	4	5
Select one<br>◯ Dose ADJUST<br>◯ Dose AVOID	◯	◯	◯	◯	◯

Q5: Bupropion

eGFR 15-29	1	2	3	4	5
Select one<br>◯ Dose ADJUST<br>◯ Dose AVOID	◯	◯	◯	◯	◯
eGFR <15	1	2	3	4	5
Select one<br>◯ Dose ADJUST<br>◯ Dose AVOID	◯	◯	◯	◯	◯

Medication List

Q6: Please select the top 45 medications you believe COMMUNITY PHARMACISTS should dose adjust or avoid in the community pharmacy setting.

- ☐ Meformin
- ☐ Glyburide
- ☐ Saxagliptin
- ☐ Sitagliptin
- ☐ Bezafibrate
- ☐ Fenofibrate
- ☐ Rosuvastatin
- ☐ Solifenacin
- ☐ Tolterodine
- ☐ Gabapentin
- ☐ Pregabalin
- ☐ Topiramate
- ☐ Cenobamate
- ☐ Allopurinol
- ☐ Colchicine
- ☐ Febuxostat
- ☐ Apixaban
- ☐ Dabigatran
- ☐ Edoxaban
- ☐ Rivaroxaban
- ☐ Dalteparin
- ☐ Tinzaparin
- ☐ Enoxaparin
- ☐ Acyclovir
- ☐ Famciclovir
- ☐ Valacyclovir
- ☐ Oseltamivir
- ☐ Paxlovid (Nirmatrelvir/ritonavir)
- ☐ Truvada (Emtricitabine / Tenofovir Disoproxil Fumarate)
- ☐ Amoxicillin
- ☐ Amoxicillin/Clavulanic Acid
- ☐ Cephalexin
- ☐ Clarithromycin
- ☐ Sulfamethoxazole / Trimethoprim
- ☐ Ciprofloxacin
- ☐ Levofloxacin
- ☐ Norfloxacin
- ☐ Nitrofurantoin
- ☐ Fluconazole
- ☐ Codeine
- ☐ Morphine
- ☐ Tramadol
- ☐ NSAIDs
- ☐ Ranitidine
- ☐ Famotidine

- [ ] Mirtazapine
- [ ] Duloxetine
- [ ] Bupropion
- [ ] Venlafaxine
- [ ] Escitalopram
- [ ] Eplerenone
- [ ] Spironolactone
- [ ] Finerenone
- [ ] Atogepant
- [ ] Ubrogepant
- [ ] Baclofen
- [ ] Lithium
- [ ] Metoclopramide
- [ ] Digoxin
- [ ] Methotrexate
- [ ] Tizanidine
- [ ] Risperidone
- [ ] Abrocitinib
- [ ] Varenicline
- [ ] Sildenafil
- [ ] Tadalafil
- [ ] Sotalol
- [ ] Amantadine
- [ ] Memantine
- [ ]

Q7: Do you have any comments you would like to share?

Q8: Please provide your email for the survey system to send you your individual scores to support consensus building process for the next survey round. Please select **'Finish'** to submit your responses.

Round 3: Modified Delphi Survey - Drugs to Adjust\Avoid in Non-Dialysis CKD eGFR below 30

Dear participant, this is **ROUND 3** of the modified Delphi process.
In Round 1 and Round 2, you were asked to select whether a medication should be dose adjusted or avoided for eGFR categories (15-29 mL/min and <15 mL/min) and to rate on a 5-point Likert scale (1= strongly disagree to 5 = strongly agree) your level of agreement on the importance of COMMUNITY PHARMACISTS adjusting or avoiding the select medication. Also, in Round 2, you were asked to select the **top 45 medications** you believe **community pharmacists** should dose adjust or avoid in the community pharmacy setting in individuals with an eGFR <30 mL/min.
For Round 3, you will first be asked to narrow your selection to the top 40 medications you believe community pharmacists should dose adjust or avoid in the community pharmacy setting for individuals with an eGFR <30 mL/min. You may wish to consider frequency of medication use and risk of potential medication harm.
Also for **Round 3**, we will again ask you to **select** whether a medication should be **dose adjusted or avoided** for eGFR categories (15-29 mL/min and <15 mL/min) and to rate on a 5-point Likert scale (1= strongly disagree to 5 = strongly agree) your level of agreement on the importance of **community pharmacists** adjusting or avoiding the select medication.
The **aim of this Modified Delphi process** is to reach consensus on the **top medications** for **community pharmacists** to **adjust or avoid** in the community pharmacy setting in collaboration with another prescriber if one is available. This medication list will be used to develop an electronic drug dosing tool for community pharmacists in individuals with **eGFR less than 30 mL/min**.
Thank you for your participation.

Medication List

Q1: Please select the top 40 medications you believe COMMUNITY PHARMACISTS should dose adjust or avoid in the community pharmacy setting for individuals with an eGFR < 30 mL/min.

- ☐ Meformin
- ☐ Glyburide
- ☐ Sitagliptin
- ☐ Bezafibrate
- ☐ Fenofibrate
- ☐ Tolterodine
- ☐ Gabapentin
- ☐ Pregabalin
- ☐ Topiramate
- ☐ Allopurinol
- ☐ Colchicine
- ☐ Febuxostat
- ☐ Apixaban (Atrial Fibrillation)
- ☐ Dabigatran (Atrial Fibrillation)
- ☐ Edoxaban (Atrial Fibrillation)
- ☐ Rivaroxaban (Atrial Fibrillation)

- [ ] Dalteparin (VTE Treatment)
- [ ] Enoxaparin (VTE Treatment and Prophylaxis)
- [ ] Acyclovir
- [ ] Famciclovir
- [ ] Valacyclovir
- [ ] Oseltamivir
- [ ] Paxlovid (Nirmatrelvir/ritonavir)
- [ ] Truvada (Emtricitabine / Tenofovir Disoproxil Fumarate)
- [ ] Amoxicillin/Clavulanic Acid
- [ ] Clarithromycin
- [ ] Sulfamethoxazole / Trimethoprim
- [ ] Ciprofloxacin
- [ ] Levofloxacin
- [ ] Norfloxacin
- [ ] Nitrofurantoin
- [ ] Fluconazole
- [ ] Codeine
- [ ] Morphine
- [ ] Tramadol
- [ ] NSAIDs
- [ ] Duloxetine
- [ ] Bupropion
- [ ] Eplerenone
- [ ] Finerenone
- [ ] Baclofen
- [ ] Lithium
- [ ] Digoxin (Maintenance)
- [ ] Methotrexate
- [ ] Varenicline
- [ ] Sotalol
- [ ] Amantadine

For each medication in the survey, please select whether you would dose adjust or avoid basedon the eGFR category (15-29 mL/min and <15 mL/min). Rate your level of agreement on a 5-pointLikert scale (1= strongly disagree, 2 = disagree, 3 = neither agree or disagree, 4 = agree, 5 =strongly agree) on the importance of community pharmacists adjusting or avoiding the selectmedication.

Antihyperglycemics

Q2: Metformin

eGFR 15-29	1	2	3	4	5
Select one<br>○ Dose ADJUST<br>○ Dose AVOID	○	○	○	○	○
eGFR <15	1	2	3	4	5
Select one<br>○ Dose ADJUST<br>○ Dose AVOID	○	○	○	○	○

Q3: Glyburide

eGFR 15-29	1	2	3	4	5
Select one<br>○ Dose ADJUST<br>○ Dose AVOID	○	○	○	○	○
eGFR <15	1	2	3	4	5
Select one<br>○ Dose ADJUST<br>○ Dose AVOID	○	○	○	○	○

Q4: Sitagliptin

eGFR 15-29	1	2	3	4	5
Select one<br>○ Dose ADJUST<br>○ Dose AVOID	○	○	○	○	○

eGFR <15	1	2	3	4	5
Select one<br>◯ Dose ADJUST<br>◯ Dose AVOID	◯	◯	◯	◯	◯

For each medication in the survey, please select whether you would dose adjust or avoid basedon the eGFR category (15-29 mL/min and <15 mL/min). Rate your level of agreement on a 5-pointLikert scale (1= strongly disagree, 2 = disagree, 3 = neither agree or disagree, 4 = agree, 5 =strongly agree) on the importance of community pharmacists adjusting or avoiding the selectmedication.

Lipid Lowering Agents

Q5: Bezafibrate

eGFR 15-29	1	2	3	4	5
Select one<br>◯ Dose ADJUST<br>◯ Dose AVOID	◯	◯	◯	◯	◯
eGFR <15	1	2	3	4	5
Select one<br>◯ Dose ADJUST<br>◯ Dose AVOID	◯	◯	◯	◯	◯

Q6: Fenofibrate

eGFR 15-29	1	2	3	4	5
Select one<br>◯ Dose ADJUST<br>◯ Dose AVOID	◯	◯	◯	◯	◯

eGFR <15	1	2	3	4	5
Select one<br>◯ Dose ADJUST<br>◯ Dose AVOID	◯	◯	◯	◯	◯

For each medication in the survey, please select whether you would dose adjust or avoid basedon the eGFR category (15-29 mL/min and <15 mL/min). Rate your level of agreement on a 5-pointLikert scale (1= strongly disagree, 2 = disagree, 3 = neither agree or disagree, 4 = agree, 5 =strongly agree) on the importance of community pharmacists adjusting or avoiding the selectmedication.

Antimuscarinics

Q7: Tolterodine

eGFR 15-29	1	2	3	4	5
Select one<br>◯ Dose ADJUST<br>◯ Dose AVOID	◯	◯	◯	◯	◯
eGFR <15	1	2	3	4	5
Select one<br>◯ Dose ADJUST<br>◯ Dose AVOID	◯	◯	◯	◯	◯

For each medication in the survey, please select whether you would dose adjust or avoid basedon the eGFR category (15-29 mL/min and <15 mL/min). Rate your level of agreement on a 5-pointLikert scale (1= strongly disagree, 2 = disagree, 3 = neither agree or disagree, 4 = agree, 5 =strongly agree) on the importance of community pharmacists adjusting or avoiding the selectmedication.

Anticonvulsants

Q8: Gabapentin

eGFR 15-29	1	2	3	4	5
Select one<br>◯ Dose ADJUST<br>◯ Dose AVOID	◯	◯	◯	◯	◯
eGFR <15	1	2	3	4	5
Select one<br>◯ Dose ADJUST<br>◯ Dose AVOID	◯	◯	◯	◯	◯

Q9: Pregabalin

eGFR 15-29	1	2	3	4	5
Select one<br>◯ Dose ADJUST<br>◯ Dose AVOID	◯	◯	◯	◯	◯
eGFR <15	1	2	3	4	5
Select one<br>◯ Dose ADJUST<br>◯ Dose AVOID	◯	◯	◯	◯	◯

Q10: Topiramate

eGFR 15-29	1	2	3	4	5
Select one<br>◯ Dose ADJUST<br>◯ Dose AVOID	◯	◯	◯	◯	◯
eGFR <15	1	2	3	4	5
Select one<br>◯ Dose ADJUST<br>◯ Dose AVOID	◯	◯	◯	◯	◯

For each medication in the survey, please select whether you would dose adjust or avoid basedon the eGFR category (15-29 mL/min and <15 mL/min). Rate your level of agreement on a 5-pointLikert scale (1= strongly disagree, 2 = disagree, 3 = neither agree or disagree, 4 = agree, 5 =strongly agree) on the importance of community pharmacists adjusting or avoiding the selectmedication.

Urate Lowering Agents

Q11: Allopurinol

eGFR 15-29	1	2	3	4	5
Select one<br>◯ Dose ADJUST<br>◯ Dose AVOID	◯	◯	◯	◯	◯
eGFR <15	1	2	3	4	5
Select one<br>◯ Dose ADJUST<br>◯ Dose AVOID	◯	◯	◯	◯	◯

Q12: Colchicine

eGFR 15-29	1	2	3	4	5
Select one<br>◯ Dose ADJUST<br>◯ Dose AVOID	◯	◯	◯	◯	◯
eGFR <15	1	2	3	4	5
Select one<br>◯ Dose ADJUST<br>◯ Dose AVOID	◯	◯	◯	◯	◯

Q13: Febuxostat

eGFR 15-29	1	2	3	4	5
Select one<br>◯ Dose ADJUST<br>◯ Dose AVOID	◯	◯	◯	◯	◯
eGFR <15	1	2	3	4	5
Select one<br>◯ Dose ADJUST<br>◯ Dose AVOID	◯	◯	◯	◯	◯

For each medication in the survey, please select whether you would dose adjust or avoid basedon the eGFR category (15-29 mL/min and <15 mL/min). Rate your level of agreement on a 5-pointLikert scale (1= strongly disagree, 2 = disagree, 3 = neither agree or disagree, 4 = agree, 5 =strongly agree) on the importance of community pharmacists adjusting or avoiding the selectmedication.

Anticoagulants

Q14: Apixaban (Atrial Fibrillation)

eGFR 15-29	1	2	3	4	5
Select one<br>◯ Dose ADJUST<br>◯ Dose AVOID	◯	◯	◯	◯	◯
eGFR <15	1	2	3	4	5
Select one<br>◯ Dose ADJUST<br>◯ Dose AVOID	◯	◯	◯	◯	◯

Q15: Dabigatran (Atrial Fibrillation)

eGFR 15-29	1	2	3	4	5
Select one<br>◯ Dose ADJUST<br>◯ Dose AVOID	◯	◯	◯	◯	◯
eGFR <15	1	2	3	4	5
Select one<br>◯ Dose ADJUST<br>◯ Dose AVOID	◯	◯	◯	◯	◯

Q16: Edoxaban (Atrial Fibrillation)

eGFR 15-29	1	2	3	4	5
Select one<br>◯ Dose ADJUST<br>◯ Dose AVOID	◯	◯	◯	◯	◯
eGFR <15	1	2	3	4	5
Select one<br>◯ Dose ADJUST<br>◯ Dose AVOID	◯	◯	◯	◯	◯

Q17: Rivaroxaban (Atrial Fibrillation)

eGFR 15-29	1	2	3	4	5
Select one<br>◯ Dose ADJUST<br>◯ Dose AVOID	◯	◯	◯	◯	◯
eGFR <15	1	2	3	4	5
Select one<br>◯ Dose ADJUST<br>◯ Dose AVOID	◯	◯	◯	◯	◯

For each medication in the survey, please select whether you would dose adjust or avoid basedon the eGFR category (15-29 mL/min and <15 mL/min). Rate your level of agreement on a 5-pointLikert scale (1= strongly disagree, 2 = disagree, 3 = neither agree or disagree, 4 = agree, 5 =strongly agree) on the importance of community pharmacists adjusting or avoiding the selectmedication.

Low Molecular Weight Heparins

Q18: Dalteparin (VTE Treatment)

eGFR 15-29	1	2	3	4	5
Select one<br>◯ Dose ADJUST<br>◯ Dose AVOID	◯	◯	◯	◯	◯
eGFR <15	1	2	3	4	5
Select one<br>◯ Dose ADJUST<br>◯ Dose AVOID	◯	◯	◯	◯	◯

Q19: Enoxaparin (VTE Treatment and Prophylaxis)

eGFR 15-29	1	2	3	4	5
Select one<br>◯ Dose ADJUST<br>◯ Dose AVOID	◯	◯	◯	◯	◯
eGFR <15	1	2	3	4	5
Select one<br>◯ Dose ADJUST<br>◯ Dose AVOID	◯	◯	◯	◯	◯

For each medication in the survey, please select whether you would dose adjust or avoid basedon the eGFR category (15-29 mL/min and <15 mL/min). Rate your level of agreement on a 5-pointLikert scale (1= strongly disagree, 2 = disagree, 3 = neither agree or disagree, 4 = agree, 5 =strongly agree) on the importance of community pharmacists adjusting or avoiding the selectmedication.

Antivirals

Q20: Acyclovir

eGFR 15-29	1	2	3	4	5
Select one<br>◯ Dose ADJUST<br>◯ Dose AVOID	◯	◯	◯	◯	◯
eGFR <15	1	2	3	4	5
Select one<br>◯ Dose ADJUST<br>◯ Dose AVOID	◯	◯	◯	◯	◯

Q21: Famciclovir

eGFR 15-29	1	2	3	4	5
Select one<br>◯ Dose ADJUST<br>◯ Dose AVOID	◯	◯	◯	◯	◯
eGFR <15	1	2	3	4	5
Select one<br>◯ Dose ADJUST<br>◯ Dose AVOID	◯	◯	◯	◯	◯

Q22: Valacyclovir

eGFR 15-29	1	2	3	4	5
Select one<br>◯ Dose ADJUST<br>◯ Dose AVOID	◯	◯	◯	◯	◯
eGFR <15	1	2	3	4	5
Select one<br>◯ Dose ADJUST<br>◯ Dose AVOID	◯	◯	◯	◯	◯

Q23: Oseltamivir

eGFR 15-29	1	2	3	4	5
Select one<br>◯ Dose ADJUST<br>◯ Dose AVOID	◯	◯	◯	◯	◯
eGFR <15	1	2	3	4	5
Select one<br>◯ Dose ADJUST<br>◯ Dose AVOID	◯	◯	◯	◯	◯

Q24: Paxlovid (nirmatrelvir/ritonavir)

eGFR 15-29	1	2	3	4	5
Select one<br>◯ Dose ADJUST<br>◯ Dose AVOID	◯	◯	◯	◯	◯
eGFR <15	1	2	3	4	5
Select one<br>◯ Dose ADJUST<br>◯ Dose AVOID	◯	◯	◯	◯	◯

Q25: Truvada (emtricitabine / tenofovir disoproxil fumarate)

eGFR 15-29	1	2	3	4	5
Select one<br>◯ Dose ADJUST<br>◯ Dose AVOID	◯	◯	◯	◯	◯
eGFR <15	1	2	3	4	5
Select one<br>◯ Dose ADJUST<br>◯ Dose AVOID	◯	◯	◯	◯	◯

For each medication in the survey, please select whether you would dose adjust or avoid basedon the eGFR category (15-29 mL/min and <15 mL/min). Rate your level of agreement on a 5-pointLikert scale (1= strongly disagree, 2 = disagree, 3 = neither agree or disagree, 4 = agree, 5 =strongly agree) on the importance of community pharmacists adjusting or avoiding the selectmedication.

Antimicrobials

Q26: Amoxicillin / Clavulanic acid

eGFR 15-29	1	2	3	4	5
Select one<br>◯ Dose ADJUST<br>◯ Dose AVOID	◯	◯	◯	◯	◯
eGFR <15	1	2	3	4	5
Select one<br>◯ Dose ADJUST<br>◯ Dose AVOID	◯	◯	◯	◯	◯

Q27: Clarithromycin

eGFR 15-29	1	2	3	4	5
Select one<br>◯ Dose ADJUST<br>◯ Dose AVOID	◯	◯	◯	◯	◯
eGFR <15	1	2	3	4	5
Select one<br>◯ Dose ADJUST<br>◯ Dose AVOID	◯	◯	◯	◯	◯

Q28: Sulfamethoxazole/Trimethoprim

eGFR 15-29	1	2	3	4	5
Select one<br>◯ Dose ADJUST<br>◯ Dose AVOID	◯	◯	◯	◯	◯
eGFR <15	1	2	3	4	5
Select one<br>◯ Dose ADJUST<br>◯ Dose AVOID	◯	◯	◯	◯	◯

Q29: Ciprofloxacin

eGFR 15-29	1	2	3	4	5
Select one<br>◯ Dose ADJUST<br>◯ Dose AVOID	◯	◯	◯	◯	◯
eGFR <15	1	2	3	4	5
Select one<br>◯ Dose ADJUST<br>◯ Dose AVOID	◯	◯	◯	◯	◯

Q30: Levofloxacin

eGFR 15-29	1	2	3	4	5
Select one<br>◯ Dose ADJUST<br>◯ Dose AVOID	◯	◯	◯	◯	◯

eGFR <15	1	2	3	4	5
Select one<br>○ Dose ADJUST<br>○ Dose AVOID	○	○	○	○	○

Q31: Norfloxacin

eGFR 15-29	1	2	3	4	5
Select one<br>○ Dose ADJUST<br>○ Dose AVOID	○	○	○	○	○
eGFR <15	1	2	3	4	5
Select one<br>○ Dose ADJUST<br>○ Dose AVOID	○	○	○	○	○

Q32: Nitrofurantoin

eGFR 15-29	1	2	3	4	5
Select one<br>○ Dose ADJUST<br>○ Dose AVOID	○	○	○	○	○
eGFR <15	1	2	3	4	5
Select one<br>○ Dose ADJUST<br>○ Dose AVOID	○	○	○	○	○

For each medication in the survey, please select whether you would dose adjust or avoid basedon the eGFR category (15-29 mL/min and <15 mL/min). Rate your level of agreement on a 5-pointLikert scale (1= strongly disagree, 2 = disagree, 3 = neither agree or disagree, 4 = agree, 5 =strongly agree) on the importance of community pharmacists adjusting or avoiding the selectmedication.

Antifungals

Q33: Fluconazole

eGFR 15-29	1	2	3	4	5
Select one<br>◯ Dose ADJUST<br>◯ Dose AVOID	◯	◯	◯	◯	◯
eGFR <15	1	2	3	4	5
Select one<br>◯ Dose ADJUST<br>◯ Dose AVOID	◯	◯	◯	◯	◯

For each medication in the survey, please select whether you would dose adjust or avoid based on the eGFR category (15-29 mL/min and <15 mL/min). Rate your level of agreement on a 5-point Likert scale (1= strongly disagree, 2 = disagree, 3 = neither agree or disagree, 4 = agree, 5 = strongly agree) on the importance of community pharmacists adjusting or avoiding the select medication.

Analgesics and Opioids

Q34: Codeine

eGFR 15-29	1	2	3	4	5
Select one<br>◯ Dose ADJUST<br>◯ Dose AVOID	◯	◯	◯	◯	◯
eGFR <15	1	2	3	4	5

Select one<br>◯ Dose ADJUST<br>◯ Dose AVOID	◯	◯	◯	◯	◯

Q35: Morphine

eGFR 15-29	1	2	3	4	5
Select one<br>◯ Dose ADJUST<br>◯ Dose AVOID	◯	◯	◯	◯	◯
eGFR <15	1	2	3	4	5
Select one<br>◯ Dose ADJUST<br>◯ Dose AVOID	◯	◯	◯	◯	◯

Q36: Tramadol

eGFR 15-29	1	2	3	4	5
Select one<br>◯ Dose ADJUST<br>◯ Dose AVOID	◯	◯	◯	◯	◯
eGFR <15	1	2	3	4	5
Select one<br>◯ Dose ADJUST<br>◯ Dose AVOID	◯	◯	◯	◯	◯

Q37: NSAIDs

eGFR 15-29	1	2	3	4	5
Select one<br>◯ Dose ADJUST<br>◯ Dose AVOID	◯	◯	◯	◯	◯
eGFR <15	1	2	3	4	5
Select one<br>◯ Dose ADJUST<br>◯ Dose AVOID	◯	◯	◯	◯	◯

For each medication in the survey, please select whether you would dose adjust or avoid basedon the eGFR category (15-29 mL/min and <15 mL/min). Rate your level of agreement on a 5-pointLikert scale (1= strongly disagree, 2 = disagree, 3 = neither agree or disagree, 4 = agree, 5 =strongly agree) on the importance of community pharmacists adjusting or avoiding the selectmedication.

Antidepressants

Q38: Bupropion

eGFR 15-29	1	2	3	4	5
Select one<br>◯ Dose ADJUST<br>◯ Dose AVOID	◯	◯	◯	◯	◯
eGFR <15	1	2	3	4	5
Select one<br>◯ Dose ADJUST<br>◯ Dose AVOID	◯	◯	◯	◯	◯

Q39: Duloxetine

eGFR 15-29	1	2	3	4	5
Select one<br>◯ Dose ADJUST<br>◯ Dose AVOID	◯	◯	◯	◯	◯
eGFR <15	1	2	3	4	5
Select one<br>◯ Dose ADJUST<br>◯ Dose AVOID	◯	◯	◯	◯	◯

For each medication in the survey, please select whether you would dose adjust or avoid basedon the eGFR category (15-29 mL/min and <15 mL/min). Rate your level of agreement on a 5-pointLikert scale (1= strongly disagree, 2 = disagree, 3 = neither agree or disagree, 4 = agree, 5 =strongly agree) on the importance of community pharmacists adjusting or avoiding the selectmedication.

Mineralocorticoid Receptor Antagonist

Q40: Eplerenone

eGFR 15-29	1	2	3	4	5
Select one<br>◯ Dose ADJUST<br>◯ Dose AVOID	◯	◯	◯	◯	◯
eGFR <15	1	2	3	4	5
Select one<br>◯ Dose ADJUST<br>◯ Dose AVOID	◯	◯	◯	◯	◯

For each medication in the survey, please select whether you would dose adjust or avoid basedon the eGFR category (15-29 mL/min and <15 mL/min). Rate your level of agreement on a 5-pointLikert scale (1= strongly disagree, 2 = disagree, 3 = neither agree or disagree, 4 = agree, 5 =strongly agree) on the importance of community pharmacists adjusting or avoiding the selectmedication.

Non-Steroidal Mineralocorticoid Receptor Antagonists

Q41: Finerenone

eGFR 15-29	1	2	3	4	5
Select one<br>○ Dose ADJUST<br>○ Dose AVOID	○	○	○	○	○
eGFR <15	1	2	3	4	5
Select one<br>○ Dose ADJUST<br>○ Dose AVOID	○	○	○	○	○

For each medication in the survey, please select whether you would dose adjust or avoid basedon the eGFR category (15-29 mL/min and <15 mL/min). Rate your level of agreement on a 5-pointLikert scale (1= strongly disagree, 2 = disagree, 3 = neither agree or disagree, 4 = agree, 5 =strongly agree) on the importance of community pharmacists adjusting or avoiding the selectmedication.

Other

Q42: Baclofen

eGFR 15-29	1	2	3	4	5
Select one<br>○ Dose ADJUST<br>○ Dose AVOID	○	○	○	○	○
eGFR <15	1	2	3	4	5

Select one<br>◯ Dose ADJUST<br>◯ Dose AVOID	◯	◯	◯	◯	◯

Q43: Lithium

eGFR 15-29	1	2	3	4	5
Select one<br>◯ Dose ADJUST<br>◯ Dose AVOID	◯	◯	◯	◯	◯
eGFR <15	1	2	3	4	5
Select one<br>◯ Dose ADJUST<br>◯ Dose AVOID	◯	◯	◯	◯	◯

Q44: Digoxin (Maintenance)

eGFR 15-29	1	2	3	4	5
Select one<br>◯ Dose ADJUST<br>◯ Dose AVOID	◯	◯	◯	◯	◯
eGFR <15	1	2	3	4	5
Select one<br>◯ Dose ADJUST<br>◯ Dose AVOID	◯	◯	◯	◯	◯

Q45: Methotrexate

eGFR 15-29	1	2	3	4	5
Select one<br>◯ Dose ADJUST<br>◯ Dose AVOID	◯	◯	◯	◯	◯
eGFR <15	1	2	3	4	5
Select one<br>◯ Dose ADJUST<br>◯ Dose AVOID	◯	◯	◯	◯	◯

Q46: Varenicline

eGFR 15-29	1	2	3	4	5
Select one<br>◯ Dose ADJUST<br>◯ Dose AVOID	◯	◯	◯	◯	◯
eGFR <15	1	2	3	4	5
Select one<br>◯ Dose ADJUST<br>◯ Dose AVOID	◯	◯	◯	◯	◯

Q47: Sotalol

eGFR 15-29	1	2	3	4	5
Select one<br>◯ Dose ADJUST<br>◯ Dose AVOID	◯	◯	◯	◯	◯
eGFR <15	1	2	3	4	5
Select one<br>◯ Dose ADJUST<br>◯ Dose AVOID	◯	◯	◯	◯	◯

Q48: Amantadine

eGFR 15-29	1	2	3	4	5
Select one<br>◯ Dose ADJUST<br>◯ Dose AVOID	◯	◯	◯	◯	◯
eGFR <15	1	2	3	4	5
Select one<br>◯ Dose ADJUST<br>◯ Dose AVOID	◯	◯	◯	◯	◯

Other

Q49: Of the medications you were asked to rank, were there any medications you would not dose adjust or avoid?

Final Questions

Q50: Do you have any comments you would like to share?

Q51: Please provide your email for the survey system to send you your individual scores to support consensus building process for the next survey round. Please select **'Finish'** to submit your responses.
