# Supplementary material for: Medications for community pharmacists to dose adjust or avoid to enhance prescribing safety in individuals with advanced chronic kidney disease: a scoping review and modified Delphi
Source: BMC Nephrol. 2024 Oct 29;25:386. doi: 10.1186/s12882-024-03829-y (PMC11523796; doi:10.1186/s12882-024-03829-y)
Supplement: Supplementary file 4 — Additional file 4: Medications presented to Modified Delphi. [file 12882_2024_3829_MOESM4_ESM.pdf]

Additional File 4. Medications Presented to Modified Delphi[t]

Antihyperglycemics	Antivirals	Mineralocorticoid Receptor Antagonists
Metformin	Acyclovir	Eplerenone *
Glyburide	Famciclovir	Spironolactone
Saxagliptin	Valacyclovir	**CGRP Antagonist**
Sitagliptin	Oseltamivir	Atogepant *
**Lipid Lowering Agents**	Paxlovid *	Ubrogepant *
Bezafibrate	Truvada *	**Antidepressants**
Fenofibrate	**Antimicrobials**	Bupropion**
Rosuvastatin	Amoxicillin**	Duloxetine
**Antimuscarinic Agents**	Amoxicillin / Clavulanic Acid**	Venlafaxine
Solifenacin	Cephalexin**	Escitalopram
Tolterodine	Clarithromycin**	Mirtazapine
**Anticonvulsants**	Sulfamethoxazole / Trimethoprim	**Other Drugs**
Gabapentin	Ciprofloxacin	Baclofen
Pregabalin	Levofloxacin	Lithium
Topiramate	Norfloxacin *	Metoclopramide
Cenobamate*	Nitrofurantoin	Digoxin
**Urate Lowering Agents**	**Antifungals**	Methotrexate *
Allopurinol	Fluconazole	Tizanidine *
Colchicine	**Analgesics / Opioids**	Risperidone
Febuxostat	NSAIDs	Abrocitinib *
**Anticoagulants**	Codeine	Varenicline
Apixaban	Morphine	Sildenafil
Dabigatran	Tramadol	Tadalafil
Edoxaban	**H2RA Antagonists**	Sotalol
Rivaroxaban	Ranitidine	Amantadine
Dalteparin	Famotidine	Memantine
Tinzaparin	**Non-Steroidal Mineralocorticoid Receptor Antagonist**	Proton Pump Inhibitors (PPI) *
Enoxaparin	Finerenone *	

Paxlovid = Nirmatrelvir / Ritonavir; Truvada = Emtricitabine / Tenofovir Disoproxil Fumarate

[t]includes 64 medications identified from the scoping review

* Twelve unique medications identified only from the scoping review. Note: PPIs broader drug class was not included in modified Delphi as will be incorporated into translation efforts

** Five additional medications added from participants in round 1 for round 2
