# Supplementary material for: Medications for community pharmacists to dose adjust or avoid to enhance prescribing safety in individuals with advanced chronic kidney disease: a scoping review and modified Delphi
Source: BMC Nephrol. 2024 Oct 29;25:386. doi: 10.1186/s12882-024-03829-y (PMC11523796; doi:10.1186/s12882-024-03829-y)
Supplement: Supplementary file 5 — Additional file 5: Scoping Review Data Extraction of 12 Unique Medications. [file 12882_2024_3829_MOESM5_ESM.pdf]

Additional File 5. Scoping Review Data Extraction for Twelve Unique Medications

Author, Year, Country, and Aim	Study Design, Participants, Setting	Methodology and Analysis	Relevant Study Findings, Drug(s) to Avoid or Adjust
Petruccelli et al., 2022, Brazil (1)<br><br>To assess kidney function for individuals taking PrEP (emtricitabine/TDF) over 48 weeks.	**Study Design**: Retrospective analysis of data collected in PrEP Brazil Study.<br><br>**Participants:** N = 392 Age ≥ 18 years, eGFR ≥ 60 mL/min/1.73m^2.<br>**Setting:** Brazilian Centers for HIV prevention.	**Methodology**: eGFR measurement assessed at 4,12,24,26 and 48 weeks in individuals administered PrEP. Participants stratified into 2 groups: eGFR ≤ 90 mL/min/1.73m^2 (n=162) and eGFR > 90 mL/min/1.73m^2 (n=230). Adherence assessed by TDF concentration in dried blood spots via mass spectrometry at weeks 4 and 48.<br><br>**Analysis:** ANOVA and post hoc analysis by Bonferroni were used to compare eGFR between weeks. Linear regression was performed for eGFR, TDF concentration and eGFR variation.	**Findings:** At 4 weeks, 90 participants (23%) presented with reductions in eGFR > 10 mL/min/1.73m^2 as compared to baseline and some as large as 59 mL/min/1.73m^2. There was a significant reduction in eGFR compared to baseline (-3.46 ± 13.29, p < 0.001). A negative relationship was demonstrated between TDF blood levels and eGFR at 4 weeks (r = -0.005, p < 0.01) and at 48 weeks (r = -0.006, p < 0.01).<br><br>**Drugs to Avoid or Adjust:** Emtricitabine/tenofovir disoproxil fumarate
Schaefer et al., 2022, Multi-Country (2)<br><br>To review published literature on kidney toxicity among TDF-based oral PrEP users and to conduct a pooled and an IPDMA on kidney function for PrEP users.	**Study Design:** Systematic review and meta-analysis of RCTs or cohort studies that reported on graded kidney-related adverse events among PrEP users.<br><br>**Participants:** N = 13,523 (pooled meta-analysis) N= 18 676 (global dataset of PrEP users screened for PrEP initiation across 15 countries).	**Methodology:** PubMed search conducted June 30, 2021. Summary data extracted and meta-analyses conducted with random-effects models to estimate relative risks of grade 1 and higher adverse events (measured by increased SCr or decline in CrCL or eGFR. CrCL stages were defined as > 90, 60-89 and < 60 mL/min).<br><br>**Analysis:** Assessed graded kidney-related adverse events. Primary outcome measure after PrEP initiation was a deterioration to clinically significant CrCl < 60 mL/min (referred to as a significant decline).	**Findings:** 62 unique records were identified which included 17 articles reporting on 11 RCTs with 13,523 participants in the pooled meta-analysis. PrEP was associated with increased risk of grade 1 or higher kidney adverse events (pooled OR 1.49; 95% CI, 1.22-1.81, I^2 = 25%). In the IPDMA, longitudinal analyses included 14 368 PrEP users and 349 (2.43%) individuals had a CrCl decline to < 60 mL/min with higher risks associated with increasing age and baseline CrCl of 60-89 mL/min (aHR 8.49%, 95% CI, 6.44-11.20) and CrCL < 60 mL/min (aHR 20.83%, 95% CI, 12.83-33.82).<br><br>**Drugs to Avoid or Adjust:** Tenofovir disoproxil fumarate based oral PrEP
Toussi et al., 2022, USA (3)<br><br>To evaluate the effects of renal	**Study Design:** Phase I, non-randomized, open label PK study.	**Methodology:** Single oral dose 100 mg nirmatrelvir and 100 mg ritonavir administered on Day 1 with additional doses of ritonavir	**Findings:** Systemic nirmatrelvir exposure increased with decreased renal function. The adjusted geometric mean ratio $AUC_{0\rightarrow inf}$ was 124% (mild),

impairment on the pharmacokinetics, safety and tolerability of nirmatrelvir/ritonavir.	**Participants:**<br>N = 34 (n=10 normal renal function; n=8 each for mild [eGFR 60 - < 90 mL/min], moderate [30 - < 60 mL/min] and severe [< 30 mL/min] renal impairment)<br>Age 18-75 years, BMI 17.5 – 40 kg/m^2 and total body weight of > 50 kg.	100 mg administered 12 and 24 hours after nirmatrelvir dosing.<br>**Analysis:** Primary PK parameters were Cmax, $AUC_{0\rightarrow inf}$, percentage of unchanged drug excreted in the urine over 48h, and CLr. Blood samples were collected before dosing and 0.5, 1, 2, 3, 4, 6, 8, 10, 12, 24, 36, and 48-hours following dose. Urine samples for additional PK analysis were collected in intervals of ≤24 hours and >24 to ≤48 hours; Safety and tolerability were accessed as a secondary outcome.	187% (moderate), and 304% (severe) compared to normal renal function. Ratios of Cmax for mild, moderate, and severe renal function were 129.8%, 138%, 148%, respectively compared to normal renal function. CL/F was significant correlated with eGFR. Mean CL/F values were 5.58 (mild), 3.60 (moderate) and 2.27 L/hour (severe) compared to 6.91 L/hour for normal renal function group. In normal renal function the half-life was 7.73 hours and increased to 13.37 hours in severe renal impairment. Treatment-related adverse events were mild in severity.<br><br>**Drugs to Avoid or Adjust:**<br>Nirmatrelvir/ritonavir
Chan et al., 2023, China (4)<br><br>To determine the safety profile and clinical/virological outcomes of nirmatrelvir/ritonavir at a modified dose in CKD.	**Study Design:**<br>Prospective, single-arm interventional study.<br><br>**Participants:**<br>N = 85<br>Adults with CKD (n= 10 eGFR > 60, n=10 eGFR 30-60, n=6 eGFR < 30 mL/min/1.73m^2 and n=59 dialysis) with COVID-19 infection and symptoms < 5 days.	**Methodology:** A modified dose was administered based on eGFR. Patients were followed for 30 days after treatment and PCR tests repeated at days 5, 15 30 and if symptoms.<br><br>**Analysis:** Primary outcomes included safety profile, adverse/serious adverse events and events leading to drug discontinuation.	**Findings:** 9.4% and 5.9% of patients experienced adverse and serious adverse events and these were comparable between eGFR groups < or > 30 mL/min/1.73m^2. The viral load significantly decreased on days 5,15 and 30 (p< 0.001) with reductions consistent in subgroups with eGFR < 30 mL/min/1.73m^2.<br><br>**Drugs to Avoid or Adjust:**<br>Nirmatrelvir/ritonavir

Muanda et al., 2022, Canada (5)<br><br>To evaluate the 14-day risk of hospitalization due to nervous system and/or psychiatric disorders, hypoglycemia, or collagen related event in patients with advanced CKD prescribed a low vs high dose fluoroquinolone.	**Study Design:** Population based cohort study (8 health care databases as part of the Institute for Clinical Evaluation Sciences (January 1, 2008-March 17, 2020).<br><br>**Participants:** N = 11,917 Age 66 years or older with advanced CKD (eGFR <30 mL/min/1.73m^2 but not receiving dialysis).	**Methodology:** New prescription for a higher-dose fluoroquinolone (ciprofloxacin, 501-1000 mg/day; levofloxacin, 501-750 mg/day; or norfloxacin, 401-800 mg/day) versus a lower-dose fluoroquinolone (ciprofloxacin, 500 mg/day; levofloxacin, 250-500 mg/day; or norfloxacin, 400 mg/day). The dispense date served as the date of cohort entry (index date). Study restricted the cohort to patients with at least 1 SCr measurement in the period from 1 year to 7 days before the index date.<br><br>**Analysis:** Primary outcome was hospitalization due to adverse effects associated with fluroquinolones. Secondary outcomes included a hospital visit with sepsis, retinal detachment or other tendinopathies, all-cause hospitalization, all-cause mortality, and sudden cardiac death.	**Findings:** 5482 (46.0%) received a high-dose and 6435 (54.0%) received a low-dose of a fluoroquinolone. The primary composite outcome occurred in 1.2% treated with a higher-dose fluoroquinolone and 0.9% treated with a lower-dose fluoroquinolone (weighted risk ratio, 1.45; 95% CI, 1.01-2.08); weighted risk difference, 0.39%; 95% CI, 0.01%-0.76%). The risk of secondary outcome did not differ significantly between groups.<br><br>**Drugs to Avoid or Adjust:** Norfloxacin (Ciprofloxacin, Levofloxacin include)
Muanda et al., 2023, Canada (6)<br><br>To compare the 90-day risk of serious AEs among person with CKD who started low-dose MTX versus hydroxychloroquine and to compare the risk of serious adverse events among adults with CKD starting 2 distinct doses of methotrexate vs those starting hydroxychloroquine.	**Study Design:** Retrospective, population-based, new-user cohort (prescription for oral M or hydroxychloroquine) study using ICES (January 1, 2008 - July 31, 2021).<br><br>**Participants:** N = 6909, (n= 2900 MTX and n= 4009 hydroxychloroquine) Age 66 years and older with an eGFR < 60 mL/min/1.73m^2 (excluding patients	**Methodology:** The study used linked administrative health care databases. The prescription dispense date served as the date of cohort entry (index date). eGFR was before index was used. Two cohorts of MTX included those who started 5 mg - < 15 mg/week and 15-35 mg/week compared to hydroxychloroquine.<br><br>**Analysis:** The primary outcome was a composite of serious AE: 90-day hospital visits since starting study drug due to myelosuppression, sepsis, pneumotoxic effects, hepatotoxic effects. Subgroup analyses were conducted by eGFR category. RRs	**Findings:** The primary outcome occurred in 3.55% and 1.73% of patients that started low-dose MTX vs hydroxychloroquine, respectfully (RR 2.05; 95% CI, 1.42-2.96) RD 1.82%; 95% CI, 0.91-2.73%). In subgroup analysis, the risk increased significantly with lower eGFR (e.g., eGFR < 45 mL/min/1.73m^2, RR 2.79; 95% CI, 1.51-5.13). MTX users at 15-25 mg/week had a higher risk of the primary outcome compared to hydroxychloroquine.<br><br>**Drugs to Avoid or Adjust:** Methotrexate

	receiving dialysis or kidney transplant).	and RD were obtained using regression.	
Hu et al., China, 2023 (7)<br><br>To identify RCTs correlated with eplerenone for treating CKD and hyperkalemia among other renal outcomes.	**Study Design:** Meta-analysis of RCTs up to September 21, 2022.<br><br>**Participants:** N = 19 RCTs eplerenone with or without mild/moderate hypertension, or with or without ACEi/ARB compared to placebo N = 4501 adults with eGFR > 30 mL/min/1.73m^2, serum potassium (K+) ≤5 mmol/L 24 h prior to randomization, diagnosed with CKD.	**Methodology:** Articles were screened against inclusion and exclusion criteria and evaluated for risk of bias with use of eplerenone with or without ACEi/ARB in comparison to placebo or active control for at least 4 weeks.<br><br>**Analysis:** Primary endpoint was 24-hour proteinuria, urine ACR, eGFR, SCr, SBP, DBP, K+ and adverse events of hyperkalemia (K+ ≥5.5 and ≥6.0 mmol/L). The use of 95% CI, and MD were conducted on continuous variable.	**Findings:** Significant rises in K+ were reported with eplerenone versus placebo (MD 0.13; 95% CI, 0.07-0.18, p < 0.00001) and eplerenone versus thiazide (MD 0.18; 95% CI, 0.13-0.23, p< 0.00001). Significant risks of hyperkalemia were evident in eplerenone groups (K+ ≥ 5.5 mmol/L, RR 1.70; 95% CI, 1.35-2.13 and K+ ≥ 6.0 mmol/L, RR1.61; 95% CI, 1.06-2.44, p=0.02).<br><br>**Drugs to Avoid or Adjust:** Eplerenone
Sarafidis et al., Multi-Country, 2023 (8)<br><br>To investigate the effects of finerenone in persons with stage 4 CKD.	**Study Design:** FIDELITY subgroup analysis combined data from Fidelio-DKD and Figaro-DKD (N > 13000), 2 phase 3 randomized, double-blind, placebo controlled, multi-center trials.<br><br>**Participants:** N = 870 (n= 440 finerenone and n= 450 placebo) with CKD stage 4 with T2D on maximally tolerated ACEi or ARB based on stage of CKD (stage 3 eGFR < 30 or stage 1-3 eGFR ≥30 mL/min/1.73m^2) at baseline.	**Methodology:** Patients were randomized 1:1 to finerenone (10 or 20 mg once daily based on eGFR) or placebo and grouped based on CKD stage.<br><br>**Analysis:** Primary endpoint was cardiovascular outcome (cardiovascular death, nonfatal myocardial infarction, nonfatal stroke or hospitalization for heart failure), kidney outcome (kidney failure, sustained ≥ 57% decrease in eGFR from baseline or kidney disease death). Safety outcome: AEs including K+.	**Findings:** Mean eGFR for participants was 26.9 ml/min/1.73m^2. The cardiovascular composite outcome occurred in 75(17%) and 92 (20%) in stage 4 CKD who received finerenone compared to placebo (HR 0.78; 95% CI, 0.95-1.07) and was consistent between CKD subgroups. The kidney composite outcome was not consistent over time. Hyperkalemia was the most reported AE occurring in 26% for finerenone compared to 13% for placebo.<br><br>**Drugs to Avoid or Adjust:** Finerenone
Kim et al., Korea, 2024 (9)	**Study Design:** Retrospective observational study	**Methodology:** New users of PPI and H2RA were collected from the Korea National Health Insurance Service database.	**Findings:** PPI initiation was more significantly associated with higher ESKD risk compared to H2RA (aHR 1.72; 95% CI, 1.19-2.48). Risks of

To evaluate and compare the risk adverse effects of PPIs to H2RAs in patients with CKD.	(Jnaur 2009 – December 2017).<br><br>**Participants:** N = 4128 with CKD (eGFR < 60 mL/min/1.73m^2 or dipstick positive albuminuria of >1+ on ≥2 consecutive tests) prescribed PPI/H2RA.	Follow-up occurred one year from index date with initiation of PPIs or H2RA.<br><br>**Analysis:** Primary endpoint was mortality, ESKD, myocardial infarction and stroke. Hazard ratios of outcomes were measured using Cox regression model after adjusting for multiple variables.	mortality and cardiovascular outcomes were similar between 2 groups. In subgroup analysis, association between PPI and progression to ESKD remained significant in eGFR < 60 mL/min/1.73m^2 groups (aHR 1.63; 95% CI, 1.09 – 2.43).<br>**Drugs to Avoid or Adjust:**<br>PPIs
Jain et al.,<br>2023 (10)<br><br>To assess association of adverse effects of PPIs on the renal system.	**Study Design:**<br>Data mining algorithm of FAERS from January 1,2004 to December 31, 2021.<br><br>**Participants:** Patients with suspect renal adverse events with PPIs.	**Methodology:** Data was collected from the FAERS database for PPIs (rabeprazole, pantoprazole, lansoprazole, and omeprazole) and suspected CKD, AKI, RF, RI and ESRD.<br><br>**Analysis:** Primary endpoint was disproportionate reporting of renal adverse effects from PPIs using data mining algorithms such as proportional reporting ratio e.g., PRR (≥2) with associated chi-squared value (>4), reporting odds ratio e.g., ROR (≥2) with 95% CI and case count (≥3) were calculated to identify a possible signal.	**Findings:** CKD was the most common reported adverse event with PPIs in the FAERS database. The calculated PRR and ROR indicated a positive signal of suspected CKD, AKI, RF, RI and ESRD with PPIs. The subgroup analysis results revealed a greater number of cases in the age group (18–64 years compared to other age groups. Polypharmacy did decrease the number of reports associated with CKD, AKI, RF, RI and ESRD but did not change the overall analysis.<br>**Drugs to Avoid or Adjust:**<br>PPIs (Rabeprazole, pantoprazole, lansoprazole and omeprazole)
Killam-Worrall et al., United States, 2024 (11)<br><br>To review adverse effects of baclofen and tizanidine in older community dwelling adults.	**Study Design:**<br>Systematic review (July 2019- June 2023). FAERS public dashboard used to capture AEs of interest not captured in included studies.<br><br>**Participants:**<br>Age > 50 years taking baclofen or tizanidine.<br>**Setting:**<br>Community dwelling.	**Methodology:** Systematic search of Web of Science, Scopus, Embase, and PubMed to identify studies with adverse events (falls, fractures, confusion, drowsiness, gait disturbances) associated with baclofen or tizanidine. Data from FAERS was extracted for AEs of interest for baclofen and tizanidine.<br><br>**Analysis:** Data was extracted from include studies. FAERS data were compiled for adverse effect incidence.	**Findings:**<br>Ten records reported baclofen-related adverse effects of dyskinesia, encephalopathy, and drowsiness. The other 5 studies reported tizanidine-related adverse effects of bradycardia and hypotension. Eight of the 15 studies included individuals with renal dysfunction. Two studies showed increased hospitalization for encephalopathy in CKD (7.2% versus 0.1%) and ESRD (daily dose baclofen 20 mg or more; RR 19.8; 95% CI, 14-28). FAERS revealed 486 (baclofen) and 305 (tizanidine) AEs of interest, with a 27.8% and 29% incidence of falls for baclofen and tizanidine.<br>**Drugs to Avoid or Adjust:**<br>Tizanidine (Baclofen include)
Switzer et al.,	**Study Design:**<br>Narrative review of	**Methodology:** Systematic review of national institute of health, US	**Findings:** Three articles of two randomized controlled studies were

United State, 2022 (12)<br><br>To review efficacy safety of atogepant for migraine prevention.	RCTs of atogepant (February 1, 2012 – February 1, 2022).<br><br>**Participants:** N = 796 (Three articles from 2 RCTs), adults with ≥ 1 year history of migraine.	National Library of Medicine Clinical Trials, PubMed, European PMC and Cochrane Library databases for efficacy/safety of atogepant compared to placebo for prevention of episodic migraines.<br><br>**Analysis:** Primary endpoint was change in MMD from baseline to week 12. Safety included TEAEs for atogepant compared to placebo.	included. MMD at 4 weeks showed significant improvement for all doses compared to placebo (p <0.0018). No serious TEAEs were reported. Patients with CrCl < 30 mL/min or end-stage renal disease were not included in any of the studies; safety is unknown. Physiologically based PK model predicted that atogepant exposures increase by about 2.3-fold in patients with severe renal impairment.<br>**Drugs to Avoid or Adjust:** Atogepant (probable)
Stern et al., 2023, USA (13)<br><br>To review migraine management (abortive and prevention) in person with renal or hepatic disease.	**Study Design:** Narrative review.<br><br>**Participants:** Adults aged 19 -44 years.	**Methodology**: Literature search of PubMed, Ovid Medline, Embase and Cochrane Library for migraine or headache and renal or hepatic disease. Product labels reviewed as well as drug databases (Renal Drug Handbook).<br><br>**Analysis:** Relevant articles were screened by two authors.	**Findings:** For acute migraine treatment in eGFR 15-29 mL/min/1.73m^2, use half the dose no more than 50 mg/dose Ubrogepant and may use second 50 mg dose after 2 hours. For migraine prevention and eGFR 15-29 mL/min/1.73m^2, maximum 10 mg atogepant daily. In ESRD, atogepant and Ubrogepant have not been studied and safety is unknown.<br>**Drugs to Avoid or Adjust:** Atogepant, Ubrogepant.
Wang et al., 2022, United States (14)<br><br>To evaluate the effects of renal impairment on the pharmacokinetics of abrocitinib.	**Study Design:** Phase I, nonrandomized, open label, single dose, parallel-cohort study.<br><br>**Participants:** N = 23 Age 18-75 years. Normal renal function: eGFR ≥ 90 mL/min (n= 8) Moderate renal impairment: eGFR ≥30 to <60 mL/min (n=7) Severe renal impairment: eGFR < 30 mL/min (n=8)	**Methodology** Blood samples were collected up to 72 hours after a single 200 mg dose of abrocitinib.<br><br>**Analysis:** PK parameters were calculated using standard noncompartmental analysis including $AUC_{0\rightarrow inf}$, Cmax, tmax and terminal half-llife, and apparent oral clearance (CL/F) for abrocitinib and its metabolites (M1, M2 and M4).	**Findings:** For abrocitinib, the adjusted geometric mean ratios (GMRs; %) for $AUC_{0\rightarrow inf}$ were 182.91 (90% CI, 117.09-285.71) and 138.49 (90% CI, 93.74-204.61), respectively, for subjects with moderate renal impairment vs normal renal function; corresponding GMRs were 121.32 (90% CI, 68.32-215.41) and 99.11 (90% CI, 57.30-171.43) for subjects with severe impairment vs normal renal function. Metabolite exposures generally increased in subjects with renal impairment. Moderate and severe renal impairment leads to higher exposure to abrocitinib, suggesting dose should be reduced by half.<br>**Drugs to Avoid or Adjust:** Abrocitinib
O'Dwyer et al., 2024, United States (15)	**Study Design:** Post-hoc analysis of phase III, open-label safety study.	**Methodology:** A subset of 10 US study sites enrolling 11 or more patients with high-quality seizure data.	**Findings:** At 36 months, 26.2% of older patients discontinued treatment due to AEs. The most common were dizziness, fall and balance disorders (≥10% incidence). Gait disturbances

To assess safety and efficacy of cenobamate in older patients.	**Participants:** N = 15 Age 65-70 years with uncontrolled focal seizures.	**Analysis:** Primary endpoint: Percentage of patients achieving 100% reduction in seizures within 3-month intervals during maintenance phase and at last 3-month visit (up to 24 months). Discontinuation due to adverse effects and treatment emergent adverse effects were assessed in persons who received 1 or more doses of cenobamate.	were also more common in older patients compared to the original safety study (11.9% compared to 5.4%). Kidney function was not available. Cenobamate is extensively metabolized, and its metabolites are primarily excreted renally. A reduction in dose is recommended when creatinine clearance is ≤ 90 mL/min, and cenobamate is not recommended in end-stage renal disease. **Drugs to Avoid or Adjust:** Cenobamate

ACEi = angiotensin-converting enzyme inhibitor, AE = adverse effects, aHR = adjusted hazard ratio, AKI = acute kidney injury, ARB = angiotensin receptor blocker, AUC = area under curve, CKD = chronic kidney disease, CLr = renal clearance, CrCl = creatinine clearance, eGFR = estimated glomerular filtrate rate, ESKD = end-stage kidney disease, ESRD = end-stage renal disease, FAERS = FDA adverse reporting system, H2RA = histamine type 2 receptor antagonist, IPDMA = individual data meta-analysis, MD = mean difference, MMD = monthly migraine days, MTX = methotrexate, OR = odds ratio, PK = pharmacokinetic, PPI = proton pump inhibitor, PrEP = pre-exposure prophylaxis, PRR = proportional reporting ratio, RD = risk difference, RF = renal failure, RI = renal injury, ROR = reporting odds ratio, RR = relative risk, SCr = serum creatinine, TDF = tenofovir disoproxil fumarate, TEAEs = treatment-emergent adverse effects

References

1. Petruccelli KCS, Baía-da-Silva DC, Val F, et al. Kidney function and daily emtricitabine/tenofovir disoproxil fumarate pre-exposure prophylaxis against HIV: results from the real-life multicentric demonstrative project PrEP Brazil. AIDS Res Ther. 2022;19(1):12. Available from: https://doi.org/10.1186/s12981-022-00437-4
2. Schaefer R, Amparo da Costa Leite PH, Silva R, et al. Kidney function in tenofovir disoproxil fumarate-based oral pre-exposure prophylaxis users: a systematic review and meta-analysis of published literature and a multi-country meta-analysis of individual participant data. Lancet HIV. 2022;9(4):e242-e253. Available from: https://doi.org/10.1016/s2352-3018(22)00004-2
3. Toussi SS, Neutel JM, Navarro J, et al. Pharmacokinetics of oral nirmatrelvir/Ritonavir, a protease inhibitor for treatment of COVID-19, in subjects with renal impairment. Clin Pharmacol Ther. 2022; 112(4):892-900. Available from: https://doi.org/10.1002/cpt.2688
4. Chan GCK, Lui GCY, Wong CNS, et al. Safety profile and clinical and virological outcomes of nirmatrelvir-ritonavir treatment in patients with advanced chronic kidney disease and coronavirus disease 2019. Clin Infect Dis. 2023;77(10):1406-1412. Available from: https://doi.org/10.1093/cid/ciad371
5. Muanda FT., Sood MM., Weir MA., Sontrop JM., Ahmadi F., Yoo E., et al. Association of higher-dose fluoroquinolone therapy with serious adverse events in older adults with advanced chronic kidney disease. JAMA Netw Open. 2022 Aug; 5(8): e2224892. Available from: https://doi.org/10.1001/jamanetworkopen.2022.24892
6. Muanda FT., Blake PG., Weir MA., Ahmadi F., McArthur E., Sontrop JM., et al. Low-dose methotrexate and serious adverse events among older adults with chronic kidney disease. JAMA Netw Open. 2023 Nov; 6(11): e2345132. Available from: https://doi.org/10.1001/jamanetworkopen.2023.45132
7. Hu H, Cao M, Sun Y, Jin X, Zhao X, Cong X. Efficacy and Safety of Eplerenone for Treating Chronic Kidney Disease: A Meta-Analysis. Int J Hypertens. 2023 Mar 9;2023:6683987. Available from: https://doi.org/10.1155%2F2023%2F6683987
8. Sarafidis P, Agarwal R., Pitt B., Wanner C., Filippatos G., Boletis J., et al. on behalf of the FIDELIO-DKD and FIGARO-DKD Investigators. Outcomes with finerenone in participants with stage 4 CKD and type 2 Diabetes: A FIDELITY

subgroup analysis. Clinical Journal of the American Society of Nephrology. 2023 May; 18(5): 602-612. Available from: http://doi.org/10.2215/CJN.0000000000000149

9. Kim SG, Cho JM, Han K, Joo KW, Lee S, Kim Y, Cho S, Huh H, Kim M, Kang E, Kim DK, Park S. Non-indicated initiation of proton pump inhibitor and risk of adverse outcomes in patients with underlying chronic kidney disease: a nationwide, retrospective, cohort study. BMJ Open. 2024 Jan 29;14(1):e078032. Available from: https://doi.org/10.1136/bmjopen-2023-078032
10. Jain D., Sharma G., Kumar A. Adverse effects of proton pump inhibitors (PPIs) on the renal system using data mining algorithms (DMAs). Expert opinion on drug safety. 2023 Mar 22; 22(8): 741-752. Available from: https://doi.org/10.1080/14740338.2023.2189698
11. Killam-Worrall L, Brand R, Castro JR, Patel DS, Huynh K, Lindley B, Torres BP. Baclofen and Tizanidine Adverse Effects Observed Among Community-Dwelling Adults Above the Age of 50 Years: A Systematic Review. Ann Pharmacother. 2024 May;58(5):523-532. Available from: https://doi.org/10.1177/10600280231193080
12. Switzer MP, Robinson JE, Joyner KR, Morgan KW. Atogepant for the prevention of episodic migraine in adults. SAGE Open Med. 2022;10. Available from: https://doi.org/10.1177/20503121221128688
13. Stern JI., Datta S., Chiang C-C., Garza I., Vieira DL., Robertson CE. Narrative review of migraine management in patients with renal or hepatic disease. Headache Currents. 2023 Jan 28; 63: 9-24. Available from: https://doi.org/10.1111/head.14437
14. Wang EQ, Le V, Winton JA, et al. Effects of Renal Impairment on the Pharmacokinetics of Abrocitinib and Its Metabolites. J Clin Pharmacol. 2022;62(4):505-519. Available from: http://doi.org/10.1002/jcph.1980
15. O'Dwyer R, Stern S, Wade CT, Guggilam A, Rosenfeld WE. Safety and Efficacy of Cenobamate for the Treatment of Focal Seizures in Older Patients: Post Hoc Analysis of a Phase III, Multicenter, Open-Label Study. Drugs Aging. 2024;41(3):251-260. Available from: http://doi.org/10.1007/s40266-024-01102-3
