# Supplementary material for: Medications for community pharmacists to dose adjust or avoid to enhance prescribing safety in individuals with advanced chronic kidney disease: a scoping review and modified Delphi
Source: BMC Nephrol. 2024 Oct 29;25:386. doi: 10.1186/s12882-024-03829-y (PMC11523796; doi:10.1186/s12882-024-03829-y)
Supplement: Supplementary file 6 — Additional file 6: Medications Selected to Dose Adjust by all Participants for Round 1 and 2. [file 12882_2024_3829_MOESM6_ESM.pdf]

Additional File 6. Medications Selected to Dose Adjust by All Participants from Round 1 and 2

eGFR 15-29 mL/min	eGFR < 15 mL/min
Sitagliptin	Rosuvastatin
Rosuvastatin	Gabapentin
Solifenacin	Pregabalin
Tolterodine	Topiramate
Gabapentin	Allopurinol
Pregabalin	Famciclovir
Topiramate	Oseltamivir
Allopurinol	Ciprofloxacin
Colchicine	Norfloxacin
Enoxaparin	Fluconazole
Acyclovir	Venlafaxine
Famciclovir	Metoclopramide
Valacyclovir	Tizanidine
Oseltamivir	Sildenafil
Amoxicillin	Memantine
Amoxicillin / Clavulanic Acid	
Cephalexin	
Clarithromycin	
Sulfamethoxazole / Trimethoprim	
Ciprofloxacin	
Norfloxacin	
Fluconazole	
Ranitidine	
Famotidine	
Venlafaxine	
Metoclopramide	
Digoxin	
Tizanidine	
Risperidone	
Sildenafil	
Amantadine	
Memantine	
