# Supplementary material for: Medications for community pharmacists to dose adjust or avoid to enhance prescribing safety in individuals with advanced chronic kidney disease: a scoping review and modified Delphi
Source: BMC Nephrol. 2024 Oct 29;25:386. doi: 10.1186/s12882-024-03829-y (PMC11523796; doi:10.1186/s12882-024-03829-y)
Supplement: Supplementary file 7 — Additional file 7: Top 40 Medication List Selected by Modified Delphi Panel. [file 12882_2024_3829_MOESM7_ESM.pdf]

Additional File 7. Top 40 Medications Selected by Modified Delphi Panel

Count	Medication	Percentage (n = 26)
1	Metformin	100
2	Gabapentin	100
3	Pregabalin	100
4	Rivaroxaban	100
5	Ciprofloxacin	100
6	Nitrofurantoin	100
7	NSAIDs	100
8	Edoxaban	96.15
9	Levofloxacin	96.15
10	Digoxin	92.31
11	Glyburide	92.31
12	Colchicine	92.31
13	Dabigatran	92.31
14	Acyclovir	92.31
15	Famciclovir	92.31
16	Valacyclovir	92.31
17	Paxlovid[1]	92.31
18	Sulfamethoxazole / Trimethoprim	92.31
19	Baclofen	88.46
20	Enoxaparin	88.46
21	Truvada[2]	88.46
22	Norfloxacin	88.46
23	Tramadol	88.46
24	Lithium	84.62
25	Sotalol	84.62
26	Fibrates	84.62
27	Allopurinol	84.62
28	Apixaban	84.62
29	Clarithromycin	84.62
30	Codeine	84.62
31	Duloxetine	84.62
32	Bupropion	84.62
33	Topiramate	80.77
34	Fluconazole	80.77
35	Dalteparin	80.77
36	Morphine	76.92
37	Methotrexate	76.92
38	Varenicline	76.92
39	Amantadine	76.92
40	Oseltamivir	73.08

[1] Nirmatrelvir/Ritonavir [2] Emtricitabine/Tenofovir Disoproxil Fumarate
